# Design of Cobalt Fischer–Tropsch Catalysts for the Combined Production of Liquid Fuels and Olefin Chemicals from Hydrogen-Rich Syngas

**DOI:** 10.1021/acscatal.0c05027

**Published:** 2021-04-05

**Authors:** Kai Jeske, Ali Can Kizilkaya, Iván López-Luque, Norbert Pfänder, Mathias Bartsch, Patricia Concepción, Gonzalo Prieto

**Affiliations:** † Max-Planck-Institut für Kohlenforschung, Kaiser-Wilhelm-Platz 1, 45470 Mülheim an der Ruhr, Germany; ‡ Department of Chemical Engineering, Izmir Institute of Technology, Gülbahçe Kampüsü, 35430 Izmir, Turkey; § ITQ Instituto de Tecnología Química, Universitat Politècnica de València-Consejo Superior de Investigaciones Científicas (UPV-CSIC), Avenida de los Naranjos s/n, 46022 Valencia, Spain; ∥ Max-Planck-Institut für Chemische Energiekonversion, Stiftstraße, 45470 Mülheim an der Ruhr, Germany; ⊥ Faculty of Physics and CENIDE, Universität Duisburg-Essen, 47048 Duisburg, Germany

**Keywords:** hierarchical porosity, promotion effects, linear olefins, (bio) syngas, DFT calculations

## Abstract

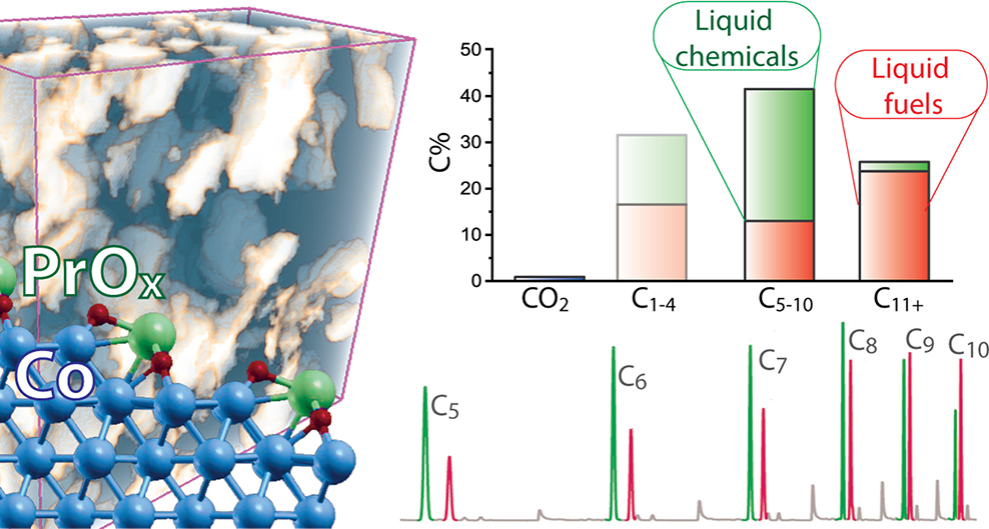

Adjusting hydrocarbon product distributions in the Fischer–Tropsch (FT) synthesis is of notable significance in the context of so-called X-to-liquids (XTL) technologies. While cobalt catalysts are selective to long-chain paraffin precursors for synthetic jet- and diesel-fuels, lighter (C_10–_) alkane condensates are less valuable for fuel production. Alternatively, iron carbide-based catalysts are suitable for the coproduction of paraffinic waxes alongside liquid (and gaseous) olefin chemicals; however, their activity for the water–gas-shift reaction (WGSR) is notoriously detrimental when hydrogen-rich syngas feeds, for example, derived from (unconventional) natural gas, are to be converted. Herein the roles of pore architecture and oxide promoters of Lewis basic character on CoRu/Al_2_O_3_ FT catalysts are systematically addressed, targeting the development of catalysts with unusually high selectivity to liquid olefins. Both alkali and lanthanide oxides lead to a decrease in *turnover frequency*. The latter, particularly PrO_*x*_, prove effective to boost the selectivity to liquid (C_5–10_) olefins without undesired WGSR activity. *In situ* CO-FTIR spectroscopy suggests a dual promotion via both electronic modification of surface Co sites and the inhibition of Lewis acidity on the support, which has direct implications for double-bond isomerization reactivity and thus the regioisomery of liquid olefin products. Density functional theory calculations ascribe oxide promotion to an enhanced competitive adsorption of molecular CO versus hydrogen and olefins on oxide-decorated cobalt surfaces, dampening (secondary) olefin hydrogenation, and suggest an exacerbated metal surface carbophilicity to underlie the undesired induction of WGSR activity by strongly electron-donating alkali oxide promoters. Enhanced pore molecular transport within a multimodal meso-macroporous architecture in combination with PrO_*x*_ as promoter, at an optimal surface loading of 1 Pr_at_ nm^–2^, results in an unconventional product distribution, reconciling benefits intrinsic to Co- and Fe-based FT catalysts, respectively. A chain-growth probability of 0.75, and thus >70 C% selectivity to C_5+_ products, is achieved alongside lighter hydrocarbon (C_5–10_) condensates that are significantly enriched in added-value chemicals (67 C%), predominantly α-olefins but also linear alcohols, remarkably with essentially no CO_2_ side-production (<1%). Such unusual product distributions, integrating precursors for synthetic fuels and liquid platform chemicals, might be desired to diversify the scope and improve the economics of small-scale gas- and biomass-to-liquid processes.

## Introduction

The widespread availability of (unconventional) natural gas resources make gas-to-liquid (GTL) technologies an attractive alternative to current refining, which relies on centralized and dwindling crude oil supplies, for the production of fuels and chemicals.^1,2^ The Fischer–Tropsch (FT) synthesis forms the core of GTL processes, enabling the valorization of natural gas into synthetic hydrocarbon fuels and specialty lubricants via syngas (CO+H_2_) as a versatile intermediate.^3^ In a future scenario where personal and short-distance transport becomes largely electrified, synthetic GTL fuels are expected to play a central role in the heavy-duty ground transport and aviation sectors, where volumetric energy density considerations make liquid fuels a nearly irreplaceable choice.

Long-chain FT *n*-paraffin products (C_11+_) are excellent precursors for sulfur-free jetfuels–via hydroisomerization of the C_11–16_ fraction^4,5^—and high-cetane diesel fuels—via hydrocracking of heavier waxes.^6^ However, the broad statistical (Anderson–Schulz–Flory)^7^ hydrocarbon product distribution inherent to this polymerization reaction inevitably results in the coproduction of hydrocarbon fractions of lower value as fuel precursor. This includes tail-gases (C_1–4_) but also a significant fraction (typically >35 wt %) of condensable C_5–10_ alkanes, which are not suitable precursors for the aforementioned synthetic fuels because of their low boiling (<450 K) and flash (<320 K) points.

The FT reaction mechanism involves the surface polymerization of unbranched hydrocarbons from monocarbonated species, followed by chain-termination via either β-H-elimination or α-H-addition hydrogenation, leading to the desorption of α-olefin or *n*-paraffin products, respectively. Studies on model 2D catalysts have shown the former termination pathway to be favored and thus α-olefins to be the major primary reaction products.^8^ However, in technical porous catalysts, the final FT product mixture often does not reflect this intrinsic surface kinetics, and the condensable products are largely enriched in *n*-paraffins. After primary desorption, α-olefin products might readsorb on the catalyst surface and undergo secondary reactions, primarily hydrogenation to the corresponding *n*-paraffins but also chain-reinsertion and double-bond migration into internal isomers.^9,10^ This secondary processing is known to be pore-transport-enhanced, and thus, its extent depends not only on the reactivity of the catalyst surface but also on the pore residence time of olefin products as they egress by diffusion to the continuous phase.^11^ Favoring primary α-olefins over secondary *n*-paraffins products would add value to the FT lighter condensate (C_5–10_) slate.

Liquid linear α-olefins (LAO) are valuable commodity chemicals. LAO in the C_5–10_ range find applications as polymer comonomers, alkylating agents for the production of alkylbenzenes, precursors of specialty lubricants (via oligomerization) and raw materials for the synthesis of low-MW fatty acids, organosilanes and thiols for functional coatings,^12−14^ amines,^15^ as well as aldehyde and alcohol solvents and plasticizers via hydroformylation with syngas.^16^ Industrially, C_5–10_ LAO are largely produced by ethylene oligomerization, which yields mostly even-numbered hydrocarbon chains.^17,18^ Alternative production routes involve *n*-paraffin dehydrogenation, wax cracking, alcohol dehydration, or fatty acid decarboxylation/ethenolysis but are less widespread industrially.^19,20^ The coproduction of synthetic C_5–10_ α-olefins concomitantly to C_11+_ paraffinic diesel and jetfuel precursors in GTL processes could provide a future-proof source of two high-demand commodities from syngas. Moreover, downstream conversion of the former to C_6–11_*n*-alcohols via hydroformylation enables the formulation of high-cetane, drop-in syngas-derived fuels which exploit the remarkable soot inhibition properties of middle-chain *n*-alcohols.^21,22^

However, there are currently no specific catalysts leading to such unconventional product distribution from hydrogen-rich syngas (H_2_/CO ∼ 2). FeC_*x*_-based FT catalysts provide high olefin selectivities, particularly in the C_10–_ fraction. However, their prominent activity for the water–gas-shift reaction (WGSR) leads to the rejection of a significant share of the oxygen in the syngas feed in the form of CO_2_.^23,24^ While this feature is valuable to convert syngas feeds with substoichiometric H_2_/CO ratios (0.7–1.2), for example, derived from coal gasification,^25^ it encumbers their use to process syngas derived from natural gas reforming or biomass steam gasification,^26,27^ with higher H_2_ contents (H_2_/CO ∼ 2). In the latter cases, the coproduction of CO_2_ would entail lower conversion efficiencies per-pass and significant tail-gas recycle compression costs.^28^ As a result, oxide-supported cobalt nanoparticles are the catalysts of choice in high-yield GTL technologies because of their high specific reaction rates at mild temperature (∼473 K), high selectivity to waxes, and intrinsic inactivity for the WGSR.^29^ However, their remarkable (secondary) hydrogenation reactivity efficiently depletes primary α-olefins, resulting in essentially paraffinic products. The design of CoMnO_*x*_^30−32^ catalysts has recently unlocked high selectivities to light (C_2–4_) olefins, remarkably, after dual promotion with Na and S, with very low WGSR activity.^32^ However, chain growth probabilities ≤0.5 limit their selectivity to liquid products.

Designing the pore architecture of Co-based FT catalysts to achieve fast pore transport rates has proven effective to minimize secondary hydrogenation and boost the share of liquid olefins in the products. This strategy proved useful to curb undesired overcracking in tandem FT/hydrocracking processes for the direct conversion of syngas into wax-free liquid hydrocarbons.^33,34^ The combination of pore transport enhancement with surface promotion effects could provide a blueprint toward unconventional FT product distributions uniting high selectivities to C_5–10_ α-olefin chemicals and heavier paraffinic fuel precursors while retaining the essentially null CO_2_ side-production inherent to cobalt-based FT catalysts. Here we address the combined effect of porosity and Lewis basic oxide promoters to develop γ-Al_2_O_3_-supported cobalt FT catalysts with high selectivity to liquid C_5–10_ olefins from H_2_-rich syngas.

## Experimental Section

### Synthesis of CoRu/γ-Al_2_O_3_ Catalysts

Meso-macroporous γ-Al_2_O_3_ was synthesized via soft-templating from an aqueous gel incorporating pseudoboehmite (75% Al_2_O_3_, Disperal P2, Sasol) and a polyethylene glycolether nonionic surfactant (Tergitol 15-S-7) as porogen (Al:EO:H_2_O = 1:8.1:49, EO: ethylene oxide subunits in the surfactant), followed by drying the mixture at 343 K for 72 h, and 393 K for 3 h, and subsequent calcination at 823 K (0.5 K min^–1^) in stagnant air. Mesoporous γ-Al_2_O_3_ supports were obtained by dehydration of high-purity pseudoboehmite precursors (Sasol Materials). CoRu/γ-Al_2_O_3_ catalysts were then synthesized on sieve fractions (0.2–0.4 mm) of the γ-Al_2_O_3_ supports via incipient wetness impregnation with a solution of Co(NO_3_)_2_·6H_2_O (1.5 M) and ruthenium(III) nitrosyl nitrate (Ru/Co = 0.007) in dilute nitric acid. After they were dried at 343 K under Ar flow (200 cm^3^ g_cat_^–1^) for 10 h, the precursors were decomposed by heating to 623 K for 4 h (1 K min^–1^) in a vertical downward flow reactor. Promoted CoRu/γ-Al_2_O_3_ catalysts were synthesized by wet impregnation with nitrate precursors of the respective promoter element dissolved in 0.5 M HNO_3_, followed by removal of H_2_O in a rotary evaporator (323 K) and calcination in air flow (200 cm^3^ g_cat_^–1^) for 4 h at 623 K. Catalysts were denoted *x*M-CoRu/AOm(*p*), where M indicates the identity of the promoter in the case of promoted catalysts and *x* specifies its surface specific content (M_at_ nm^–2^). AOm or alternatively AOmM designate either monomodal mesoporous or multimodal meso-macroporous γ-Al_2_O_3_ supports, respectively. For the former, *p* additionally denotes the average mesopore diameter.

### Characterization Methods

N_2_ physisorption isotherms were recorded at 77 K using a Micromeritics 3Flex V4.04 device after degassing at 523 K under vacuum for 12 h. Hg intrusion porosimetry was performed in a Micromeritics AutoPore IV 951 apparatus after the sample (0.2–0.4 mm particles) was dried at 383 K for 72 h. H_2_ chemisorption isotherms were recorded at 373 K using an ASAP 2010C (Micromeritics) after *in situ* reduction in flowing H_2_ at 673 K for 5 h (2 K min^–1^) and degassing the sample at 1.3 Pa. High-angle annular dark-field scanning-transmission electron microscopy was performed with a spherical aberration-corrected beam (C_s_) Hitachi HD-2700 microscope equipped with a cold field-emission gun and two EDAX Octane T Ultra W EDS detectors, operated at 200 kV. Samples were embedded in a low-viscosity resin, sectioned to ∼150 nm thick slices using a Reichert Ultracut ultramicrotome, and collected on Cu TEM grids (300 mesh) supporting a lacey carbon film. X-ray photoelectron spectra were recorded on a SPECS spectrometer with a Phoibos 150 MCD-9 detector and a nonmonochromatic (AlKα = 1486.6 eV) X-ray source after *in situ* reduction of pelletized samples under H_2_ flow at 673 K, and in certain instances exposure to FT reaction conditions *in situ* (T = 473 K, P = 10 bar, H_2_/CO = 2), followed by *in vacuo* transfer to the photoelectron spectroscopy chamber. FTIR spectroscopy experiments were performed in reactor cells featuring KRS-5 windows and mounted on a Bruker Vertex70 spectrometer. CO was applied as surface probe molecule at 298 and 110 K in order investigate metallic and oxide surface Lewis sites, respectively, on the *in situ* reduced catalysts. Prior to XPS and CO-FTIR experiments, catalysts had been reduced *ex situ* in H_2_ (70 cm^3^ min^–1^) at 673 K for 5 h (heating rate 1 K min^–1^), followed by a metal passivation treatment in flow of 1% O_2_/N_2_ for 1 h at room temperature to limit *in situ* reduction in the cells to the passivation overlay. CO_2_-TPD-MS was carried out on a Micromeritics TPD/2900 connected to a quadrupole mass spectrometer (Pfeiffer) after *in situ* reduction under flow of 10% H_2_/Ar (50 cm^3^ min^–1^) at 673 K for 2 h (heating rate 10 K min^–1^).

### Catalysis

Catalytic experiments were performed in a fixed-bed high-Cr 316 stainless steel reactor loaded with 0.2–0.4 mm catalyst particles diluted with SiC granules (46 grit). Prior to reaction, the catalyst was reduced *in situ* under a flow of H_2_ at (200 cm^3^ min^–1^) at 673 K (2 K min^–1^ to 423 K, followed by 0.83 K min^–1^ to 673 K) for 5 h at atmospheric pressure. The reaction was carried out at 473 K and 20 bar using a feed with molar composition 30% CO/60% H_2_/10% Ar (Ar as internal GC standard). The stream leaving the reactor was depressurized and periodically analyzed online with a GC (Agilent 7890B) equipped with two TCD detectors and one FID detector, while liquid and solid hydrocarbons were collected in high-pressure traps, and analyzed offline. Product selectivities are reported on a carbon basis in the pseudosteady state, that is, after at least 16 h on-stream at the corresponding feed space velocity (WHSV).

### First-Principles Density Functional Theory (DFT) Calculations

Periodic DFT calculations were performed using the Vienna Ab-initio Simulation Package (VASP).^35,36^ The exchange-correlation energy was calculated with the PBE^37^ form of the generalized gradient approximation (GGA) functional. The electron–ion interaction was modeled by the projector-augmented wave (PAW)^38^ method. Spin-polarized calculations were performed to account for the magnetic properties of cobalt with a plane wave cutoff energy of 600 eV.

Additional experimental and computational details are provided in the Supporting Information.

## Results and Discussion

### Catalyst Design

**Figure 1:**
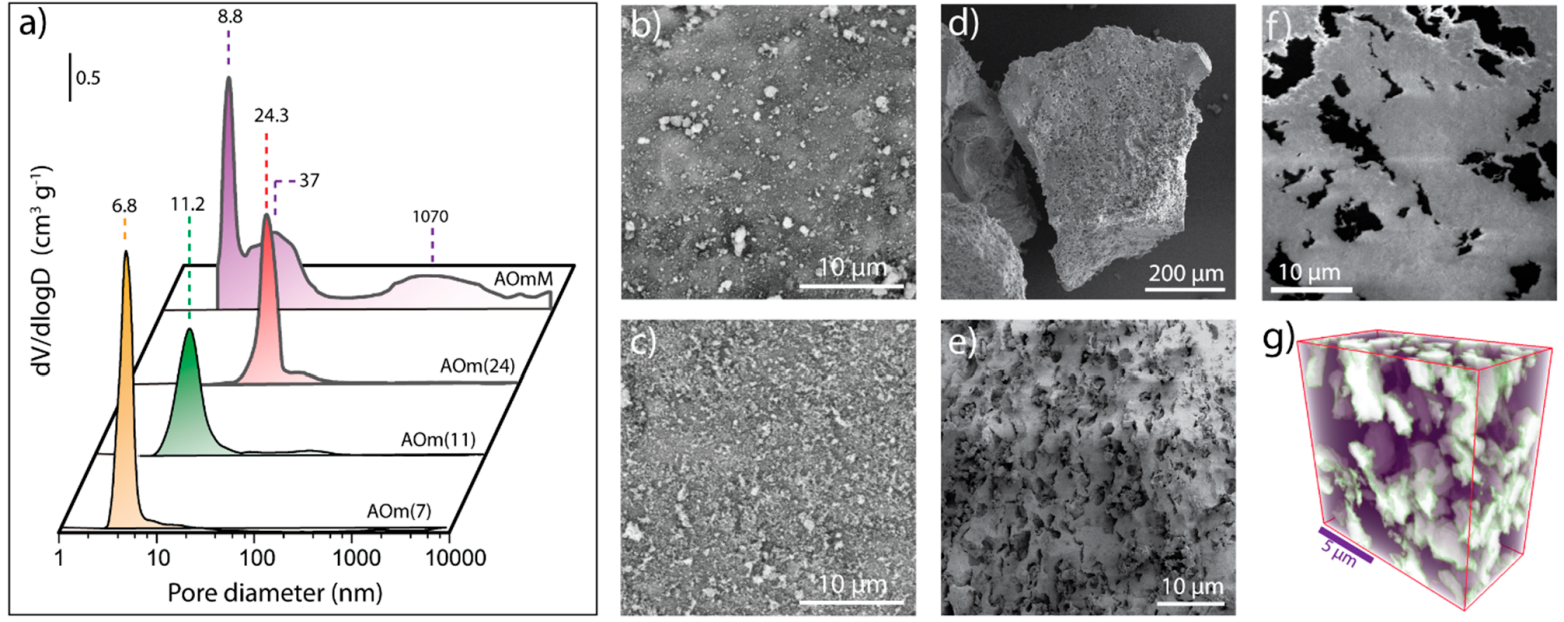
Porosity assessment for γ-Al_2_O_3_ support materials. (a) Pore size distributions as derived by Hg intrusion porosimetry for the series of γ-Al_2_O_3_ support materials. (b,c) Representative SEM micrographs for microparticles of AOm(11) and AOm(24), respectively, showing no signs of macropores on their outer surface; (d,e) Representative scanning electron micrographs for microparticles of AOmM showing the percolation of macropores to the outer surface. (f) Cross-sectional SEM micrograph after focus-ion-beam (FIB) milling of the resin-embedded AOmM support. Lighter gray regions correspond to mesoporous Al_2_O_3_ regions, while dark gray patches correspond to macropores cross sections. (g) 3D-rendered view of a reconstructed FIB-SEM tomogram for AOmM. Purple regions correspond to mesoporous Al_2_O_3_ and white regions to intraparticle macropores.

The porosity of the carrier material is known to play a central role for pore mass transport phenomena and selectivity in the cobalt-catalyzed Fischer–Tropsch synthesis.^9,39^ Table S1 in the Supporting Information summarizes the textural properties determined for the series of γ-Al_2_O_3_ support materials. Their porous structure was assessed by Hg intrusion porosimetry in order to probe the meso- as well as the macropore regimes. Figure 1a shows the corresponding pore size distributions. Monomodal and narrow size distributions peaking at 6.8 and 11.2 nm were observed for AOm(7) and AOm(11), respectively. In the case of the wider-pore AOm(24), the dominant pore population centered at 24.3 nm appeared complemented by a shoulder at 49.6 nm, suggesting the presence of a second, minor population of wider openings. Pore volume contributions in the macropore regime (>50 nm) were essentially negligible in all cases (≤0.05 cm^3^ g^–1^), indicating that this set of materials represents essentially mesoporous supports covering a wide range of pore diameters. A multimodal pore size distribution was ascertained in the case of AOmM, with mesopore modes peaking at 8.8 and 37 nm, respectively, alongside a significant contribution from macropores (0.46 cm^3^ g^–1^) with diameters extending in a wider range centered at around 1 μm. In contrast to the even outer surface observed by SEM for the monomodal mesoporous carriers (Figure 1b,c), macropores were found to protrude to the outer surface of the microparticles in AOmM (Figure 1d,e). Focused-ion-beam scanning-electron-tomography (FIB-SEM) was applied to image the internal macropore architecture of this hierarchically porous support. As observed in the cross-sectional SEM micrograph (Figure 1f) and the corresponding reconstructed tomogram (Figure 1g), irregularly shaped macropores were found to be evenly distributed within the inner volume of the material.

**Figure 2:**
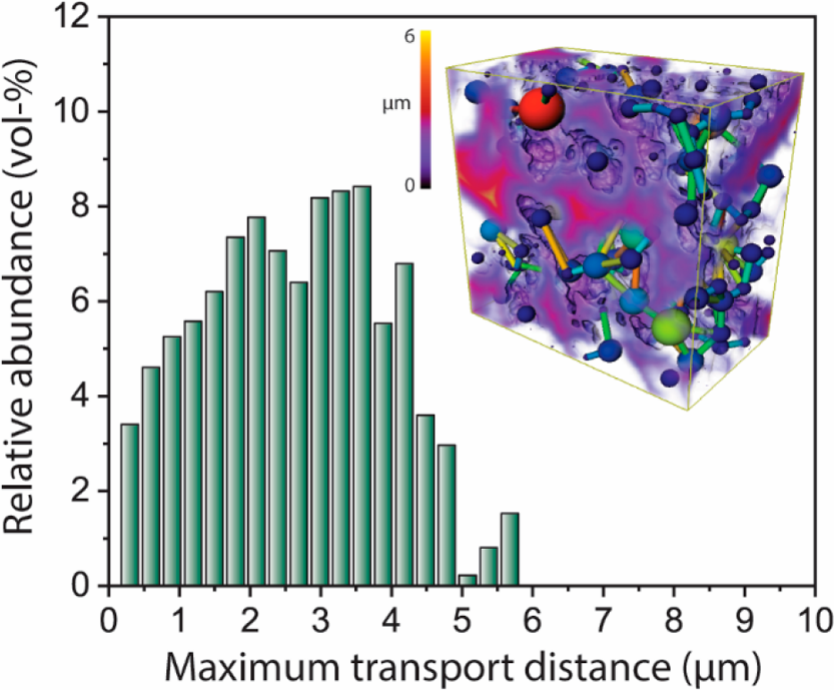
Histogram for the maximum Euclidean distance from mesopore regions to the nearest boundary with the macropore network as derived from 3D image analysis of the reconstructed FIB-SEM tomogram for the bimodally meso-macroporous γ-Al_2_O_3_ support material (AOmM). The inset shows the 3D contour plot for the Euclidean distance to nearest macropore as well as the computed Pore Network Model, with macropores shown as balls and throats connecting them as bars. See details for the 3D tomographic image quantification in the Supporting Information.

Quantitative image analysis of the FIB-SEM tomogram revealed a high degree of connectivity for the macropore network, with an average coordination at the intersecting *nodes* of the macropore network model of 3.1 ± 1.3. More importantly, the network of interconnected macropores results in a significant partitioning of the mesoporous γ-Al_2_O_3_ domains, reducing the average transport distances within mesoporous regions down below 5 μm (Figure 2). Compared with mesoporous domains extending over the entire microparticle diameter (200–400 μm) in the case of the series of strictly mesoporous γ-Al_2_O_3_ supports, these shorter mesopore transport distances are expected to significantly enhance pore molecular transport.^40^

Ruthenium-promoted^41^ cobalt FT catalysts were synthesized on the set of Al_2_O_3_ support materials. In all cases, the nominal cobalt surface-specific loading was set constant to 9.0 ± 1.0 Co_at_ nm^–2^ in order to achieve a uniform surface density of metal sites in the series of catalysts. This translated into Co loadings in the range of 9–22 wt % Co on the set of γ-Al_2_O_3_ carriers which showed BET specific surface areas spanning 96–300 m^2^ g^–1^ (Figure S1, Table S1). As oxide promoters, a series of alkali (Na, K, Cs) and lanthanide (La, Pr, Sm) oxides were additionally deposited on the surface of the cobalt FT catalysts at preset surface-specific loadings in the range of 0.1–4.0 M_at_ nm^–2^. The two series of promoters share a common Lewis basic character, which in the case of the alkali oxides stems from the strong e-donor character of their O^2–^ groups, while in the case of lanthanide oxides it is related to the electrodonating capacity of Ln^*x*+^ cations. Moreover, while those lanthanide elements studied as promoters share very similar ionic radii in the range of 96–103 pm, the ionic radii increases notably with the atomic number, from 102 pm for Na to 167 pm for Cs, for the series of alkali promoter elements.^42^ Bulk energy dispersive X-ray spectroscopy (EDS) analysis showed experimental promoter loadings in good agreement with the nominal contents, with relative deviations ≤20% (Table S2).

Co_3_O_4_ spinel was the only cobalt phase detected in all catalysts in their as-calcined state (Figure S2). Cobalt reducibility was studied with H_2_-temperature-programmed reduction (Figures S3 and S4). Two well-defined H_2_ consumption events were registered in the temperature range of 400–700 K, which are characteristic for the stepwise reduction of Co_3_O_4_ to metallic cobalt via CoO as intermediate.^43^ While these two reduction bands peaked at 450 and 655 K, respectively, for an unpromoted CoRu/AOmM catalyst, both reduction events shifted progressively to higher temperatures, by up to 77 and 33 K, respectively, on incorporation of a lanthanide oxide such as PrO_*x*_ at increasing contents up to 1.0 Pr_at_ nm^–2^, suggesting that interaction of the oxide promoter and the cobalt (oxide) species retards the reduction of the latter. Higher promoter loadings up to 3.0 Pr_at_ nm^–2^, however, led to a slight increase in reducibility as exemplified by a progressive down-shift of the CoO-to-Co^0^ reduction temperature (Figure S3), possibly as a result of a reduced interaction of cobalt species with the strongest acid sites on the Al_2_O_3_ carrier. A remarkably similar trend was observed when an alkali oxide such as NaO_*x*_ was applied as promoter (Figure S4). Interestingly, catalysts incorporating a reference surface content of 1.0 M_at_ nm^–2^ of different promoter oxides representative of the entire series studied herein (i.e., PrO_*x*_, NaO_*x*_, and CsO_*x*_) showed essentially identical H_2_-TPR traces, indicating that the surface atomic loading rather than the identity of the promoter determined cobalt reducibility, likely as a result of similar degrees of promoter-cobalt interaction in all cases. On the basis of the H_2_-TPR profiles, a reduction temperature of 673 K was selected to achieve essentially full cobalt reduction prior to catalysis.

**Figure 3:**
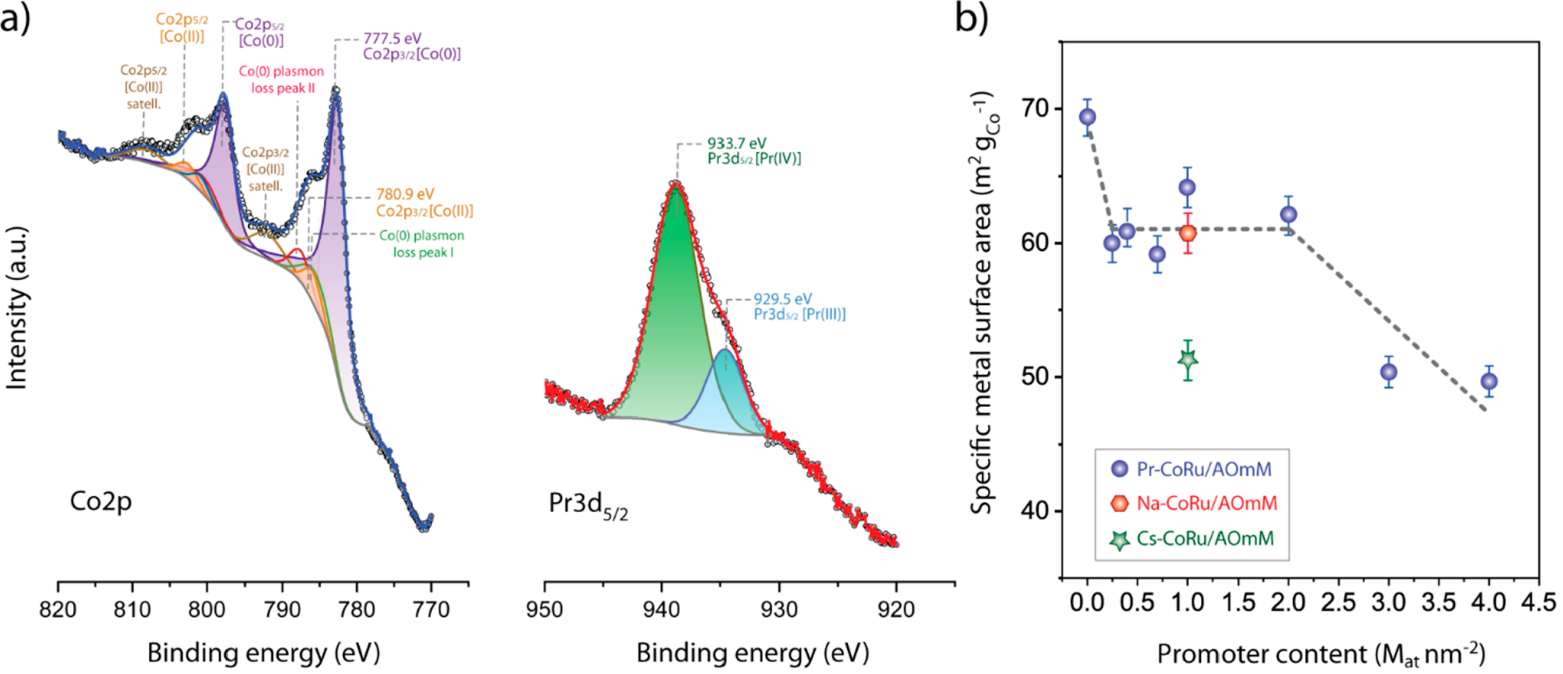
Surface characterization of PrO_*x*_-promoted Co-based FT catalysts. (a) X-ray photoelectron spectrum in the Co 2p and Pr 3d_5/2_ spectral regions for 1.0Pr-CoRu/AOmM after H_2_ reduction. (b) Cobalt-specific H_2_ chemisorption uptake for the series of Pr-CoRu/AOmM catalysts supported on multimodal meso-macroporous γ-Al_2_O_3_ as a function of the surface-specific praseodymium content. Error bars correspond to the standard error as determined from three independent chemisorption experiments on selected catalysts. The line is meant as a guide to the eye and it applies to the series of Pr-CoRu/AOmM catalysts.

For reasons which are going to be detailed later, further characterization studies focused on the series of catalysts incorporating PrO_*x*_ as promoter. Cobalt and promoter speciation were studied on selected catalysts after reduction activation by means of X-ray photoelectron spectroscopy (XPS). Figure 3a shows the XP spectra in the Co 2p and Pr 3d_5/2_ regions for 1.0Pr-CoRu/AOmM. Similar spectra were obtained for 3.0Pr-CoRu/AOmM, with a higher PrO_*x*_ loading (Figure S5). As expected from the H_2_-TPR profiles, metallic Co^0^ (BE Co 2p_3/2_ = 777.2–777.5 eV) is the major near-surface cobalt species after reduction (≥90%). The minor Co^2+^ contributions (BE Co 2p_3/2_ = 780.4–780.9 eV) are likely overrepresented after the *in situ* catalyst reduction preceding XPS experiments because of the poorer gas–solid hydrodynamics around pelletized samples and thus less efficient water convective evacuation, compared with packed-bed configurations applied for activation prior to catalysis. Further analysis of metal core–electron BEs for selected catalysts after *in situ* reduction and Fischer–Tropsch catalysis suggested that Ru, while intimately alloyed with Co after reductive activation, tends to segregate into Ru-rich aggregates under reaction conditions (see Table S3 and accompanying discussion). Moreover, the addition of praseodymium leads to a slight down-shift (by 0.2–0.3 eV), of the Co 2p BE for metallic cobalt, suggestive for a slight depletion in electronic charge on the near-surface Co^0^ atoms and therefore the electron-withdrawing character of the PrO_*x*_ species. The latter persists in oxidic form, predominantly as Pr^4+^, with ≤30% atomic contributions from Pr^3+^, both after catalyst reduction and following FT reaction conditions (Table S3).

**Figure 4:**
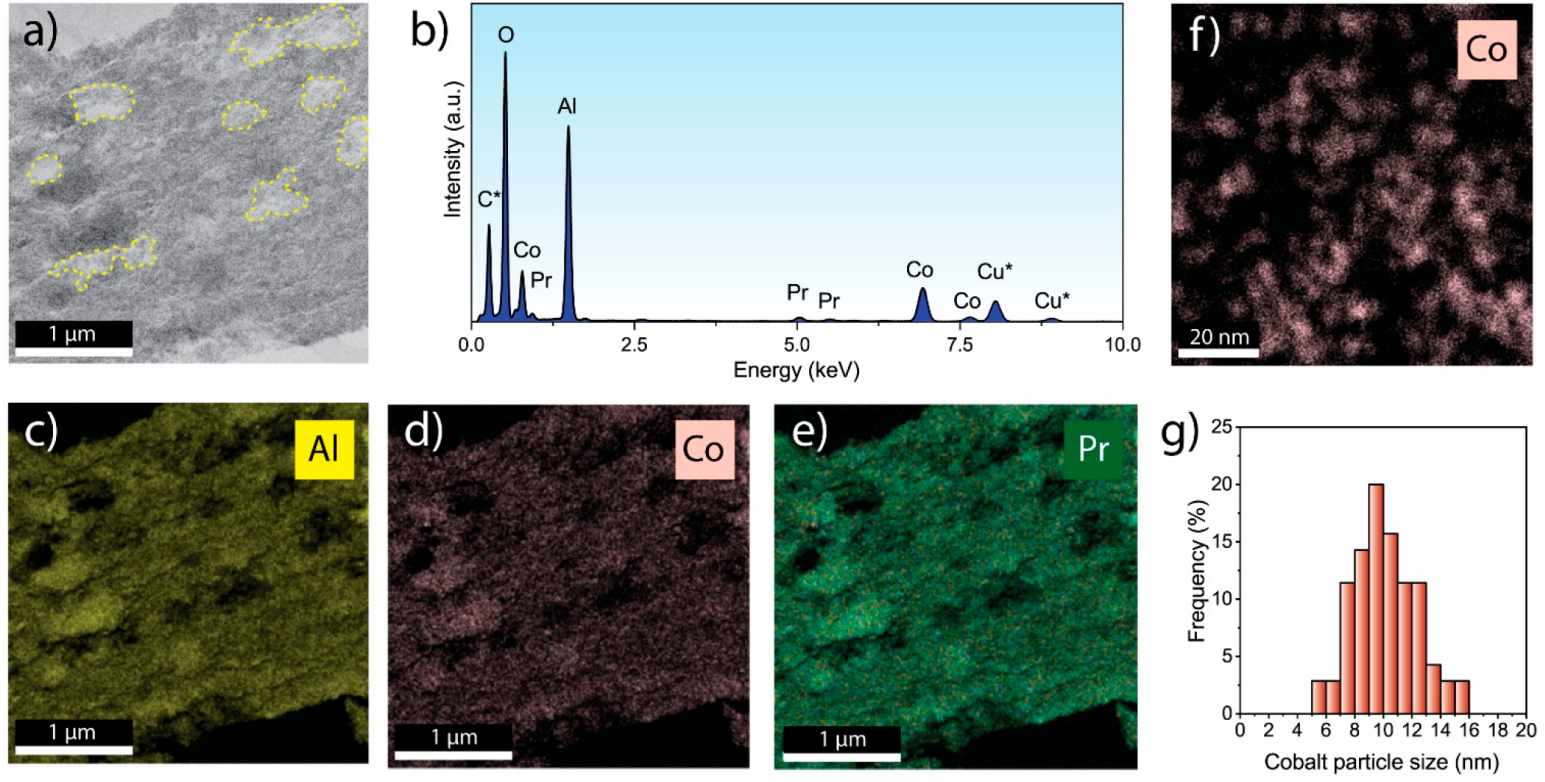
Bright field C_s_-STEM and EDS microanalysis on ultramicrotomed catalyst cross sections of 1.0Pr-CoRu/AOmM. (a) Representative BF-STEM mesoscale micrograph and; (b) the corresponding ED spectrum. Spectral contributions from the embedding carbonaecous resin and the copper grid are marked with asterisks. (c,d,e) EDS compositional maps for the region imaged in (a) for Al, Co, and Pr, respectively, obtained from the corresponding EDS K-lines. (f) Representative nanoscale EDS compositional map for Co. (g) Surface-averaged cobalt nanoparticle size distribution.

The dispersion and spatial distribution of cobalt and PrO_*x*_ on selected catalysts after H_2_ reduction was assessed by means of C_s_ aberration-corrected scanning-transmission electron microscopy (C_s_-STEM) and EDS nanospectroscopy on ultramicrotomed catalyst cross sections. Remarkably uniform spatial distributions for cobalt nanoparticles were ascertained at both the meso- and nanoscopic scales for an unpromoted CoRu/AOmM catalyst (Figure S6). The thermal decomposition of the nitrate catalyst precursors applied herein and developed previously by de Jong and co-workers^44^ effectively prevents the clustering of cobalt (oxide) nanocrystals, which therefore occupy the available support surface area with essentially maximum interparticle spacing. Figures 4 and S7 show representative micrographs and EDS elemental maps for the corresponding PrO_*x*_-promoted 1.0Pr-CoRu/AOmM. The excellent cobalt dispersion is preserved following the incorporation of the oxide promoter. Moreover, PrO_*x*_ appears as a nonparticulate phase, also evenly distributed on the catalyst surface at all analysis length scales. No preferential association of PrO_*x*_ species with cobalt was observed. Instead, praseodymium oxide appears highly dispersed and closely associated with the γ-Al_2_O_3_ carrier, likely due to a stronger interaction facilitated by the complementary mild basic and acidic surface character of these two oxides, respectively. Similarly, excellent dispersions and uniform spatial distributions for Co and PrO_*x*_ were also observed at higher Pr loadings (Figure S8).

Geometrical surface-averaged mean cobalt nanoparticle sizes, as determined from analysis of the STEM-EDX results, were similar (*d*_*s*_ = 10.2 ± 0.9 nm) for all catalysts studied, discarding any significant effect of PrO_*x*_ incorporation on cobalt dispersion. The exposed metal surface area was quantified with H_2_-chemisorption. Figure 3b shows its evolution with the Pr content for the series of Pr-CoRu/AOmM catalysts. A metal surface area of 69 m^2^ g_Co_^–1^ was determined for the unpromoted CoRu/AOmM, in fair agreement with the value of 61 m^2^ g_Co_^–1^ derived on geometrical considerations from STEM-EDS results. A ca. 12% drop in the exposed metal surface area is experienced upon incorporation of even minute amounts of the lanthanide oxide (0.4 Pr_at_ nm^–2^), suggesting the blockage of a fraction of the surface metal centers on the Co nanoparticles. Further increasing Pr loading up to 2.0 Pr_at_ nm^–2^ resulted in no statistically significant modifications of the metal surface area. Only upon further increasing the Pr content up to ≥3.0 Pr_at_ nm^–2^ was the available metal surface area further decreased, down to about 71% of that in the unpromoted catalyst. These results suggest that only a comparatively minor fraction of the metal centers become blocked by PrO_*x*_ species, already from comparatively low promoter loadings. The surplus praseodymium oxide interacts preferentially with the γ-Al_2_O_3_ surface and does not lead to further metal blockage for Pr loadings up to 2 Pr_at_ nm^–2^, beyond which point the promoter coverage on the metal increases more markedly with loading, likely as a result of decreased availability of binding centers on γ-Al_2_O_3_ for further PrO_*x*_ uptake. At an identical reference oxide promoter surface loading of 1.0 M_at_ nm^–2^, NaO_*x*_ led to a drop in the exposed Co surface of 12%, similar to that observed for PrO_*x*_, while for CsO_*x*_ this decrease reached 26%, suggesting that the capacity of the oxide promoters to block the metal surface scales with the cation size.

### Fischer–Tropsch Synthesis

The Fischer–Tropsch synthesis performance was evaluated in a fixed-bed reactor setup at industrially relevant conditions. The reaction temperature was set to 473 K, at the lower end of the technically significant temperature range for cobalt-based FT catalysis, in order to minimize secondary hydrogenation activity. Unpromoted CoRu/Al_2_O_3_ catalysts showed cobalt-specific reaction rates (cobalt-time-yield, CTY) in the range of 89–242 mmol CO g_Co_^–1^ h^–1^, which translated into similar *turnover frequencies* (TOF) per unit surface metal atom of 2.5 ± 1.0 × 10^–2^ s^−1^. At similar CO conversion levels of 20 ± 3%, CH_4_ selectivity spanned the range of 7.9–11.6%, while the selectivity to CO_2_ remained ≤0.6% in all cases, illustrating the inactivity of cobalt FT catalysts to the WGSR (Table 1, Table S4). ASF chain-growth probabilities (α) were comparatively similar in the range of 0.79–0.83, resulting in selectivities to condensable C_5+_ hydrocarbons of 74.3–80.8%. In spite of the comparable chain-length product distributions, the share of olefins in the C_5–10_ fraction depended conspicuously on the support pore diameter, increasing from 33.4% to 54.7% as the support pore size increased from 7 nm (monomodally mesoporous) to the more open pore architecture of the multimodal meso-macroporous AOmM. These results illustrate the direct dependence of the olefinicity for liquid product fractions on the effective (mesopore) transport distances and thus α-olefin pore residence times within the catalyst microparticles.

**Table 1:** Cobalt-Time-Yield (CTY), Product Selectivities, and Hydrocarbon Chain-Growth Probability (α) for Co-Based FT-Catalysts Supported on a Multimodal Meso-Macroporous γ-Al_2_O_3_ Support, and Optionally Modified with Various Alkali and Lanthanide Oxide Promoters^a^

catalyst	CTY^b^ [mmol CO g_Co^−1^_ h^−1^]	*S*(CO_2_) [C%]	*S*(CH_4_) [C%]	*S*(C_5+_) [C%]	*S*(C_5–10_ Olef.) [C%]	α^c^ [-]	C_2–4_ Olef.^d^ [%]	C_5–10_ Olef.^e^ [%]
CoRu/AOmM	88.9	0.6	7.9	79.2	13.3	0.80	56.5	54.7
1.0Na-CoRu/AOmM	32.9	1.0	8.0	76.0	14.4	0.77	54.3	56.2
3.0Na-CoRu/AOmM	8.5	8.1	10.8	53.2	10.1	0.66	36.1	53.1
1.0K-CoRu/AOmM	27.5	1.3	8.6	74.4	13.9	0.78	54.1	57.2
1.0Cs-CoRu/AOmM	10.9	2.9	11.4	65.0	12.1	0.71	56.7	54.6
1.0La-CoRu/AOmM	43.1	0.8	8.2	73.2	16.2	0.75	54.7	58.1
1.0Pr-CoRu/AOmM	36.7	0.9	8.4	70.2	19.9	0.75	57.5	61.5
3.0Pr-CoRu/AOmM	12.4	1.3	12.4	54.6	19.5	0.66	53.0	61.7
1.0Sm-CoRu/AOmM	47.3	1.1	9.2	73.6	14.6	0.76	54.8	53.2

a Reaction conditions: *T* = 473 K, *P* = 20 bar, H_2_/CO = 2, WHSV = 5.1–11.0 h^–1^, CO conversion = 20 ± 3%.

b Cobalt-time-yield.

c Chain-growth probability.

d Molar olefin abundance within the C_2–4_ hydrocarbon product fraction.

e Molar olefin abundance within the C_5–10_ hydrocarbon product fraction.

The addition of any of the Lewis basic promoters studied, whether alkali or lanthanide oxide, resulted in a decrease in CTY with respect to the unpromoted counterpart. This is illustrated in Table 1 for a set of CoRu/AOmM catalysts incorporating different promoter oxides at selected loadings of 1.0 and 3.0 M_at_ nm^–2^. These results tie in well with previous reports of a decreasing FT activity by incorporation of basic oxides of alkali and alkaline earth metals^45−47^ or lanthanum^48,49^ on Co-based FT catalysts. At a comparable loading of 1.0 M_at_ nm^–2^, the decrease in CTY was more pronounced (63–88%) and increased with increasing the cation size and decreasing electronegativity for the series of alkali promoters (Na < K < Cs), while it was less prominent and rather similar (47–59%) among the different lanthanide oxides studied.

Product selectivity was evaluated at an equivalent CO conversion level of 20%. All promoters led to a decrease in the chain-growth probability determined from the linearized ASF plots (Figure S9) and thus a decrease in selectivity to C_5+_ hydrocarbons. Both reductions^47^ and increments^50,51^ in C_5+_ selectivity have been reported previously for alkali-modified Co/γ-Al_2_O_3_ catalysts. Our results systematically point to a lower effective chain propagation upon modification with Lewis basic promoters, regardless of their identity. With regard to product olefinicity, while the effect was found to be limited for alkali oxides, lanthanide oxides such as La and most particularly Pr led to a significant increase in the olefin abundance within the C_5–10_ product fraction up to ca. 62%. Moreover, this enhancement in olefinicity for the lighter condensate products was achieved while retaining the essentially null WGSR activity which is intrinsic to metallic cobalt, with selectivities to CO_2_ ≤ 1.3%. In contrast, the addition of alkali oxides led to enhanced WGSR activities (S_CO2_ up to 8%) at comparable surface contents, in line with previous observations.^46,47,51^ Therefore, while alkali and alkaline earth (hydroxides) have been previously proposed as promoters to synthesize cobalt-based FT catalysts with enhanced selectivities to lower (C_2–4_) olefins at higher operation temperatures,^30,31,52^ it emerges from our results that lanthanide oxides, particularly PrO_*x*_, are preferred as promoters to target high selectivity to liquid (C_5+_) olefins at milder operation conditions.

**Figure 5:**
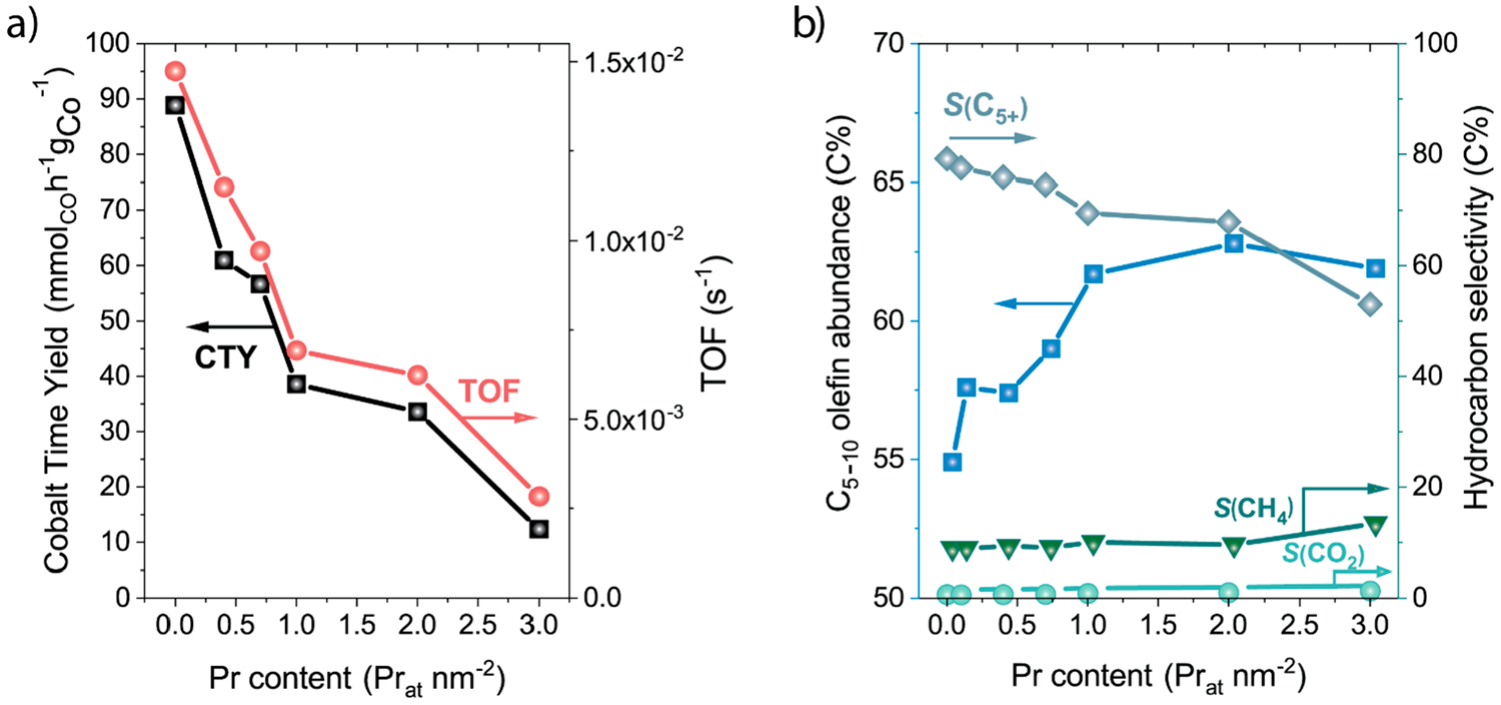
Impact of PrO_*x*_ promoter loading on catalyst performance. Evolution of (a) the cobalt-time-yield (metal-specific CO conversion rate) and the *turnover frequency* (TOF), per unit surface metal site as quantified with H_2_ chemisorption; and (b) the C_5_–C_10_ olefin abundance and hydrocarbon selectivities obtained with Pr-CoRu/AOmM catalysts supported on a multimodal meso-macroporous γ-Al_2_O_3_, as a function of the surface-specific praseodymium loading. Reaction conditions: *T* = 473 K, *P* = 20 bar, H_2_/CO = 2, WHSV = 8.7–18.0 h^–1^, CO conversion = 20 ± 3%.

Figure 5 summarizes the impact of the promoter surface loading on activity and selectivity for the series of *x*Pr-CoRu/AOmM catalysts. TOF decreased progressively with the Pr content, mirroring the behavior observed for CTY. This decrease in site-specific activity was pronounced already from low Pr contents and up to a Pr loading of 1.0 Pr_at_ nm^–2^, indicating that comparably low promoter surface coverages on cobalt are sufficient to notably reduce the intrinsic CO hydrogenation activity. Regarding product selectivity, praseodymium contents up to 1.0 Pr_at_ nm^–2^ had only marginal effects on the selectivity to CH_4_ and CO_2_, which remained within 8.0–8.4% and 0.6–0.9%, respectively. The overall selectivity to C_5+_ condensates did show a measurable and progressive decrease from 79.2% to 70.2% with increasing Pr content in the same range, as a result of a decrease in α from 0.80 to 0.75. More remarkably, the olefin abundance within the C_5–10_ fraction increased systematically to exceed 61% at a promoter loading of 1.0 Pr_at_ nm^–2^. Further increments in Pr loading up to 3 Pr_at_ nm^–2^ led to a more pronounced decrease in S_C5+_, with CH_4_ selectivity reaching up to 12.4%, while the selectivity to olefins within the C_5–10_ slate essentially plateaued off. Hence, a Pr loading of 1.0 Pr_at_ nm^–2^ was found to maximize the selectivity to liquid olefins while preserving a low methanation activity and CO_2_ side-production.

Moreover, open porosity and PrO_*x*_ promotion both enhanced the selectivity to linear alcohols, which improved from 5.0 C% for the mesoporous CoRu/AOm(7) to 8.3 C% for the multimodally porous CoRu/AOmM and reached 11.7 C% for 1.0Pr-CoRu/AOmM, for which an alcohol chain growth probability of α_OH_ = 0.73 was determined (Figure S10). For the latter catalyst, this added up to an overall 67% carbon abundance of chemicals (1-alcohols and olefins) in the C_5–10_ product fraction. The formation of *n*-alcohols on cobalt-based catalysts under syngas conversion conditions has been previously associated with the presence of Co_2_C/Co^0^ interfaces on the working catalysts.^53^ After prolonged exposure to FT reaction conditions, no signs for Co_2_C crystallites could be detected by XRD for neither the unpromoted CoRu/AOmM nor for 1.0Pr-CoRu/AOmM (Figure S11). However, contact with the PrO_*x*_ promoter might create small CoC_*x*_ surface domains which, undetectable by XRD, promote alcohol synthesis. Jointly, these results illustrate that, PrO_*x*_-promotion, albeit at the expense of the overall CO conversion rate, led to a FT product distribution which is desirable for a combined production of paraffinic middle-distillates and waxes (C_11+_) and higher value C_5–10_ chemicals, essentially without CO_2_ side-production, from H_2_-rich syngas.

**Figure 6:**
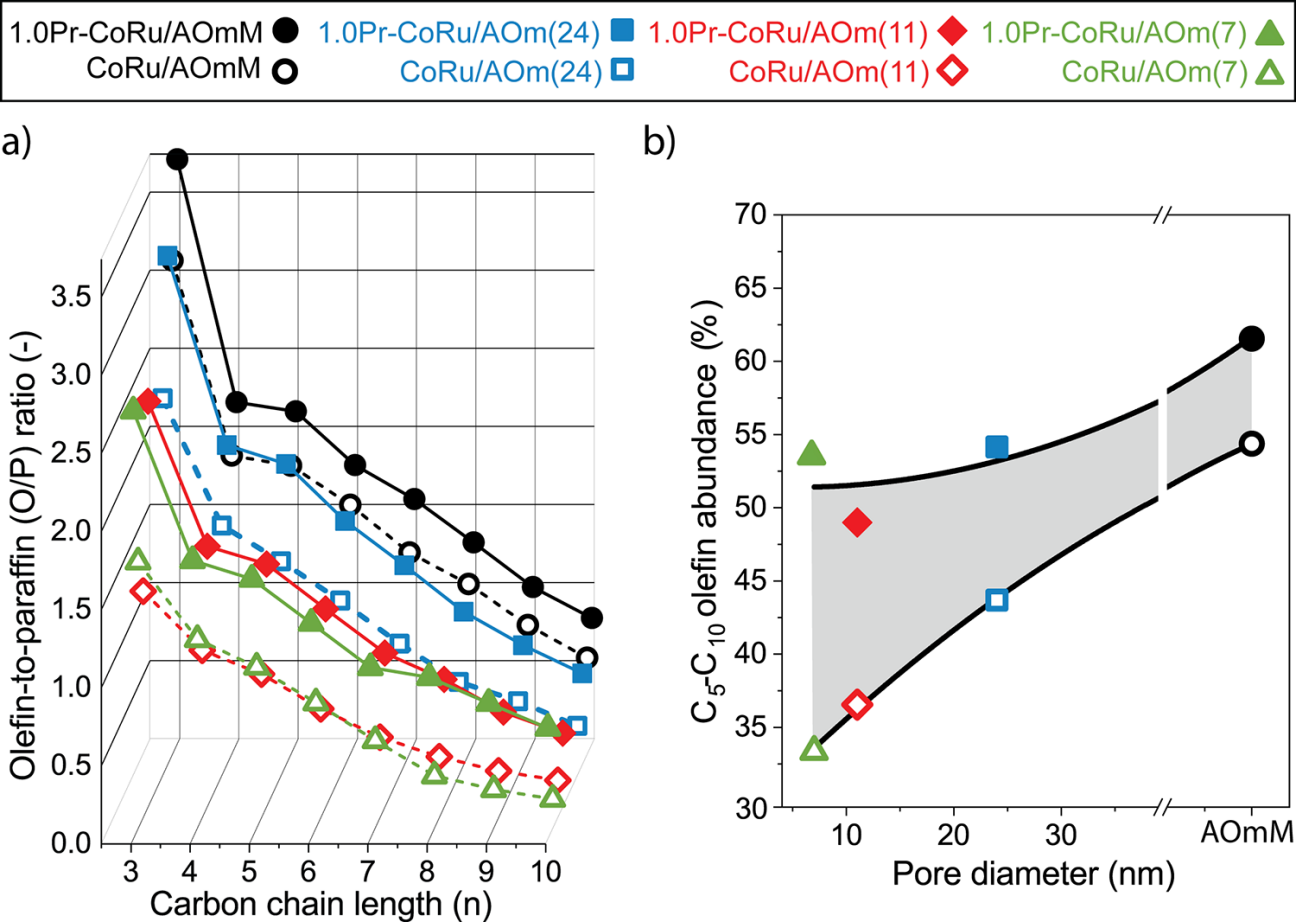
Combined effects of support porosity and chemical promotion on the selectivity to liquid olefins. Evolution of (a) the olefin-to-paraffin molar ratio with the carbon chain-length for C_3–10_ hydrocarbon products and; (b) the olefin molar abundance within the C_5–10_ liquid products with the average support pore diameter, obtained with various cobalt-based FT catalysts supported on unimodal mesoporous or multimodal meso-macroporous γ-Al_2_O_3_ carriers of varying pore diameters either in their unpromoted form (open symbols and dotted lines) or promoted with PrO_*x*_ (1.0 Pr_at_ nm^2^) (closed symbols and solid lines). In panel (b), catalysts to the right of the *x*-axis interrupt show a multimodal pore architecture with both meso- and macropores and thus no specific average pore diameter has been assigned. Reaction conditions: *T* = 473 K, *P* = 20 bar, H_2_/CO = 2, WHSV = 5.5–33.0 h^–1^, CO conversion = 20 ± 3%. The gray-shaded area on panel (b) illustrates the increase in olefinicity for the C_5–10_ liquid FT products due to the incorporation of PrO_*x*_ as promoter.

The systematic set of catalysts addressed in this study enabled disentangling the contributions of pore architecture and oxide promotion to this unusual product distribution. Neither the multimodal meso-macroporous structure nor the addition of PrO_*x*_ as surface promoter modified stability (∼200 h on-stream) under Fischer–Tropsch reaction conditions (Figure S12). Figure 6a shows the evolution of the olefin-to-paraffin (O/P) molar ratio in the range of C_3–10_ as a function of the hydrocarbon chain length for both unpromoted and PrO_*x*_-modified (1.0 Pr_at_ nm^–2^) catalysts supported on γ-Al_2_O_3_ carriers of varying pore diameter. For every catalyst, the O/P-ratio decreases with the hydrocarbon chain length, an effect which is ascribed to the chain-length dependent average pore residence time of α-olefin primary products, thus the extent to which they undergo secondary hydrogenation, under reaction conditions.^9^ In all cases, the addition of PrO_*x*_ as promoter led to a substantial increase in olefinicity. As observed in Figure 6b, the magnitude of this effect scales systematically with the support porosity, being more marked for narrow-pore catalysts and decaying progressively in magnitude as the catalyst pore architecture becomes more open. Therefore, while the role of PrO_*x*_ is likely the same in all cases, that is, reducing the hydrogenation capability of surface metal sites responsible for secondary olefin hydrogenation, the magnitude of the promotion depends as well on the effective (meso)pore transport lengths for α-olefin primary products. In the study space addressed, the combined effect of porosity design and PrO_*x*_ promotion leads to a ca. 2-fold raise in olefin selectivity within the liquid C_5–10_ products.

The hydrogenation of olefins on metal nanoparticles is considered to be a structure insensitive reaction under H_2_ atmospheres, and hence, activity scales linearly with the available metal surface area.^54,55^ Compared with standard hydrogenation conditions, under Fischer–Tropsch reaction settings, molecular CO is known to be the dominant adsorbate on metallic cobalt (and ruthenium) FT catalysts, leaving fewer available vacant sites for olefin adsorption and H_2_ dissociation.^56^ For the series of catalysts studied here, no correlation was found between the cobalt surface area and the O/P (Figure S13), suggesting that simply a reduction in the exposed metal surface does not explain, on its own, the lower extent of olefin hydrogenation. It thus stands to reason to infer that PrO_*x*_ brings about a preferential dampening of the cobalt hydrogenation reactivity.

In addition to hydrogenation, α-olefin primary FT products might undergo other secondary reactions, which include not only reinsertion into growing hydrocarbon chains but also double-bond isomerization to linear 2-alkenes, which may be catalyzed by metallic sites,^8^ as well as acidic or basic centers of the oxide catalyst support.^57,58^ A number of interesting downstream upgrading routes for liquid FT olefins (e.g., hydrosylilation, hydroformylation, etc.) benefit from high selectivity to end-of-chain functionalization.^59−62^ Therefore, the terminal-to-internal (T/I) ratio in the C_5–10_ olefin products is of interest as a figure-of-merit for FT catalysts designed to achieve high selectivity to liquid olefins. Under FT reaction conditions, the T/I olefin ratio decreased progressively with increasing the hydrocarbon chain length in all cases (Figure S14), reflecting that olefin isomerization secondary reactions are also transport-enhanced. As a result, in the absence of oxide promoters, the α-olefin abundance in the C_5–10_ olefin products increased with increasing the support pore size, from 79.5% for CoRu/AOm(7) to 92.2% in the case of the hierarchically porous CoRu/AOmM. On top of these porosity effects, the addition of PrO_*x*_ as promoter on the surface of the meso-macroporous CoRu/AOmM catalysts led to a further and significant enhancement in the terminal olefin abundance, already from very low Pr loadings of 0.1 Pr_at_ nm^–2^. For Pr contents ≥1.0 Pr_at_ nm^–2^, abundances of α-olefins in excess of 95% in the C_5–10_ fraction were achieved. Essentially all olefin products in this product fraction were found to be linear, as no branched olefin isomers could be detected (Figure EM2b in the Supporting Information). These high structural and regio-isomery selectivities are desired for downstream olefin functionalization into linear specialty chemicals (e.g., alcohols via olefin (reductive) hydroformylation with syngas).

Additional insights into the promotional effects on secondary olefin double-bond shift reactions were gathered from the analysis of the steroisomery of 2-butene products. Similarly to the T/I ratio, the impact of the PrO_*x*_ promoter on the 2-butene stereoselectivity was apparent already from very low Pr loadings (Figure S15). The *cis*/*trans* 2-butene ratio in the products increased steeply from ca. 1.4 for the unpromoted CoRu/AOmM to >1.9 for 0.1Pr-CoRu/AOmM. Further, less pronounced increments were observed on progressively increasing Pr content, leveling off at ca. 2.2 for 3.0 Pr_at_ nm^–2^. Experimental *cis*/*trans* 2-butene ratios are in all cases higher than the value of 0.53 predicted under equilibrium conditions at the reaction temperature of 473 K^63^ and are therefore the result of kinetic control. The increment observed in both the abundance of terminal olefins as well as in the share of *cis* isomers within 2-olefins on promotion with PrO_*x*_ suggests the inhibition of acid-catalyzed secondary olefin double-bond isomerization, which proceeds via carbocation intermediates and is thus expected to favor *trans* products. On the contrary, Lewis basic sites are less efficient isomerization centers and are expected to promote carbanion routes via H-abstraction, which favor *cis* isomers.^57^

Also with regard to the preservation of terminal isomers within the C_5–10_ olefin products, PrO_*x*_ was the most effective promoter among those investigated. Albeit less marked, increments in the C_5–10_ olefin T/I ratio were also observed on modification of CoRu/AOmM with alternative promoters, including oxides of alkali metals and alternative lanthanides such as Sm (Figure S16). With LaO_*x*_, in contrast, even though its addition enhanced the olefinicity of this hydrocarbon product fraction, it decreased the T/I olefin ratio in the entire chain-length range compared with the unpromoted benchmark. This effect is tentatively ascribed to the previously reported and rather exclusive ability of lanthanum—within the early lanthanide elements—to facilitate the development of stronger acid sites, that is, being more reactive for olefin isomerization via carbocation routes, when deposited on the surface of γ-Al_2_O_3_.^64^

### In Situ FTIR Spectroscopy

#### PrO_*x*_ Effects on Metal Surface Sites

**Figure 7:**
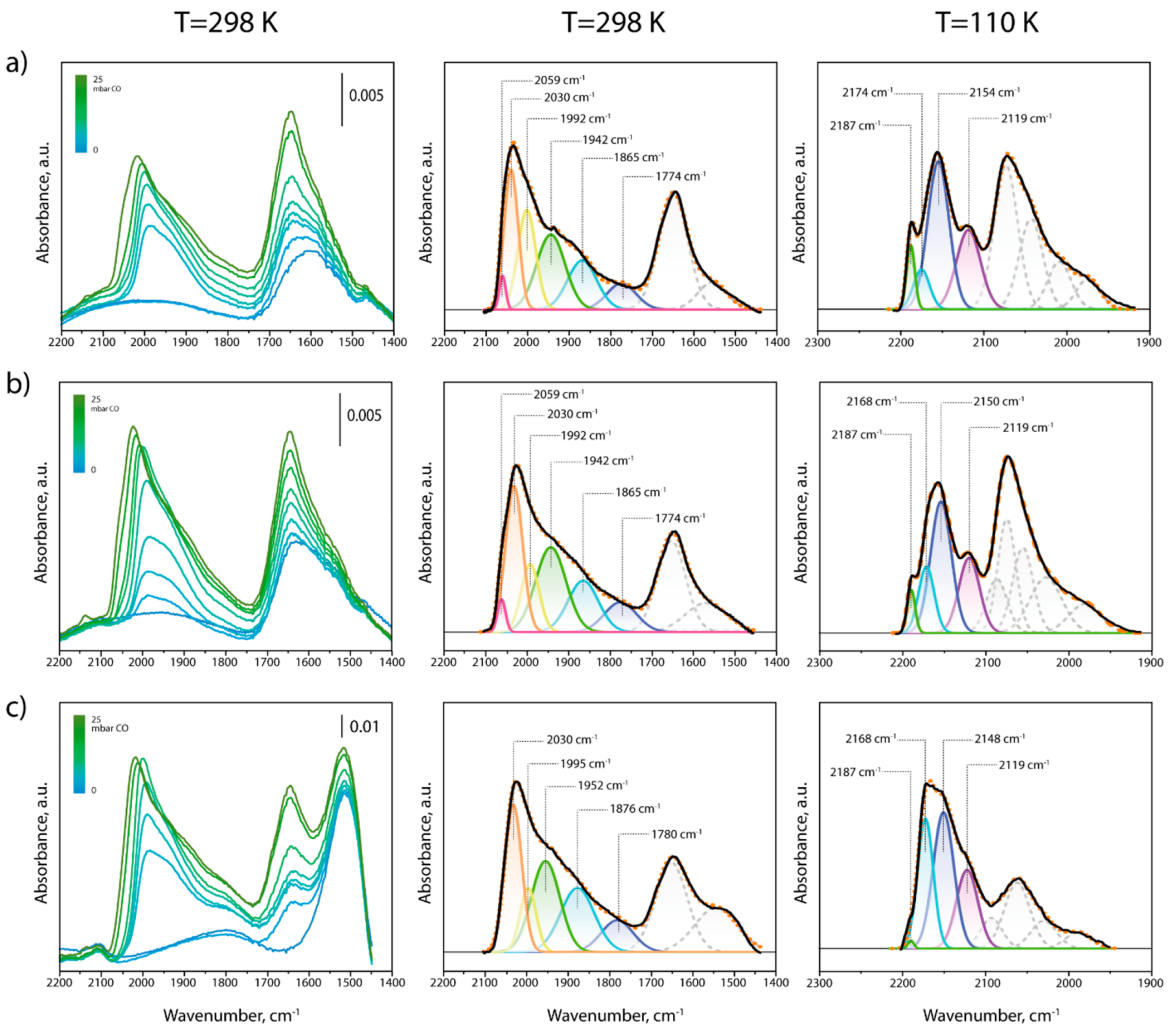
*In situ* CO-FTIR investigation on promoter effects. FTIR spectra recorded at 298 K in the υ(CO) region after increasing CO doses in the range *P*_CO_ = 0–25 mbar (left); deconvolution of the FTIR spectrum recorded at 298 K after having dosed ca. 25 mbar CO (center); and deconvolution of the FTIR spectrum recorded at 110 K after having dosed 2 mbar CO (right, see Figure S20 in the Supporting Information for the full set of spectra at increasing *p*_CO_ = 0–2 mbar), on the *in situ* reduced (a) CoRu/AOmM, (b) 1.0Pr-CoRu/AOmM, and (c) 3.0Pr-CoRu/AOmM catalysts.

Following *in situ* catalyst reduction, CO-FTIR spectroscopy was applied at room temperature to investigate the metal surface topology in selected catalysts, that is, unpromoted CoRu/AOmM, and the corresponding catalysts additionally incorporating 1.0 and 3.0 Pr_at_ nm^–2^, respectively. As shown in Figure 7, bands in the spectral region 1800–2100 cm^–1^, ascribed to υ(CO) in surface cobalt carbonyls, developed on increasing the CO dose. Additionally, bands in the region 1500–1800 cm^–1^ indicated the presence of carboxyl and carbonyl compounds on the oxide support after H_2_-reduction activation. Spectra deconvolution was applied to discriminate different contributions. Figure 7 shows the corresponding deconvolutions at *P*_CO_ ∼ 25 mbar, that is, prior to the detection of significant coverage-driven band shifts (see Figure S17 for further deconvolution details at various CO dosages). Similar contributions were observed in all cases, suggesting that the addition of PrO_*x*_ did not modify the metal surface topology to a great extent. Comparatively broader bands peaking at ca. 1870 ± 6 cm^–1^ and 1947 ± 5 cm^–1^ can be ascribed to CO adsorbed in 3-fold and bridged configurations, respectively, on cobalt.^65,66^ Moreover, already from the lowest CO dosages, a weaker and narrower band emerged at ca. 1992–2000 cm^–1^. This is the spectral region where atop cobalt carbonyls have been detected on low-coordination surface metal centers on stepped and defect-rich sputtered cobalt monocrystals,^66^ and hence, the band might be associated with CO adsorbed at surface defects (e.g., step-edges on the cobalt nanoparticles). This band progressively decreased in relative intensity and clearly shifted to lower wavenumbers on incorporating PrO_*x*_, suggesting that oxide promoter species might interact with lower coordination surface cobalt atoms, blocking a fraction of these sites for CO binding, while enhancing the electron backdonation of the remaining centers to CO*. At higher CO doses, the most prominent band at ca. 2030 cm^–1^, corresponding to atop CO adsorption on cobalt facets,^66,67^ dominated this spectral region. Finally, an additional evident implication of the incorporation of PrO_*x*_ as promoter was observed at higher wavenumbers. For the unpromoted CoRu/AOmM, a band centered at 2059 cm^–1^ became evident at *P*_CO_ > 10–15 mbar, clearly as a new contribution rather than as a result of blueshifts of bands developed at lower CO doses. This υ(CO) frequency is typically assigned to CO linearly adsorbed on cobalt terraces reconstructed by C*, originated from CO dissociation, which might already occur at room temperature.^56,66,68^ While this contribution was also observed for 1.0Pr-CoRu/AOmM, a higher Pr content of 3 Pr_at_ nm^2^ evidently led to its disappearance (see also Figure S18a for a CO dosage-resolved analysis), suggesting that at high oxide surface loadings, PrO_*x*_ species have also modified the reactivity of extended terraces on the cobalt nanoparticles. At yet higher CO doses (*P*_CO_ > 25 mbar), atop metal carbonyl bands underwent a progressive blueshift, as a result of the establishment of dipole–dipole interactions among vicinal CO* adsorbates at high surface coverages. Moreover, several comparatively narrow absorption bands emerged in the region of 1850–2000 cm^–1^, indicating the generation of molecular cobalt (sub)carbonyl compounds and their readsorption on the catalyst surface (Figure S19).^69^ Interestingly, this phenomenon became progressively exacerbated on incorporation of increasingly higher PrO_*x*_ contents. This observation suggests a progressively higher fraction of surface metal atoms to be depleted in electronic density and therefore more prone to react to the electron donor CO into molecular adducts, at the Co/PrO_*x*_ contact points.

#### PrO_*x*_ Effects on Surface Acid Sites

Next to modifications on the metallic function, the impact of the PrO_*x*_ species on the catalyst support surface was also assessed by CO-FTIR at cryogenic temperatures (110 K). At this temperature, CO probes not only metallic sites, but additionally, it serves as a sensitive reporter for surface Lewis centers. Upon admission of CO in the IR cell, following catalyst reduction, surface metal carbonyl bands (1900–2100 cm^–1^) developed already from the lowest CO doses, as a result of the stronger CO binding to metallic sites, while signals in the range of 2100–2200 cm^–1^, associated with CO adsorbed on the oxide supports, emerged on further increasing *P*_CO_ up to 2 mbar (Figure S20). Figure 7 shows deconvolution results for spectra recorded at the highest CO surface coverage for the unpromoted CoRu/AOmM as well as those counterparts incorporating 1.0 and 3.0 Pr_at_ nm^–2^, respectively (see Figure S21 for deconvolution details at various CO dosages). The corresponding cobalt-free Al_2_O_3_ and 3.0Pr/AOmM materials were also studied separately to support band assignment (Figure S22). For all catalysts studied, a contribution peaking at 2119 cm^–1^ can be ascribed to atop CO adsorption on partially oxidized Co^δ+^ sites.^70^ Given the high overall degree of cobalt reduction ascertained by XPS, these partially oxidized centers are likely located at the metal–support interface. A prominent band centered at ca. 2150 ± 4 cm^–1^ is associated with CO adsorbed via H-bond interactions with surface OH groups on the alumina surface.^71−73^ Contributions peaking at 2174 and 2187 cm^–1^ for the unpromoted CoRu/AOmM catalyst can be ascribed to CO bonded to coordinatively unsaturated (*cus*) Al^3+^ Lewis centers of medium and higher acid strength, respectively.^71,72^ On incorporation of PrO_*x*_ at increasingly higher surface loadings, the band peaking at ca. 2187–2190 cm^–1^, associated with the strongest Al_2_O_3_ Lewis sites, vanished progressively (see also Figure S18b for a CO dosage-resolved analysis), while a band developed at 2168 cm^–1^. The latter, also observed on the metal-free PrO_*x*_/AOmM, can thus be assigned to a new type of surface Lewis acid sites associated with *cus* praseodymium cations. The comparatively lower υ(CO) indicates that this new type of sites bears lower acid strength (electron-withdrawing character) compared with those on the pristine Al_2_O_3_. While such acidity inhibition was evident already at promoter loadings of 1.0 M_at_ nm^–2^, CO_2_-TPD studies showed that the development of strong surface basic centers was only noticeable at higher promoter contents of 3.0 M_at_ nm^–2^ (Figure S23). These results furnish direct evidence that PrO_*x*_ species are also responsible for the depletion of the strongest Lewis acid sites on the alumina oxide support, in favor of weaker acid centers and basic sites associated with the lanthanide oxide, explaining its decisive role in inhibiting the acid-catalyzed olefin double bond isomerization and thus enhancing the terminal regioselectivity as well as *cis* stereoselectivity of liquid olefin products.

### DFT Insights into Promotional Effects

#### Preferential Promoter Location on Cobalt

Density functional theory calculations were performed to further rationalize those metal-oxide promotional effects observed experimentally. Na_2_O and PrO_2_ oxides were selected as representative for the experimentally investigated alkali and lanthanide oxide promoters, respectively. First, the structure and adsorption energetics of the promoters on cobalt were assessed. Face-centered-cubic (*fcc*) Co^0^ was the predominant polymorph in all catalysts, both unpromoted and oxide-promoted, after activation via reduction in hydrogen flow (Figure S24). Hence, few-atom-layer thick slabs for Co(111) and Co(211) metal facets were considered as representative models for flat and stepped cobalt surfaces, respectively, in order to contemplate both extended terraces as well as step-edge topological motifs present on the surface of *fcc* cobalt nanoparticles (Figure CM1 and CM2).

**Figure 8:**
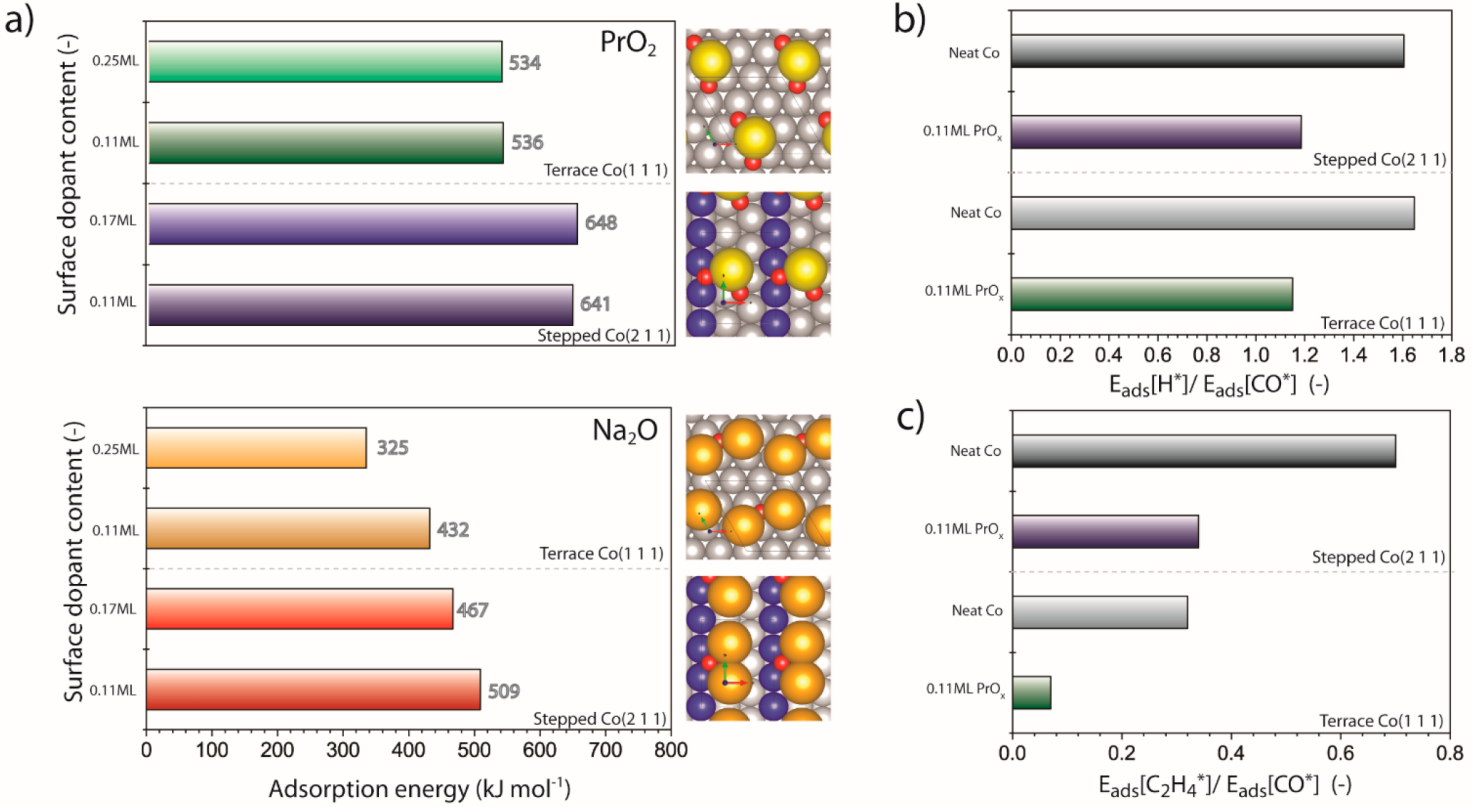
DFT calculations on promoter effects. (a) Adsorption energies for PrO_2_ and Na_2_O on flat Co(111) and stepped Co(211) cobalt surfaces as a function of the oxide coverage (fractional *monolayer* (ML)). The top views on the models show the corresponding optimized structures for a 0.11 ML oxide coverage in each case. Color codes: Pr: yellow, Na: orange, O: red, Co: gray and blue. For clarity cobalt step-edges on Co(211) are shown in blue. (b) Relative adsorption energies for H* and CO* on Co(111) and Co(211) cobalt surfaces either as neat or precovered with 0.11 ML PrO_2_. (c) Relative adsorption energies for ethylene (C_2_H_4_*) and CO* on Co(111) and Co(211) cobalt surfaces either as neat or precovered with 0.11 ML PrO_2_.

Figure 8a summarizes the results for the adsorption energetics of the oxide promoter species on the different cobalt surfaces at various surface coverages. Further details on the optimized structural models as well as the atop, bridge and hollow (*fcc* and *hcp*) surface adsorption sites considered are provided in the Supporting Information (section 2, Computational Methods). Higher *E*_ads_ indicate that PrO_2_ monomers bind stronger to cobalt surfaces, regardless of surface topology, compared with Na_2_O counterparts. At a comparable 0.11 ML promoter surface coverage on Co(111) terraces, the most stable adsorption configuration for Na_2_O was found with sodium occupying cobalt *fcc* hollow and bridge sites and oxygen anions on *fcc* sites, while for PrO_2_, oxygen ions occupied *hcp* and *fcc* hollow sites, and Pr a *hcp* hollow metal site. On the stepped Co(211) surfaces, the preferred adsorption structure for Na_2_O is with Na atoms located at the cobalt *hcp* sites on the lower terrace and only the oxygen ion siting at the step-edge, while PrO_2_ adopts an adsorption structure with oxygen anions on *hcp* hollow sites on the step and lower terrace, respectively. Details on the predicted structures at higher promoter coverages are provided in the Supporting Information. As shown in Figure 8a, the adsorption of both oxide monomer species is found to be energetically more favored on Co(211). In line with the experimental CO-FTIR results, these predictions bolster the preferential population of step-edge sites on the metal nanoparticles in the FT catalysts with promoter species at low oxide coverages.

#### Promoter Effects on Cobalt

Next, the impact of promoters on the adsorption energetics for H* and CO* species was investigated. Calculations focused on a promoter coverage of 0.11 ML, given that higher oxide surface contents, that is, of 0.25 ML on Co(111) and 0.17 ML on Co(211), resulted in either CO desorption to the gas phase or decomposition of the promoter structures, suggesting that comparatively lower oxide coverages are attainable experimentally on cobalt. Figure 8b summarizes the results for PrO_2_. As observed, the presence of PrO_2_ species on cobalt leads to a decrease in the ratio *E*_ads_[H*]/*E*_ads_[CO*] compared with the bare metal surface, primarily as a result of a notable increment in the binding energy for molecular CO on the oxide-decorated cobalt surface (Tables S5 and S6). This effect is slightly more pronounced on Co(111) terraces, whereon several energetically feasible adsorption sites could be identified for hydrogen and CO, with different spatial proximity to oxygen anions or lanthanide cations (section 2 in the Supporting Information), with H* and CO* adsorption energies spanning 276–262 kJ mol^–1^ and 240–226 kJ mol^–1^, respectively. Qualitatively similar results were obtained for the case of Na_2_O (Tables S5 and S6).

A Bader charge analysis indicates that, on bare cobalt surfaces, both CO* and H* adsorbates withdraw electron density from the surface cobalt atoms, in line with previous experimental findings,^74,75^ and regardless of the metal surface topology considered (Table S7). Addition of either Na_2_O or PrO_2_ oxide promoters results in additional electronic charge accumulation on H* and CO*. On the one hand, this suggests an enhanced electron backdonation from cobalt atoms in the vicinity of the oxide promoter species to CO*, which is in line with those red shifts observed using FTIR for υ(CO) in atop cobalt carbonyls at low-coordination metal sites in PrO_*x*_-promoted catalysts (*vide supra*). On the other hand, it contributes to a slight reduction of *E*_ads_[H*]^32^ alongside a marked increase of *E*_ads_[CO*]. Even though the actual surface coverage remains a matter of debate,^56^ it is known that under relevant FT reaction conditions, the surface of cobalt catalysts is by and large occupied by molecular CO. Surface CO* coverages in the range from ca. 0.6 ML to essentially a monolayer have been proposed under operation conditions.^57−59^ Therefore, the increase in *E*_ads_[CO*] relative to *E*_ads_[H*] is unlikely to result in major further increments in the CO* coverage on the metal. However, comparatively minor CO* coverage increments have been proposed to be sufficient to hinder the dissociative H_2_ adsorption by decreasing the availability of those surface vacant sites involved in hydrogen activation.^57^ This effect can explain the lower hydrogenation activity observed on the promoted cobalt catalysts, which results in both lower overall CO hydrogenation reaction rates as well as higher selectivity to olefins.

Next to the primary hydrogenation ability, further calculations were performed to gain insight into secondary effects induced by the oxide promoters. The extent to which α-olefin primary reaction products undergo secondary hydrogenation is a function of their bed and pore residence times—adjustable via catalyst porosity—but also the driving force for olefin adsorption on the metal. The latter was explored using ethylene as a model olefin. As shown in Figure 8c, a 0.11 ML coverage of PrO_2_ on cobalt is predicted to lead to a remarkable decrease in *E*_ads_[C_2_H_4_*]/*E*_ads_[CO*] compared with neat cobalt, by 71% and 36% for flat Co(111) and stepped Co(211) surfaces, respectively. This lower driving force for olefin readsorption might additionally contribute to the dampening of secondary olefin hydrogenation observed experimentally on PrO_*x*_-promoted catalysts.

A second important secondary effect of oxide promoters relates to their capacity to prompt the WGSR on the essentially shift-inactive cobalt FT catalysts. This effect is generally ascribed to the development of (surface) cobalt carbide species under reaction conditions.^76^ In this regard, Bader charge analyses for the surface metal atoms revealed significant differences in the electronic effects induced by Na_2_O and PrO_2_ oxides on cobalt (Table S7). While Na_2_O acts as strongly electron-donating species, PrO_2_ units behave overall as electron-withdrawing groups, a behavior which dovetails with the different origin of the basic character of the two oxides. This is particularly apparent on the stepped Co(211) surface, which is the energetically most favorable location of both oxide promoters at low surface coverages. Even in the presence of electron acceptor CO* adsorbate molecules, that is, a situation more representative of the working catalyst, surface decoration with Na_2_O leads to a decrease in the net Bader charge on cobalt, from 0.63 to 0.50 with respect to the unpromoted metal surface (Table S7). In contrast, the presence of PrO_2_ results in notably more electropositive cobalt atoms, with a net Bader charge of 1.50. These markedly different behaviors have implications for the carbophilicity, which might serve as an indication for the propensity of cobalt surfaces to undergo carbidization. Adsorption energies for C* were evaluated (see section 2 in the Supporting Information). The adsorption energy for C* on clean Co(111) and Co(211) surfaces is determined to be 691 and 705 kJ mol^–1^, respectively. While *E*_ads_[C*] increases, to 713 and 714 kJ mol^–1^, respectively, on decoration with PrO_2_, a higher increment, to 714 and 721 kJ mol^–1^, was found after deposition of Na_2_O, making the cobalt surface more carbophilic. Reinforcing these computational predictions and in line with previous reports of a facilitated carbide formation upon incorporation of alkali promoters,^77,78^ XRD analysis of catalysts recovered after the FT tests revealed the development of Co_2_C crystalline domains in 3.0Na-CoRu/AOmM, that is, the catalyst showing the most marked increase in CO_2_ selectivity (Figure S11). These results allow thus to surmise that the enhanced carbon affinity and thus a facilitated development of surface carbide species during catalysis underlie the undesired boosting of the WGSR activity observed experimentally for NaO_*x*_ and other alkali oxides.

## Conclusion

A battery of cobalt-based Fischer–Tropsch catalysts synthesized on either monomodal mesoporous or multimodal meso-macroporous alumina support materials enables untwining and assessing pore transport and oxide surface promotion effects on the selectivity to liquid (C_5+_) olefin chemicals. Under relevant reaction conditions, reducing mesopore transport distances for primary products down to the (sub)micrometer regime, in hierarchically porous meso-macroporous catalysts, contributes to a higher preservation of olefins in the condensable hydrocarbon products. The incorporation of promoters of Lewis basic character (alkali and lanthanide oxides) leads in all cases to a decrease in the metal surface specific CO hydrogenation rate (TOF) and hydrocarbon chain-growth probability. Lanthanide oxides, particularly praseodymium oxide, proved efficient as promoters to further boost the share of α-olefins within the light condensate (C_5–10_) products, notably, while preserving key features of cobalt-based catalysts such as low activity to the WGSR which was, on the contrary, exacerbated by alkali oxide promoters at similar surface contents. DFT calculations predict oxide promoters on cobalt surfaces to locate preferentially at surface metal step-edges and associate their promotion effect to an enhanced adsorption competition of CO for surface metal sites compared to hydrogen and olefins, thereby dampening (secondary) hydrogenation. Moreover, our results suggest an enhanced metal surface carbophility because of the strong electron donor character of alkali oxides compared to lanthanide oxides as the likely reason for the undesired triggering of WGSR activity by the former. Next to surface promotion on the metal nanoparticles, *in situ* CO-FTIR spectroscopy furnishes evidence for the blockage of the strongest Al^3+^*cus* Lewis acid centers on the γ-Al_2_O_3_ support upon PrO_*x*_ incorporation, explaining a second promotional effect which enhances the terminal regioselectivity in the liquid olefin products via inhibition of acid-catalyzed double-bond shift secondary reactions. Integrating a multimodal meso-macroporous architecture with PrO_*x*_ as promoter, at an optimal loading of 1 Pr_at_ nm^–2^, leads to an unconventional product distribution which reconciles features of cobalt and iron carbide-based FT catalysts, respectively. Selectivities to C_5+_ products >70 C% are achieved in combination to light condensate products (C_5–10_) enriched in added-value chemicals (67 C%), predominantly α-olefins, remarkably with essentially no CO_2_ side-production (<1%). These results illustrate how porosity design and surface promotion effects are valuable and complementary tools to adjust the FT product distribution toward the combined production of paraffinic precursors for synthetic fuels and liquid commodity chemicals from hydrogen-rich (H_2_/CO = 2) syngas.

## Supporting Information Available

Further experimental details, computational details, and additional results on the DFT calculations; and supporting Figures S1–S24 and supporting Tables S1–S7 (PDF)
Cartesian coordinates for optimized DFT structures (PDF)
