# Supplementary material for: Design of Cobalt Fischer–Tropsch Catalysts for the Combined Production of Liquid Fuels and Olefin Chemicals from Hydrogen-Rich Syngas
Source: ACS Catal. 2021 Apr 5;11(8):4784–98. doi: 10.1021/acscatal.0c05027 (PMC8056389; doi:10.1021/acscatal.0c05027)
Supplement: Supplementary file 1 — cs0c05027_si_001.pdf [file cs0c05027_si_001.pdf]

**Supporting Information**

# Design of Cobalt Fischer-Tropsch Catalysts for the Combined Production of Liquid Fuels and Olefin Chemicals from Hydrogen-rich Syngas

Kai Jeske[1], Ali Can Kizilkaya[2], Iván López-Luque[3], Norbert Pfänder[4], Mathias Bartsch[5], Patricia Concepción[3], Gonzalo Prieto[1,3,*]

[1] Max-Planck-Institut für Kohlenforschung, Kaiser-Wilhelm-Platz 1, 45470 Mülheim an der Ruhr, Germany.

[2] Department of Chemical Engineering, Izmir Institute of Technology, Gülbahçe Kampüsü, 35430 Izmir, Turkey.

[3] ITQ Instituto de Tecnología Química, Universitat Politècnica de València-Consejo Superior de Investigaciones Científicas (UPV-CSIC), Avenida de los Naranjos s/n, 46022 Valencia, Spain.

[4] Max-Planck-Institut für chemische Energiekonversion, Stiftstraße, 45470 Mülheim an der Ruhr, Germany.

[5] Faculty of Physics and CENIDE, Universität Duisburg-Essen, 47048 Duisburg, Germany.

[*]Email: prieto@mpi-muelheim.mpg.de; prieto@itq.upv.es

**<u>Table of contents</u>** *Page*


| | |
|---|---|
| **1. Experimental methods** | **S3** |
| ***Figures EM 1-4*** | **S13** |
| **2. Computational methods** | **S17** |
| ***Figures CM 1-6*** | **S19** |
| **3. Supplementary Figures** | **S22** |
| ***Figure S1*** | **S22** |
| ***Figure S2*** | **S23** |
| ***Figure S3*** | **S24** |
| ***Figure S4*** | **S25** |
| ***Figure S5*** | **S26** |
| ***Figure S6*** | **S27** |
| ***Figure S7*** | **S28** |
| ***Figure S8*** | **S29** |
| ***Figure S9*** | **S30** |
| ***Figure S10*** | **S31** |
| ***Figure S11*** | **S32** |
| ***Figure S12*** | **S33** |
| ***Figure S13*** | **S34** |
| ***Figure S14*** | **S35** |
| ***Figure S15*** | **S36** |
| ***Figure S16*** | **S37** |
| ***Figure S17*** | **S38** |
| ***Figure S18*** | **S39** |
| ***Figure S19*** | **S40** |
| ***Figure S20*** | **S41** |
| ***Figure S21*** | **S42** |
| ***Figure S22*** | **S43** |
| ***Figure S23*** | **S44** |
| ***Figure S24*** | **S46** |
| **4. Supplementary Tables** | **S47** |
| ***Table S1*** | **S47** |
| ***Table S2*** | **S48** |
| ***Table S3*** | **S49** |
| ***Table S4*** | **S50** |
| ***Table S5*** | **S51** |
| ***Table S6*** | **S51** |
| ***Table S7*** | **S52** |
| **5. References** | **S53** |
| **6. Structure files (DFT calculations)** | **S54** |

## 1. Experimental methods

### 1.1. Catalyst synthesis

#### 1.1.1. Synthesis of γ-$Al_2O_3$ supports

Mesoporous γ-$Al_2O_3$ supports were received from Sasol Materials in microparticulate form, or synthesized by calcination of the corresponding microparticulate high-purity pseudo-boehmite precursors in a muffle oven at 823 K with a 0.5 K $min^{-1}$ heating rate from room temperature. For the synthesis of the bimodal meso-macroporous γ-$Al_2O_3$ support, a high-purity dispersible, nanosized pseudo-boehmite precursor (75% $Al_2O_3$, Disperal P2, Sasol Materials) was used as precursor and a polyethyleneglycolether non-ionic surfactant (Tergitol 15-S-7, Sigma-Aldrich, CAS: 84133-50-6) was employed as porogen to synthesize the multimodally porous γ-$Al_2O_3$ support. First, a synthesis gel was prepared by dispersing the pseudo-boehmite precursor in a solution of the surfactant in DI water to achieve a final gel molar composition of Al:EO:$H_2O$= 1:8.1:49, where EO represents the ethylenoxide building units in the polymer (ca. 7 mol EO/mol surfactant). Compared to previous reports from our group[1] the gel composition was set to have a lower porogen concentration in order to obtain an alumina material with higher mechanical stability compared to yet more open trimodally porous meso-macro-macroporous structures. The gel was stirred vigorously at room temperature using a laboratory vertical stirrer (450 rpm) for 5 hours, transferred into a polypropylene autoclavable bottle and treated hydrothermally at 343 K in an oven for 48 h. The resulting gel was transferred into an evaporation dish and let dry at 343 K for 72 hours in an oven with internal air circulation. Finally, the solid was transferred into a muffle oven, further dried at 393 K for 3 h, and then heated to 823 K (0.5 K $min^{-1}$) for the crystallization of γ-$Al_2O_3$ and the combustion of the organic porogen. The muffle oven was equipped with convective air-extraction as required to rapidly evacuate volatile organic material and avoid the generation of igniting gas mixtures in the chamber. After calcination, the solid was sieved to the 0.2-0.4 mm fraction which was further employed for the synthesis of cobalt-based catalysts.

#### 1.1.2. Synthesis of Co-based FT catalysts

Catalysts were prepared by incipient wetness impregnation of the porous γ-$Al_2O_3$ support previously sieved to 0.2-0.4 mm particle size. Mesoporous γ-$Al_2O_3$ supports were obtained by dehydration of high-purity pseudo-boehmite precursors (kindly provided by Sasol Germany) at 823 K (2 K $min^{-1}$ heating rate) for 5 h or directly provided also by Sasol, Germany. The synthesized meso-macroporous support material or strictly

mesoporous commercial γ-$Al_2O_3$ granules were first dried under vacuum (423 K) for 2 hours before impregnation under static vacuum with an aqueous solution containing $Co(NO_3)_2 \cdot 6H_2O$ (1.5 M, Sigma-Aldrich, ≥98%, CAS: 10026-22-9) and ruthenium (III) nitrosyl nitrate (in dilute nitric acid, Sigma-Aldrich, CAS: 34513-98-9) to achieve an atomic ratio of Ru/Co=0.007, which was further acidified with 0.25 vol% $HNO_3$ (69-70 vol.% in $H_2O$, J.T. Baker, ≥99%, CAS: 7697-37-2). The volume of solution applied was equivalent to 90% of the total mesopore volume of the support as determined by $N_2$ physisorption. After impregnation, the solid was dried in a tubular reactor at 343 K under Ar flow (200 $cm^3$ $g_{cat}^{-1}$ $min^{-1}$, Air Liquide, 99.999%, CAS: 7440-37-1) for 10 hours and the nitrate precursors further decomposed at 623 K for 4 h under vertical downward Ar flow (heating rate of 1 K $min^{-1}$). Several impregnation/calcination cycles were performed when needed to adjust the total metal loading in the catalysts. After each further impregnation step the total mesopore volume was corrected by the volume of the metal species previously deposited in the pores, assuming a density of 6.1 $cm^3$ $g^{-1}$ ($Co_3O_4$).

### 1.1.3. Synthesis of promoted Co-based FT catalysts

The required amount of the unpromoted catalyst (in its as-calcined form) was impregnated by slurring the previously dried CoRu/$Al_2O_3$ catalyst, in its as-synthesized (oxidic) form, with an aqueous solution of the promoter/s nitrate precursor/s in 0.5 M $HNO_3$ (22.2 $cm^3$ of solution per unit $cm^3$ of solid pore volume). The concentration of the promoter nitrate precursor in the impregnating solution was adjusted to achieve preset surface-specific contents in the range of 0.4-4 $M_{at}$ $nm^{-2}$, where M denotes the alkali metal or lanthanide elements studied as promoters. For instance to achieve a nominal surface-specific promoter loading of 1.0 $Pr_{at}$ $nm^{-2}$ on a CoRu/γ-$Al_2O_3$ catalyst with a support specific surface area of 187 $m^2$ $g_{Al2O3}^{-1}$, 144.5 mg of $Pr(NO_3)_3 \cdot 6H_2O$ (Sigma-Aldrich, 99.9%, CAS: 15878-77-0) were applied per gram of solid. Similarly, the following nitrate precursors were used to incorporate alternative lanthanide and alkali oxide promoters on the cobalt-based FT catalysts: $Sm(NO_3)_3 \cdot 6H_2O$ (Sigma-Aldrich, 99.9%, CAS: 13759-83-6), $La(NO_3)_3 \cdot 6H_2O$ (Sigma-Aldrich, 99.99%, CAS: 10277-43-7), $NaNO_3$ (Sigma-Aldrich, >99%, CAS: 7631-99-4), $KNO_3$ (Sigma-Aldrich, >99%, CAS: 7757-79-1) and $CsNO_3$ (Fisher Scientific, 99.99%, CAS: 7789-18-6). After wet impregnation, the water solvent was removed in a rotary evaporator at 323 K and the solid was subsequently transferred to a tubular reactor and calcined at 623 K for 4 h under vertical downward synthetic air flow (Air Liquide, 20.5 ± 0.5 mol% $O_2$ in $N_2$, 99.999%, CAS: 132259-10-0) using a heating rate of 1 K $min^{-1}$ and a gas flow of 200 $cm^3$ $g_{solid}^{-1}$ $min^{-1}$.

### 1.2. Catalyst characterization

#### 1.2.1. $N_2$ physisorption

$N_2$ physisorption isotherms were recorded at 77 K using a Micromeritics 3Flex V4.04. Prior to the measurements, samples were dried *in situ* at 523 K under vacuum ($10^{-3}$ mbar) for 12 h (10 K $min^{-1}$). The Brunauer-Emmett-Teller (B.E.T.) method was used to determine surface areas from the 0.05-0.30 $P/P_0$ regime of the recorded isotherms. Pore volumes were determined from the adsorbed $N_2$ volume detected at equilibrium point $P/P_0$ of 0.95 from the adsorption branch of the isotherm. Pore size distributions were determined using the Barrett-Joyner-Halenda (B.J.H.) method applied to the desorption branch of the isotherm. The average mesopore diameter was defined as the peaking value of the pore size distribution.

#### 1.2.2. Hg intrusion porosimetry

Mercury intrusion porosimetry experiments were performed in a Micromeritics AutoPore IV 951 apparatus. 80-150 mg of sample (0.4-0.6 mm) were dried at 383 K for 72 h before the measurement. The intrusion-extrusion isotherms were recorded at room temperature in the pressure range of $6.9 \cdot 10^4$-$4.1 \cdot 10^4$ Pa with an equilibration rate of 0.1 μL $g^{-1}$ $s^{-1}$. For the determination of pore diameter and volume, a geometrical pore model was considered, with a Hg density of 13.55 g $cm^{-3}$ and a contact angle of 141 degree.

#### 1.2.3. Powder X-ray diffraction (XRD)

X-ray powder diffractograms were recorded on a Stoe STADI P transmission diffractometer (Mo $K_\alpha$ radiation, 0.7093 Å, goniometer radius 240 mm) equipped with a Mythen1K solid state strip detector in transmission mode. For each measurement, finely ground powdered samples was filled into a 0.5 mm ø borosilicate glass capillary and loaded onto the sample stage, where the capillary was spinning (~120 rpm) while the measurement was performed from 5-50 2θ degrees, 2° $step^{-1}$, 40s $step^{-1}$. Catalysts in their as-reduced state were transferred and sealed into the capillaries in an Ar-filled glove box under exclusion of air.

#### 1.2.4. X-ray photoelectron spectroscopy (XPS)

X-ray photoelectron spectra were collected by using a SPECS spectrometer equipped with a Phoibos 150 MCD-9 detector and using a non-monochromatic (AlKα=1486.6 eV) X-ray source, an analyzer pass energy of 30 eV, and a X-ray power of 100 W under an operating pressure of $10^{-4}$ mPa. Prior to the XPS experiments,

the samples were submitted to reduction activation under $H_2$. First, *ex situ* catalyst reduction was performed on ca. 100 mg of catalyst in the sieve fraction 0.2-0.4 mm in a quartz tubular reactor using $H_2$ (70 $cm^3$ $min^{-1}$) at 673 K for 5 h (heating rate of 1 K $min^{-1}$). After reduction, the catalyst bed was let cool down to room temperature and the metal nanoparticles were surface passivated by exposure to flow of 1%$O_2/N_2$ for 1 h at room temperature. The surface passivated catalyst was press-conformed as a pellet, mounted on a stainless steel XPS sample holder and inserted in the high-pressure catalytic reactor (HPCR) directly connected to the XPS analysis chamber under vacuum. Further *in situ* reduction (to reverse surface passivation) was performed in this reactor cell under flow of $H_2$ (25 $cm^3$ $min^{-1}$) at 673 K for 2 h (5 K $min^{-1}$). In certain instances, the catalyst pellet was allowed to cool down to 373 K, a flow of syngas ($H_2$:CO:Ar=45:45:10, 52 mL $min^{-1}$) admitted into the reactor cell, the pressure increased to 10 bar and the temperature raised back to 473 K in order to emulate Fischer-Tropsch reaction conditions for 2 h. Following these *in situ* treatments, the sample was transferred to the XPS chamber under UHV conditions, without exposure to air. Data processing was performed in CASA XPS software using Shirley-type backgrounds. Gaussian-Lorentzian (GL(30)) profiles were used for the fitting of oxide components. For metallic Co2p components, an asymmetric line (LA (1,2,50)) has been applied together with two plasmon loss peaks at 3.0 eV and 5.0 eV, respectively, above the main peak. Binding energies were referenced to the Al2p signal associated to the $\gamma$-$Al_2O_3$ support at 74.1 eV.

### 1.2.5. Temperature-programmed desorption of carbon dioxide ($CO_2$-TPD)

Temperature-programmed desorption of carbon dioxide ($CO_2$-TPD) was carried out on a TPD/2900 setup from Micromeritics. 100 mg of catalyst, sieved in the 0.4-0.6 mm particle size range, were first reduced *in situ* under flow of 10% $H_2$/Ar (50 $cm^3$ $min^{-1}$) at 673 K for 2 h (heating rate 10 K $min^{-1}$), followed by flushing with He (100 $cm^3$ $min^{-1}$) for 1 h at 673 K to remove any physisorbed hydrogen, and then cooling down to room temperature under He flow. $CO_2$ was chemisorbed by admitting 0.5 $cm^3$ pulses of $CO_2$ (Linde, 99.995%) at room temperature until surface saturation, as determined by the repeatability of the TCD signal of the $CO_2$ pulses downstream of the catalyst packed bed. After $CO_2$ dosage had been completed, the sample was purged with He (100 $cm^3$ $min^{-1}$) for 15 min to eliminate any weakly adsorbed $CO_2$, and the temperature increased to 1073 K using a heating rate of 10 K $min^{-1}$. The $CO_2$ desorption profile was registered by tracking the molecular ion with m/z=44 in a quadrupole mass spectrometer (Pfeiffer) connected online, downstream of the TPD setup.

### 1.2.6. $H_2$-Temperature-programmed reduction

Hydrogen temperature-programmed reduction ($H_2$-TPR) experiments were performed in a Micromeritics Autochem 2910 device to determine the reducibility of the cobalt catalysts. About 45 mg of sample were initially flushed with Ar flow (50 $cm^3$ $min^{-1}$) at room temperature for 30 min, then the gas was switched to 10 vol% $H_2$ in Ar and the temperature increased up to 1123 K at a heating rate of 10 K $min^{-1}$. A downstream acetone/dry ice trap was used to retain the water generated during the reduction. The $H_2$ consumption rate was monitored by a thermal conductivity detector (TCD) previously calibrated via the injection of known volumes of hydrogen using a gas syringe. The cobalt loading in the catalysts was determined from the total hydrogen consumption assuming all cobalt to be present as $Co_3O_4$ in the starting calcined catalysts (as confirmed with powder X-ray diffraction experiments, not shown), therefore a reduction stoichiometric $H_2$/Co molar ratio of 4/3 was applied. The hydrogen consumption associated to the reduction of the Ru promoter was considered negligible.

### 1.2.7. $H_2$ chemisorption

Surface-exposed metal surface areas were determined using $H_2$ chemisorption at 373 K in an ASAP 2010C Micromeritics by plateau extrapolation to zero CO pressure.[2] Prior to $H_2$ dosing, the sample (ca. 0.1 g of supported cobalt catalyst) was reduced *in situ* under flow of pure $H_2$ at 673 K for 5 hours (heating rate from RT of 2 K $min^{-1}$). After reduction, the sample was degassed at 1.3 Pa and the temperature lowered to 373 K to record the $H_2$ chemisorption isotherm. $Co^0$ surface areas were determined from the total amount of chemisorbed $H_2$, and the Co loading as determined by $H_2$-TPR. The contribution of $Ru^0$ to the overall metal content was considered insignificant. A surface H/Co=1 stoichiometry and a surface atomic density of 14.6 $Co_{at}$ $nm^{-2}$ were considered.

### 1.2.8. Chemical analysis by Energy-dispersive X-ray spectroscopy (EDS)

In order to quantify bulk promoter contents, energy-dispersive X-ray spectroscopy (EDS) was performed on a Hitachi S-3500N scanning electron microscope. The catalyst samples were ground and then applied onto a pin-stub SEM mount coated with double-adhesive-face conductive carbon-tab. EDS spectra of areas of 1 $mm^2$ were scanned using an Oxford Pentafet 10 $mm^2$ detector to make sure a statistically relevant number of catalyst particles was jointly analyzed.

### 1.2.9. Tomographic Focused-Ion-Beam Scanning-Electron Microscopy (FIB-SEM)

Microparticles (200-400 µm) of the unpromoted CoRu/AOmM catalyst have been embedded in a low-viscosity resin (Spurr) and the resin cured at 343 K for 12 h. The resin-embedded sample block has then been trimmed and polished in an ultramicrotome (Reichert Ultracut) using a diamond knife (Diatome), and then mounted on a SEM stub with conductive colloidal graphite adhesive. The stub-mounted sample has been sputter-coated with a ca. 20 nm gold overlayer using a BAL-TEC SCD 005 coater to achieve full conductive joints and minimize local charging artefacts during SEM imaging. Focused Ion Beam-Scanning Electron Microscopy (FIB-SEM) experiments were performed in a Helios NanoLab™ 600 (FEI) microscope. First, a protective Pt layer (0.2 µm thickness) was deposited on the region of interest (ROI) using the gas injection system. Then, the $Ga^+$ ion gun, operated at 30 kV and 21 nA, was used to mill a staircase front trench and two lateral trenches (ca. 50 µm depth), delimiting the volume to be imaged, as well as a cross fiducial marker (ca. 0.5 µm depth) on the top surface of the ROI to be used for automated image recognition and drift correction to maintain a constant slice thickness during the slice-and-image procedure. Serial sectioning combined with SEM imaging was carried out using an in-house developed automated routine executed using the RunScript software (FEI) with specimen drift and beam shift corrections after each milling/imaging cycle. Slices with nominal thickness of 112 nm were milled off using the $Ga^+$ FIB gun operated at 30 kV and 9.7 nA. The corresponding SEM micrographs of the consecutively exposed cross-sections were recorded with an ETD secondary electron detector while the electron gun was operated at 2 kV and 0.34 nA.

The stack of raw micrographs was first corrected for the foreshortening caused by the tilt angle between the specimen cross-section and the SEM detector (52° in the dual beam microscope employed in this study). Next, low-pass Gaussian and de-flicker filters were applied to reduce noise and correct inter-micrograph gray-value gradients within the stack of images, respectively, followed by a 2x2 pixel binning operation within the xy plane to enlarge the final voxel size and facilitate further computational processing (3D image analysis). After stack alignment using an iterative cross-correlation algorithm, the reconstructed tomograms were segmented using a watershed algorithm,[3] followed by fine adjustment of the automatically recognized volumes via controlled erosion-dilation functions, to remove artefact material "islands", as well as manual threshold adjustment to correct for local gray-scale gradients created by either curtaining effects or shadowing phenomena. After tomogram binarization, a 3D local Euclidean distance transform[4] was propagated along the three-dimensional skeleton of the "solid" alumina phase to quantify the shortest distances from any point

within the mesoporous alumina domains to the nearest boundary to the macropore network. Similarly, analysis of local maxima and minima in the 3D local distance map within the volume of connected voxels assigned to the macropore network served to derive the pore network model (PNM)[5] in Avizo (Thermo Fischer Scientific), where volumes were also surface-rendered for 3D visualization.

### 1.2.10. High-Angle Annular Dark-Field Scanning-Transmission Electron Microscopy (HAADF-STEM)

Before microscopy observation, samples were embedded in a low viscosity resin (Spurr, hard composition) and hardened at 343 K. Then nanometer thin slices (nominal thickness of 150 nm) were obtained with a Diatome diamond knife mounted on a Reichert Ultracut ultramicrotome and collected on a copper TEM grid (300 mesh) covered with a lacey carbon film. High-angle annular dark field (HAADF) STEM micrographs and EDS elemental maps were acquired using a beam spherical aberration-corrected ($C_s$) Hitachi HD-2700 dedicated Scanning Transmission Electron Microscope (STEM) equipped with a cold field-emission gun and two EDAX Octane T Ultra W EDS detectors and operated at 200 kV.

### 1.2.11. *In situ* FTIR spectroscopy

*In situ* FTIR experiments were performed applying CO as a surface probe molecule in a Bruker Vertex70 spectrometer using a DTGS detector and acquiring at 4 $cm^{-1}$ resolution. Prior to the experiments, catalysts (ca. 100 mg, 0.2-0.4 mm granule size) were first reduced *ex situ* in a tubular quartz reactor under flow of hydrogen (70 $cm^3$ $min^{-1}$) at 673 K using a heating rate of 1 K $min^{-1}$, followed by a surface metal passivation treatment at room temperature under flow of 1% $O_2/N_2$ for 1 h. Then the samples were pressed into self-supported wafers (5-10 mg $cm^{-2}$) for transmission FTIR and mounted into the IR cell. The pelletized samples were reduced *in situ* in the cell under flow of $H_2$ at 673 K for 1 h to reverse the surface passivation metal oxide overlay. In a first set of experiments, CO was used to probe surface metal sites on the *in situ* reduced catalysts using a home-made quartz cell fitted with Thallium bromoiodide (KRS-5) windows and connected to a vacuum dosing system which features both a rotary-vanes and a turbo-molecular vacuum pumps and a high-precision gas dosing facility. Following *in situ* reduction, the samples were outgassed under vacuum (ca. $10^{-5}$ mbar) at 723 K (50 K above the reduction temperature) to allow for the displacement of any hydrogen remaining on the metal surface and then cooled down to RT under vacuum. Subsequently, CO was added at increasing pressures (0.4-260 mbar) and the IR spectrum recorded after each dosage. In another set of

experiments, CO was applied as a probe for surface Lewis acid sites at 110 K. At this temperature, CO probes both metal centers as well as surface sites on the oxide carriers, however, saturation coverage on the former is achieved at very low $P_{CO}$ and thus the υ(CO) spectral region for surface metal carbonyls was only analyzed in those CO-FTIR experiments performed at RT. Low temperature experiments were carried out in a stainless steel cell featuring also KRS-5 windows, which allows *in situ* treatments in controlled atmospheres and temperatures from 97 K to 773 K. After *in situ* reduction and evacuation, the sample was cooled to room temperature under vaccuum. Next, $N_2$(l) was admitted into the jacket of the cell to lower the temperature to 110K and CO (Linde HiQ, carbon monoxide 3.7 purity) was dosed from an aluminum can on the catalyst surface at stepwise increasing pressures up to 2 mbar. Spectra deconvolution were performed using Voigt functions on difference spectra obtained by subtracting the spectrum recorded directly after *in situ* sample activation in the IR cell.

### 1.3. Catalyst testing in the Fischer-Tropsch synthesis (FTS)

The FTS catalytic tests were performed at 20 bar and 473 K in a fixed-bed reactor setup schematically shown in Figure **EM1**. It is equipped with mass-flow controllers (Bronkhorst, MFCs, ②) to feed $H_2$ (Air Liquide, 99.999%, CAS: 1333-74-0) and pre-mixed syngas (30 vol.% CO, 60 vol.% $H_2$, 10 vol% Ar as internal standard, Air Liquide, 99.99%), two-finned copper elements with embedded 180 W heating cartridges (⑧) to heat the stainless steel (316L grade) fixed-bed micro-reactor (inner diameter, *i.d.* = 12 mm; ⑨) two thermocouples (K-type, 0.5 mm, ⑦) placed at the start and at the end of the catalyst bed as part of two PID feedback loops to control the temperature inside the reactor, and an online gas chromatograph (modified Agilent 7890B, ⑯) for product analysis.

Upstream of the syngas MFC, the syngas stream was first passed over an activated carbon bed (Norit, ⑤) at 30 bar and room temperature to remove metal-carbonyl impurities that might be entrained from the pressurized gas cylinder, and then through a 316L grade stainless steel (length, *l* = 300 mm, *i.d.* = 3 mm inner diameter) capillary loop (⑥) maintained at 473 K to pre-heat the syngas. The free volume in the reactor was reduced by inserting two 316L grade stainless-steel spacers (⑩), one (*l* = 62 mm) upstream of the catalyst bed, and another (*l* = 50 mm) downstream of the bed. The spacers were separated from the catalyst bed using two quartz wool plugs (*l* = 5 mm, ⑪). Below the first Quartzwool plug, a 3.4 $cm^3$ layer of SiC granules (Alfa Aesar, 46 grit, CAS: 409-21-2) (⑫) was placed to additionally pre-heat syngas and help establish plug-flow before entering the catalyst bed. The catalyst bed (⑬) consisted of an amount of catalyst equivalent to 100

mg of $Co^0$ as determined by $H_2$-TPR. This amount of supported catalyst (Ø = 200-400 µm) was diluted with 6.2 $cm^3$ of SiC granules (Alfa Aesar, 46 grit, 409-21-2) to improve heat transfer in the catalyst bed.

Before the FTS tests, the metal species in the catalyst were reduced in $H_2$ flow (200 $cm^3$ STP $min^{-1}$) at 673 K (2 K $min^{-1}$ to 423 K, followed by 0.83 K $min^{-1}$ to 673 K) for 5 h at ambient pressure. After reduction, the reactor was cooled to 423 K in $H_2$ flow, followed by switching the flow to the synthetic syngas mixture (150 $cm^3$ STP $min^{-1}$; weight hourly space velocity, *WHSV* = 3.6–11.0 $h^{-1}$). The system was then pressurized to 20 bar using a membrane dome regulator (GO regulator), and the reactor temperature was increased to 473 K (0.15 K $min^{-1}$). The start of the temperature ramp from 423 K to 473 K in syngas was marked as time-on-stream = 0 h. Both reactor design and startup protocols have been optimized to ensure an isothermal catalyst bed operation. After at least 24 h time-on-stream, the syngas flow was adjusted to determine catalyst performance at a 20±3% CO conversion level.

Downstream of the reactor, two consecutive cold traps (⑭) were set at temperatures of 423 K and 373 K at the reaction pressure to collect heavy hydrocarbon products and water. All other downstream tubing was heated to 443 K to prevent the condensation of reaction products. All gaseous components of this gas stream leaving the traps were depressurized after passing the dome pressure regulator and were analyzed by online gas chromatography. The gas chromatograph is equipped with two sampling loops, one of which feeds into a capillary column (Restek RTX-1, 60 m) equipped with an FID detector, while the other feeds into two consecutive packed-bed columns (HS-Q 80/120, 1x m + 1x 3 m) equipped with a TCD for the analysis of $H_2$, $CO_2$, and $C_2$-$C_3$ hydrocarbons. Along this analysis channel, a molecular sieve column is used for the separation of Ar, $CH_4$, and CO, which are detected using an additional TCD. Figures EM2 and EM3 show representative product chromatograms obtained online for the gas stream and offline for the waxy products collected in the high-temperature trap, respectively, testing a $PrO_x$-promoted meso-macroporous CoRu/γ-$Al_2O_3$ catalyst. CO, $CH_4$, and $CO_2$ were quantified using TCD response factors relative to Ar. CO conversion ($X_{CO}$), cobalt-time-yield (CTY) and metal surface-specific time-yield ($A_{Co}$-CTY or *turnover frequency* (TOF)) were used to express catalytic activity (Equations 1-4). Selectivity to $C_{5+}$ hydrocarbon products was determined by deducing the selectivity to $C_{4-}$ carbon-containing products, including $CO_2$, from a closed carbon balance (Equations 5 and 6). For selected catalysts, longer tests (ToS>80 h) were performed in order to collect sufficient amounts of condensed products in the post-reactor traps to enable a reliable analysis of liquid (water-soluble and oil) as well as solid wax product phases. Liquid and solid hydrocarbons collected

from the trap kept at 373 K and 20 bar were separated by decanting, the individual liquid phases were further purified by centrifugation (9000 rpm, 15 min) to collect waxes and obtain clear polar and non-polar phases which were weighed separately. Solid wax hydrocarbons collected from the trap kept at 423 K and 20 bar were blended together with those wax fractions collected by centrifugation of the liquid products, weighed, and dissolved in $CS_2$ (≥99%, Sigma-Aldrich, CAS: 75-15-0). Acetone (HPLC grade, ≥99.9%, Sigma-Aldrich, CAS: 67-64-1) was added in the case of aqueous phase samples and 2-methyl heptane (>98.0%, Sigma-Aldrich, CAS: 592-27-8) was added in the case of non-polar oil and wax solution samples as gas chromatography standards, respectively. Offline gas chromatography of the organic hydrocarbon samples was performed on a second Agilent 7890B gas chromatograph equipped with a capillary column (Restek RTX-1, 60 m) which elutes into an FID. Offline gas chromatography of aqueous phase products was performed on a third Agilent 7890B gas chromatograph equipped with a high-polarity polyethylene glycol (DB-Waxetr, iD=0.25 mm, film thickness=0.25 µm, 30 m) which elutes into an FID. Figure EM2 depicts schematically the experimental protocol for offline product analysis. Figures EM3 and EM4 show representative product chromatograms obtained online for the gas stream and offline for the waxy products collected in the high-temperature trap, respectively. Carbon balances closed at 95±3%.

$CO\ conversion\ (X_{CO}) = \left(1 - \frac{\dot{n}_{CO,out}}{\dot{n}_{CO,in}}\right) \cdot 100$ (Equation 1)

$Cobalt\ time\ yield\ (CTY) = \dot{n}_{CO,in} \cdot X_{CO} \cdot m_{Co}{}^{-1}$ (Equation 2)

$Exposed\ cobalt\ surface\ area\ (A_{Co}) = \frac{3}{d_{Co} \cdot \rho_{Co}}$ (Equation 3)

$Cobalt\ surface\ specific\ time\ yield\ (A_{Co} - CTY) = \frac{CTY}{A_{Co} \cdot 3600}$ (Equation 4)

$S_{C1-C4} = \sum_{i=1}^{4} \dot{n}_{CO,out,i} \cdot n_i \div (\dot{n}_{CO,in} - \dot{n}_{CO,out}) \cdot 100$ (Equation 5)

$S_{C5+} = 1 - S_{C1-C4}$ (Equation 6)

$\dot{n}_{CO,in} = molar\ flow\ of\ inlet\ CO$

$\dot{n}_{CO,out} = molar\ flow\ of\ CO\ detected\ by\ GC$

$m_{Co} = cobalt\ mass$

$\rho_{Co} = cobalt\ density$

$n = carbon\ number\ of\ a\ detected\ hydrocarbon$

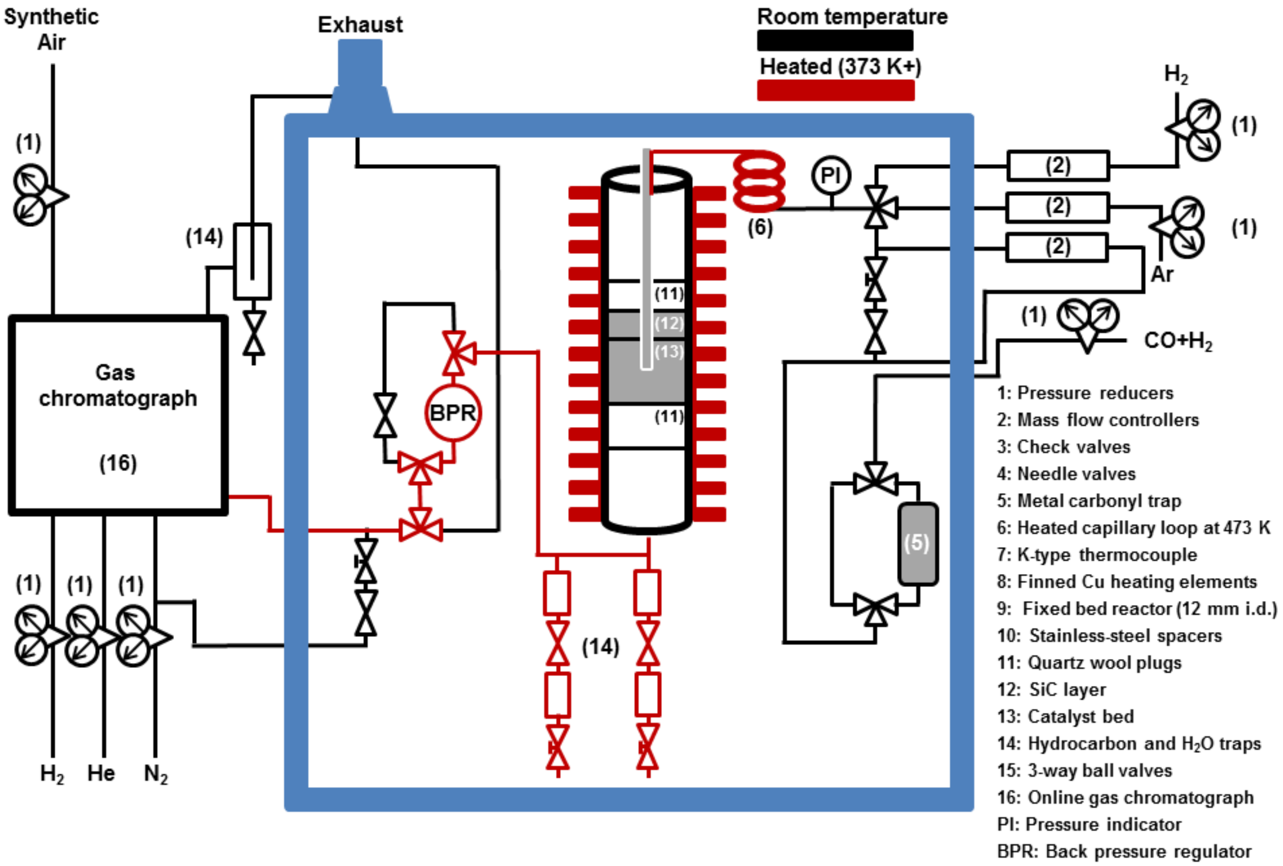


**Figure EM1:** Schematic of the fixed bed reactor setup used to perform Fischer-Tropsch synthesis experiments.

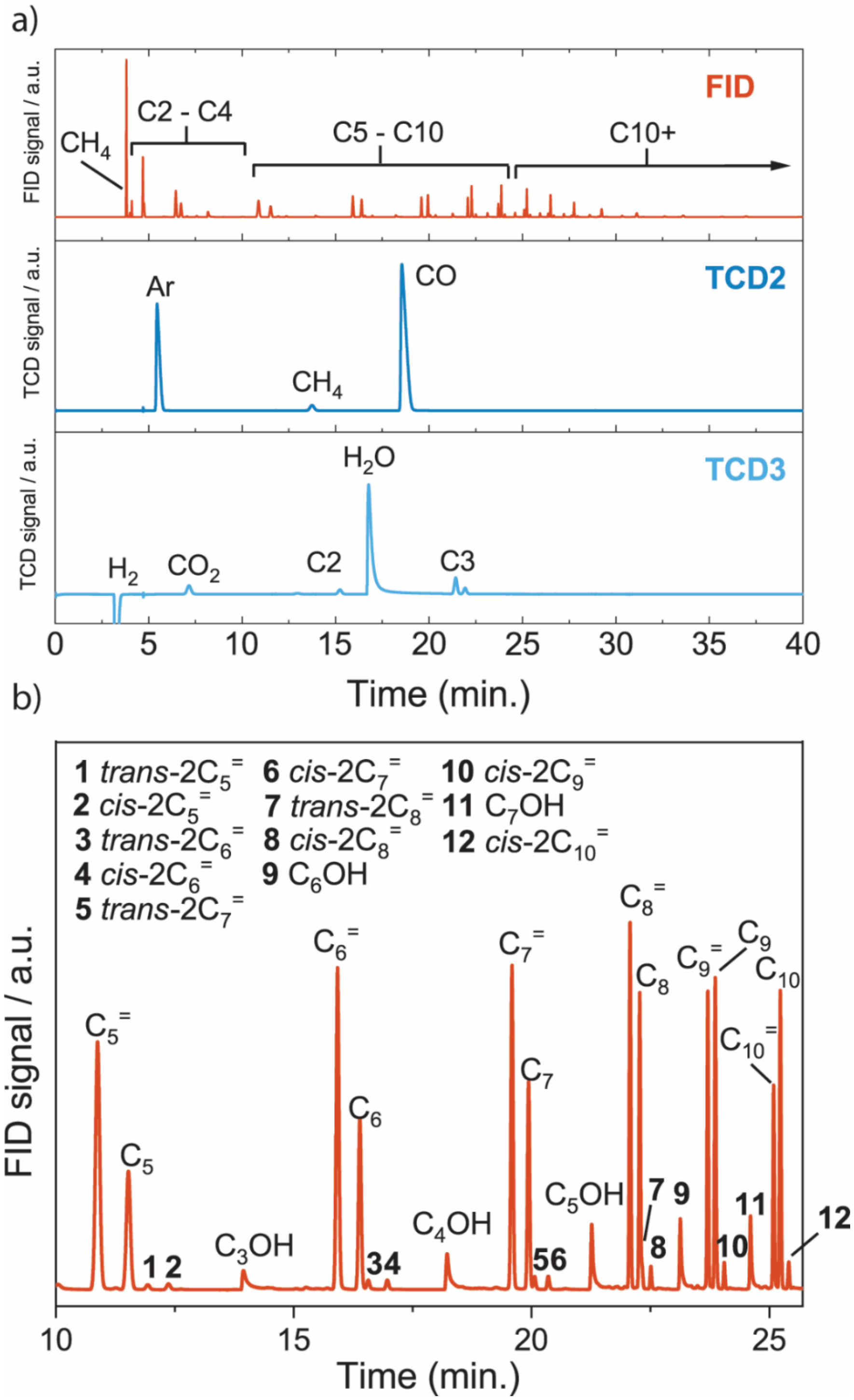


**Figure EM2:** Representative online product chromatograms for a FT catalytic test with 1.0Pr-CoRu/AOmM. a) Hydrocarbon products in the range of $C_1$-$C_{16}$ eluting from a Restek RTX-1 capillary column can be detected in the flame-ionization detector (FID) of the Agilent GC7890B. Permanent gases eluting from a 13X molecular sieve packed column are detected on a first TCD, whereas light hydrocarbon products ($C_2$-$C_3$), as well as water eluting from two consecutive HS-Q 80/120 packed columns are detected on a second TCD. b) Detail view of $C_5$-$C_{10}$ HC products of FID chromatogram shown in (a), highlighting the predominance of α-olefin products in the $C_{5-10}$ range. *trans*-$C_9^=$ and *trans*-$C_{10}^=$ are overlapped by $C_9$ and $C_{10}$, respectively.

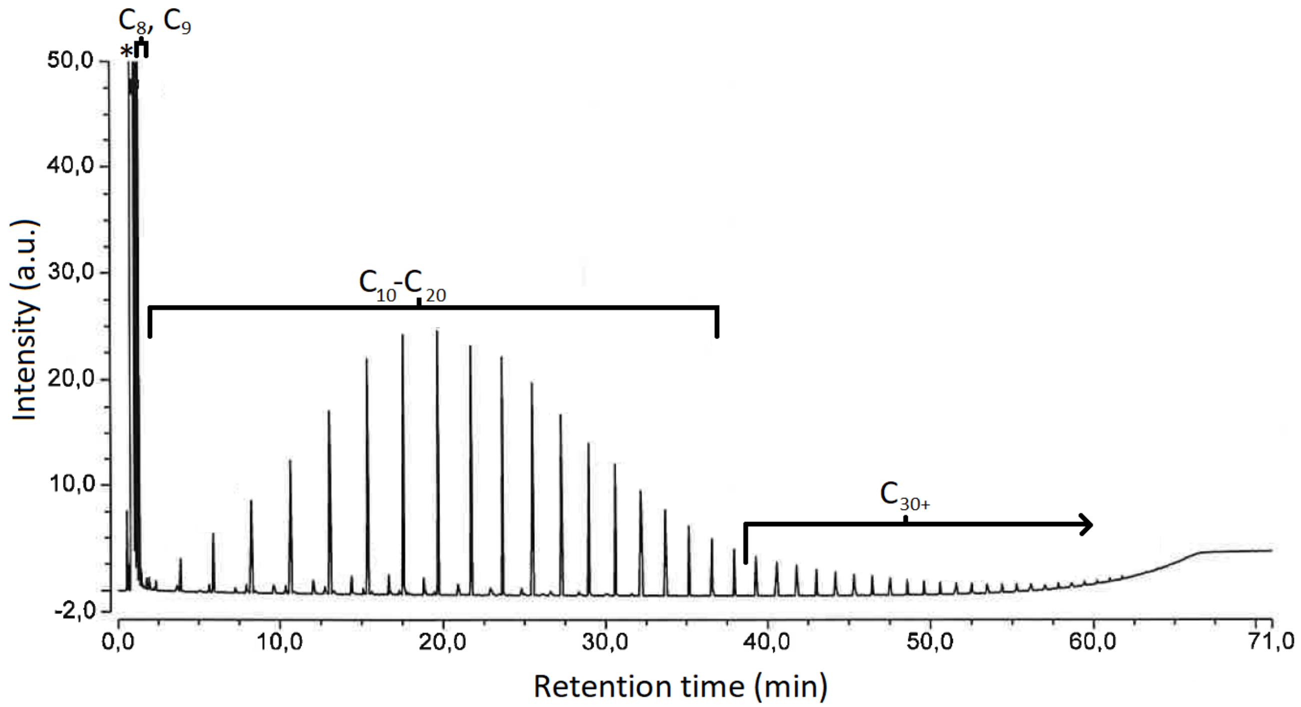


**Figure EM3:** Offline GC analysis of the waxy hydrocarbon product fraction collected over 114 h on-stream with 1.0Pr-CoRu/AOmM following a 48 h run period to establish quasi steady-state conditions (during which trapped hydrocarbons were discarded). Products eluting from a capillary column detected using an FID. Selected components are labelled on the chromatogram. FT wax dissolved in excess of toluene (1:130 mass basis) before analysis. The solvent toluene indicated by asterisk.

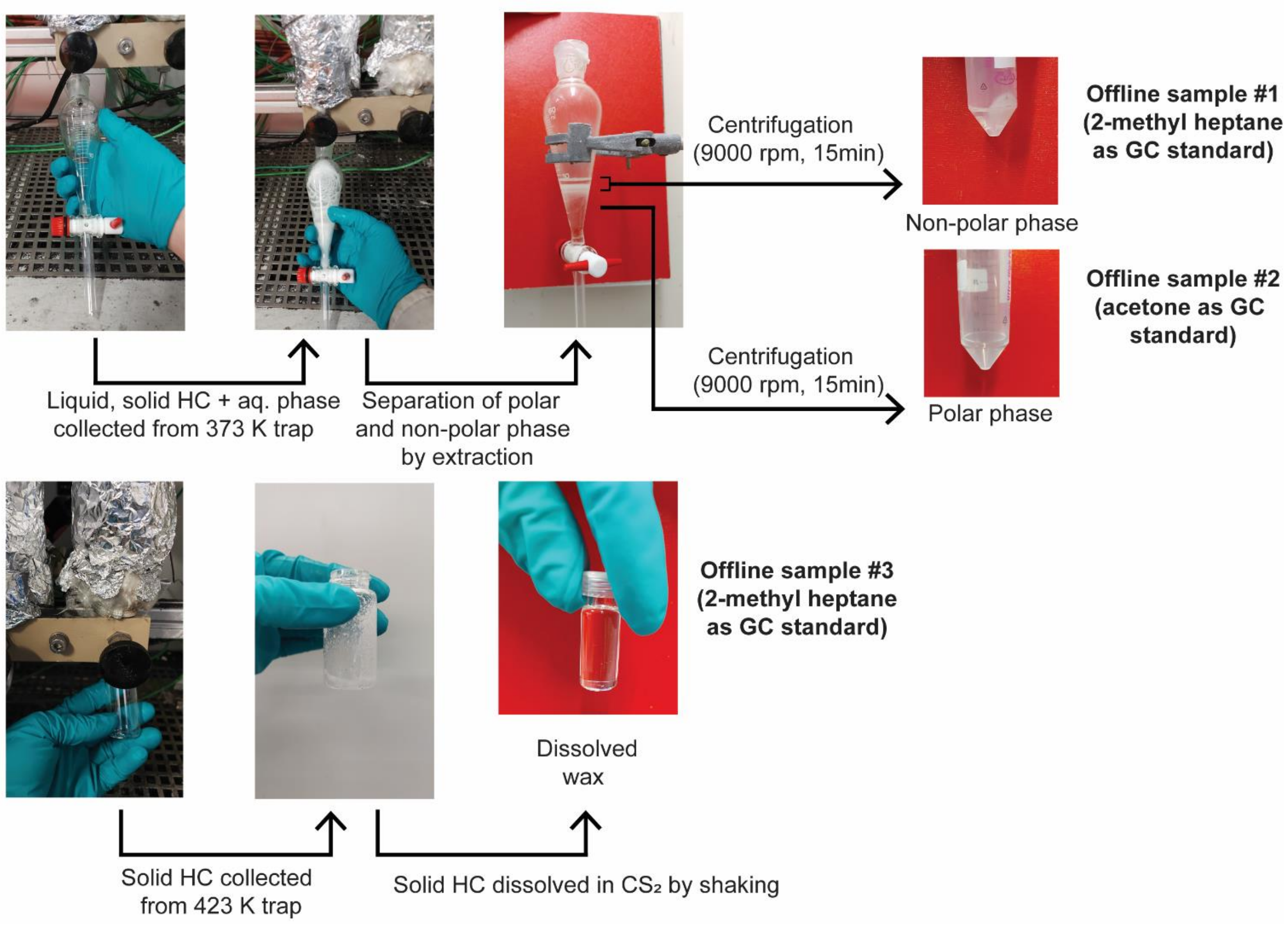


**Figure EM4:** Exemplary sample treatment procedure for offline gas chromatography analysis involving product separation by extraction, centrifugation and admixing known quantities of 2-methyl heptane or acetone.

## 2. Computational methods

Periodic Density Functional Calculations were performed using the Vienna Ab-initio Simulation Package (VASP).[6-7] The exchange-correlation energy was calculated with the PBE[8] form of the generalized gradient approximation (GGA) functional. The electron-ion interaction was modeled by the projector-augmented wave (PAW)[9] method. Spin-polarized calculations were performed to account for the magnetic properties of cobalt with a plane wave cut-off energy of 600 eV.

Surface terraces and step-edges of face-centered-cubic (*fcc*) Co nanoparticles in the supported FT catalysts were modeled with 4 atomic layers-thick Co(111) and 8 atomic layers-thick Co(211) slab models. The *fcc* cobalt polymorph is used as model as it has been reported to be the most stable phase in cobalt nanoparticles with sizes below 110 nm[10] and the predominant polymorph identified by XRD in the reduced FT catalysts. *Fcc* Co nanoparticles adopt preferentially a regular truncated octahedron shape and expose predominantly close-packed (111) terraces and step-edges with a specific B5 geometry.[10] The latter have been previously modelled with the Co(211) stepped surface with good correspondence with experimental data.[11]

The surfaces were cut from a bulk *fcc*-Co structure with an optimized lattice parameter of 3.52 Å. To investigate the coverage dependence of the binding energies for promoter oxide species and adsorbates, p(2x2) and p(3x3) models were used for the Co(111) surface to represent 0.25 ML and 0.11 ML coverages, and p(1x2) and p(1x3) models were used for the Co(211) surface to represent 0.17 ML and 0.11 ML coverages. The reciprocal space was sampled with a (5×5×1) k-points grid for p(2x2)-Co(111) and p(1x2)-Co(211) models and with (3x3x1) k-points grid for p(3x3)-Co(111) and p(1x3)-Co(211) models, automatically generated using Monkhorst-Pack method.[12] A vacuum height of at least 10 Å was inserted between slabs to avoid coupling between successive slabs in the *z*-direction. The atoms at the bottom half of the slabs in the *z*-direction were kept fixed at their pre-optimized positions, while all other atoms were allowed to relax during the optimization of promoters and adsorbates. The structural models were optimized until all the forces acting on atoms are smaller than 0.01 eV/Å. Dipole corrections in *z*-direction were used for all optimizations.

Praseodymium and sodium oxide, representative for the series of alkali and lanthanide oxide promoters studied experimentally, respectively, were modelled as $PrO_2$ and $Na_2O$ monomeric units decorating the cobalt surfaces. The binding energies of the promoters and the adsorption energies of $CO^*$, atomic $H^*$ and $C_2H_2^*$ are reported with respect to their gas-phase structures, i.e. as the energy difference between the slab decorated

with the adsorbate/promoter at a given surface coverage and the slab without the adsorbate promoter plus the adsorbate in the gas phase. Bader analysis[13-15] were performed to investigate changes in electronic charge on cobalt and adsorbates as a result of the deposition $PrO_2$ and $Na_2O$ on Co(111) and Co(211) surfaces.

***2.1 Supplementary computational results***

***2.1.1. Oxide structures on cobalt at high oxide surface coverage***

At 0.25ML surface coverage on Co(111) for the $PrO_2$ promoter, the Pr and one oxygen ion are adsorbed on *hcp* hollow sites of the surface, while the second oxygen ion is bonded only to the Pr (high above a surface top site), pointing away from the surface (Figure CM1a). For $Na_2O$, promoter unit adopts a honeycomb structure, Na ions occupy both *hcp* and *fcc* hollow sites while the oxygen ion is located on the *fcc* site (Figure CM1b).

On the stepped Co(211) surfaces, at 0.17 ML oxide coverage, $PrO_2$ binds to the surface such that one oxygen ion is located on the step-edge, tilted towards the Pr atom, while the other oxygen ion sits on a *hcp* hollow site on the lower terrace and the Pr atom occupies both the step-edge and a *hcp* hollow site on the lower terrace, due to its bulky size (Figure CM2a). This binding geometry is energetically significantly favored (by 230 kJ $mol^{-1}$ ) compared to both oxygens located on the terrace. In the case of $Na_2O$, all ions are located around the step-edge, with O and one Na ion fully covering the step-edge, while the other Na ion is located on the lower terrace on the *fcc* site (Figure CM2b). This structure is found to be the only stable configuration on the surface at this high oxide coverage.

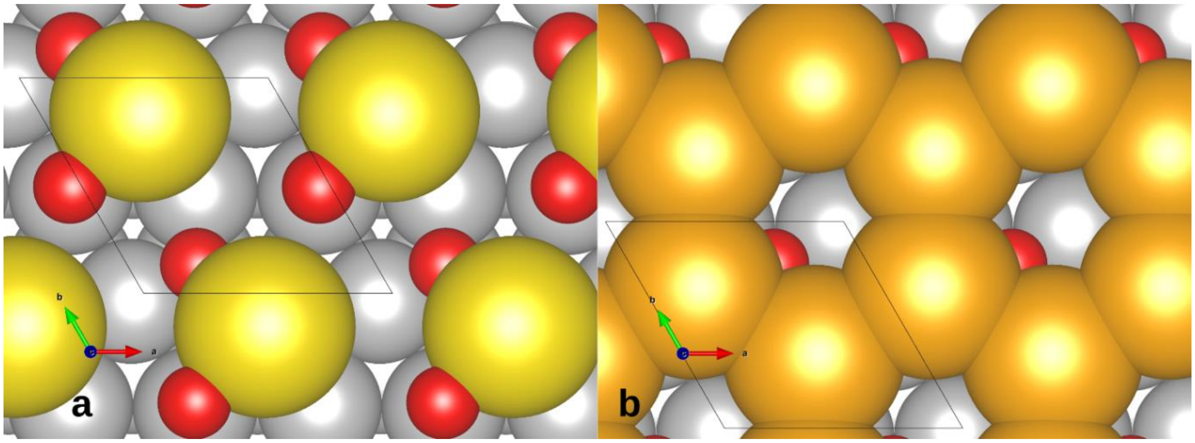


**Figure CM1:** Adsorption structures for a) $PrO_2$ and b) $Na_2O$ promoter units for 0.25 ML coverage on Co(111). Color codes: Pr: yellow, Na: orange, O: red, Co: gray.

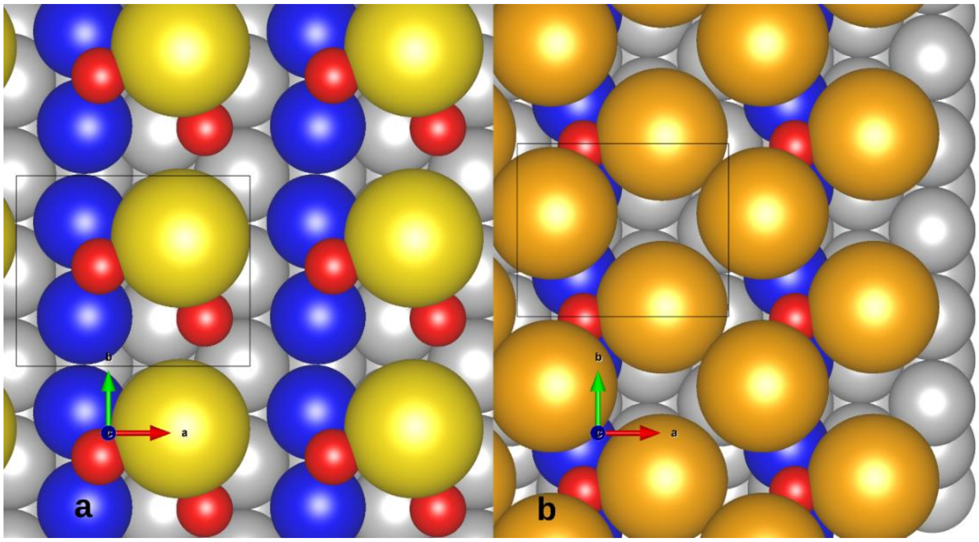


**Figure CM2:** Adsorption structures for a) $PrO_2$ and b) $Na_2O$ promoter units for 0.17 ML coverage on Co(211). Color codes: Pr: yellow, Na: orange, O: red. For clarity, cobalt atoms at the step-edges are shown in blue, while those on the terrace under the edge as shown in gray.

### 2.1.2. *$H^*$ and $CO^*$ adsorption sites on oxide-covered cobalt surfaces*

On oxide-covered Co(111) surfaces, several inequivalent adsorption sites were found to be possible for $H^*$ and $CO^*$. These binding sites are indicated in Figure CM3. Also on the oxide-covered stepped Co(211) surface, four inequivalent adsorption positions were identified for $CO^*$ and $H^*$ with a high preference to be located along the step-edge, as shown in Figure CM4.

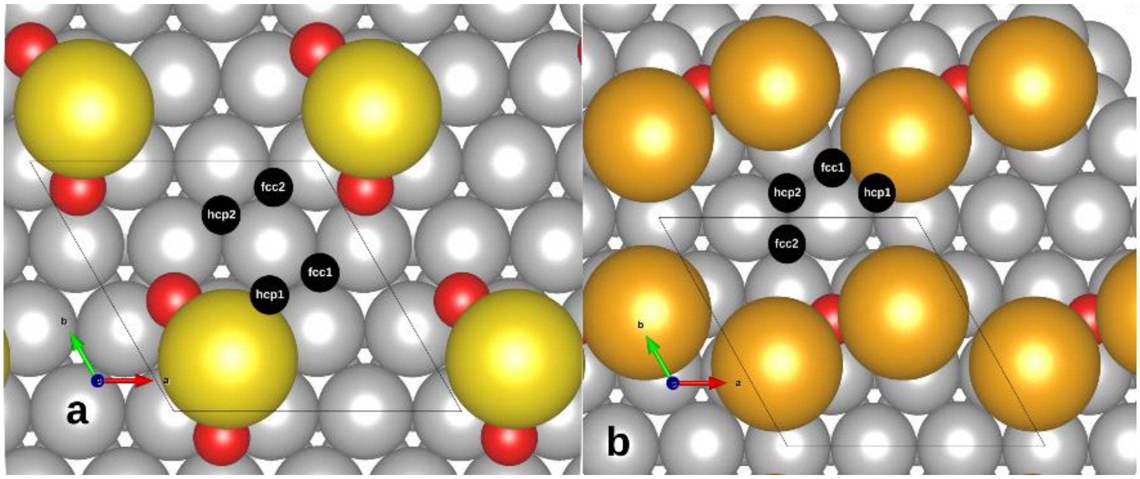


**Figure CM3:** Adsorption sites for $H^*$ and $CO^*$ on a) $PrO_2$- and b) $Na_2O$-covered Co(111) surfaces for an adsorbate/promoter coverage of 0.11 ML. Color codes: Pr: yellow, Na: orange, O: red, Co: gray.

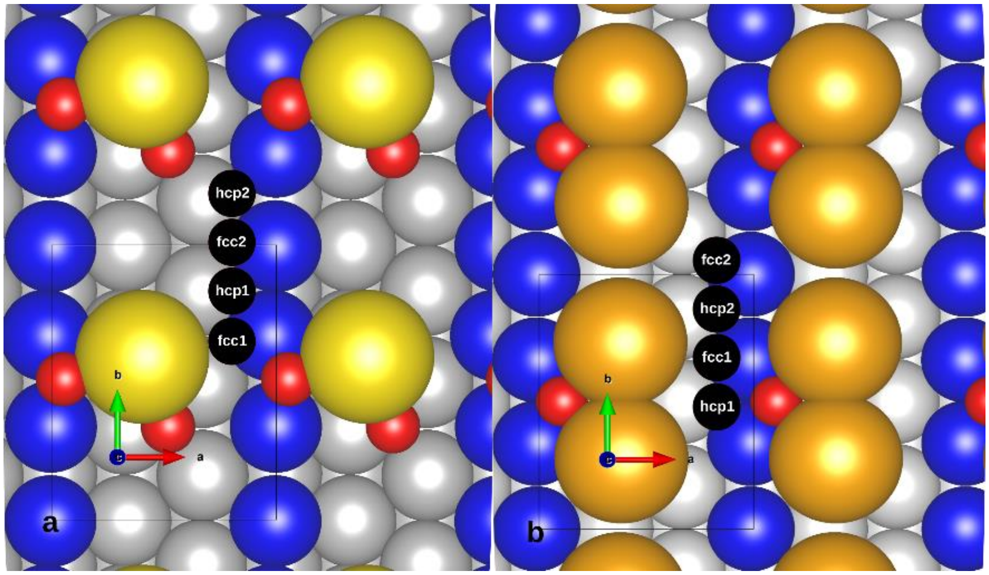


**Figure CM4:** Adsorption sites for $H^*$ and $CO^*$ on a) $PrO_2$- and b) $Na_2O$-covered Co(211) surfaces for an adsorbate/promoter coverage of 0.11 ML. Color codes: Pr: yellow, Na: orange, O: red. For clarity, cobalt atoms at the step-edges are shown in blue, while those on the terrace under the edge as shown in gray.

### *2.1.3. C* adsorption sites on oxide-covered cobalt surface*

As a means to assess the impact of the oxide promoters on the carbophilicity of cobalt, the adsorption of $C^*$ adatoms was investigated. Figure CM5 shows the energetically favored adsorption positions for $C^*$ on Co(111) extended terraces decorated with $PrO_2$ (panel a) and $Na_2O$ (b) units at a total surface coverage of 0.11 ML. As observed, energetically preferred $C^*$ adsorption sites are associated to a direct interaction of carbon with the cations in the oxide promoter units.

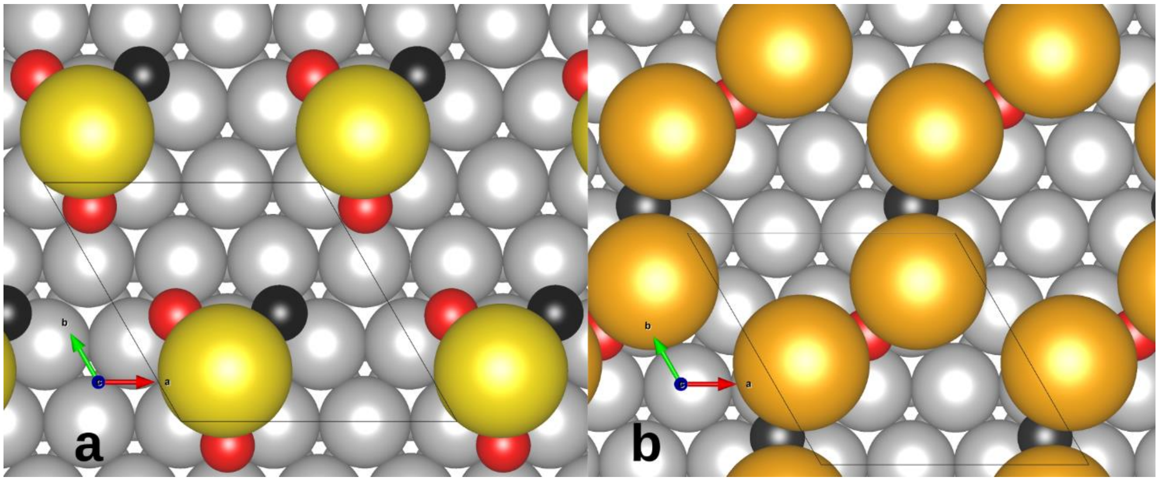


**Figure CM5:** Adsorption sites for atomic C on a) $PrO_2$- and b) $Na_2O$-covered Co(111) surfaces for an adsorbate/promoter coverage of 0.11 ML. Color codes: Pr: yellow, Na: orange, O: red, Co: grey, C: black.

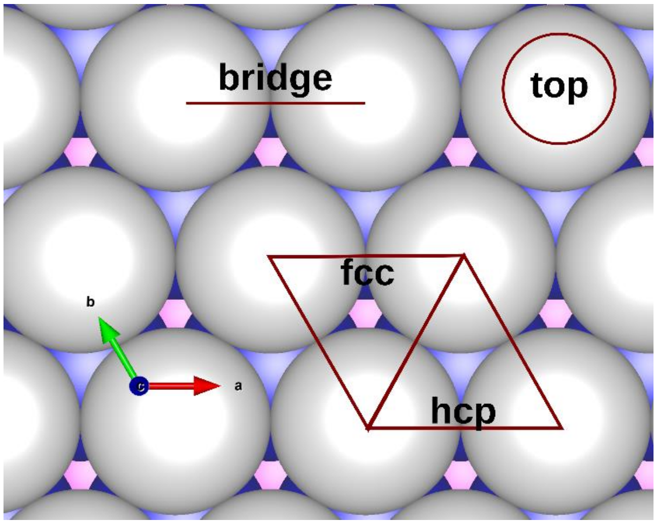


**Figure CM6**: Adsorption sites on *fcc*-cobalt surfaces. Cobalt atoms at different layers are shown in silver (top layer), blue (2nd layer) and purple (3rd layer).

## 3. Supporting Figures

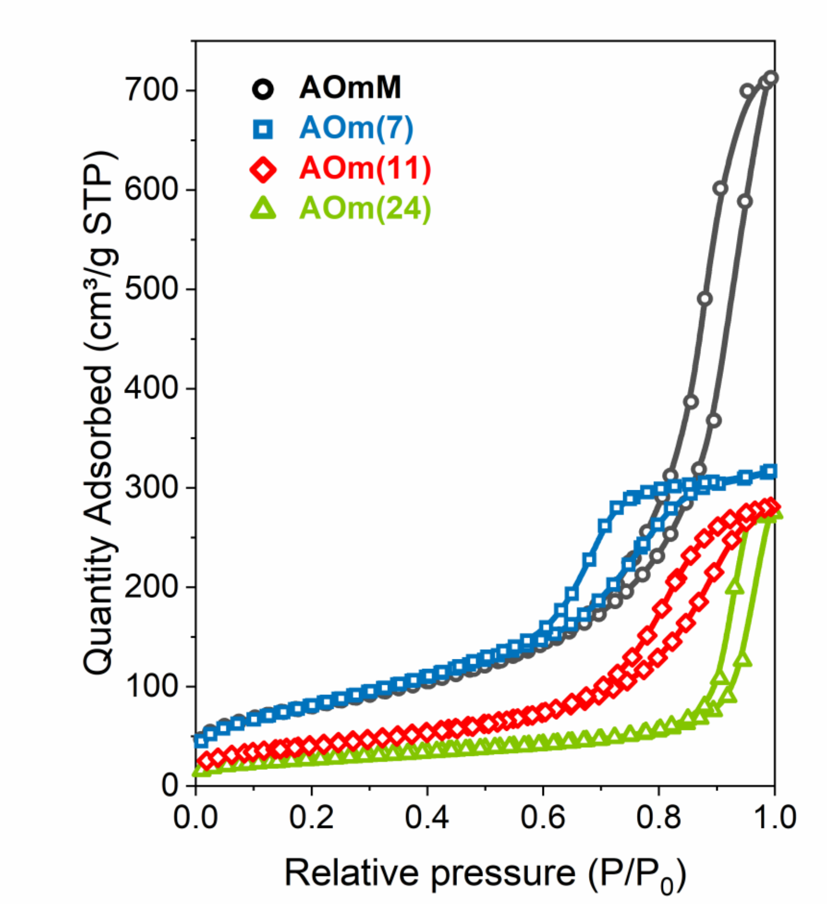


**Figure S1:** $N_2$-physisorption isotherms recorded at 77 K for the series of $\gamma$-$Al_2O_3$ catalyst supports.

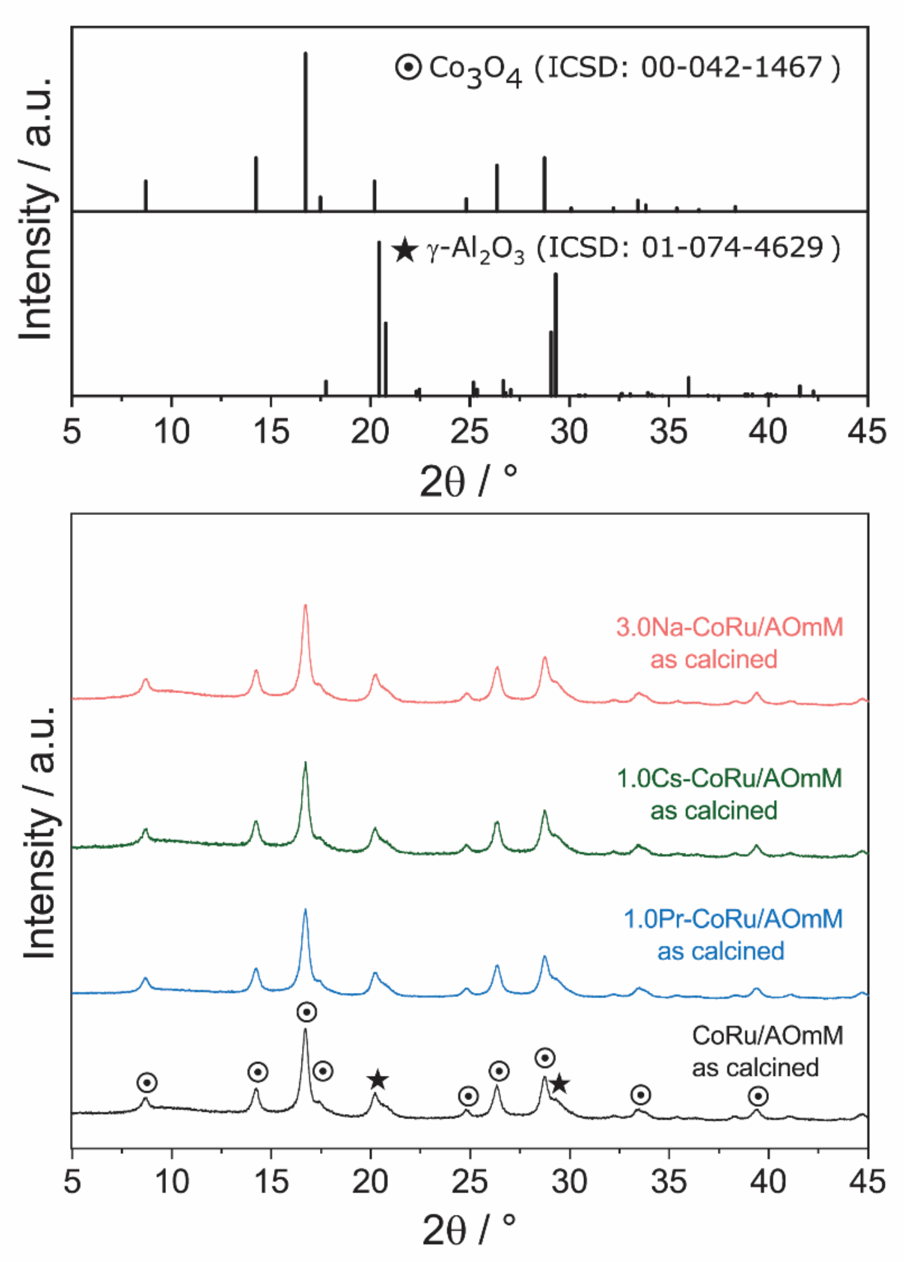


**Figure S2:** Powder X-ray diffraction patterns obtained with Mo-K-α-radiation (17.45 keV) for selected unpromoted and alkali- or lanthanide-promoted CoRu/$Al_2O_3$ catalysts in their calcined state, along with reference patterns from the International Crystal Structure Data Base (ICSD).

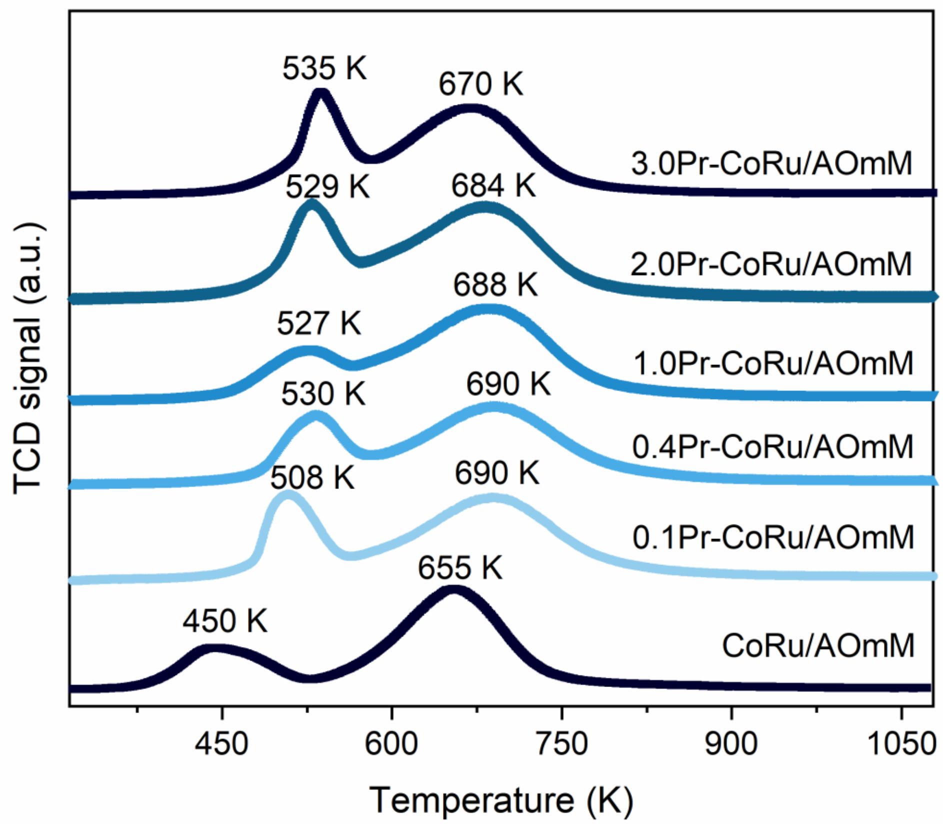


**Figure S3:** Temperature-programmed reduction profiles of Co-based FT catalysts supported on meso-macroporous $\gamma$-$Al_2O_3$ promoted with $PrO_x$ at various Pr surface loadings in the range of 0.1-3.0 $Pr_{at}$ $nm^{-2}$. For reference, the $H_2$-TPR profile for the unpromoted CoRu/AOmM catalyst is also shown.

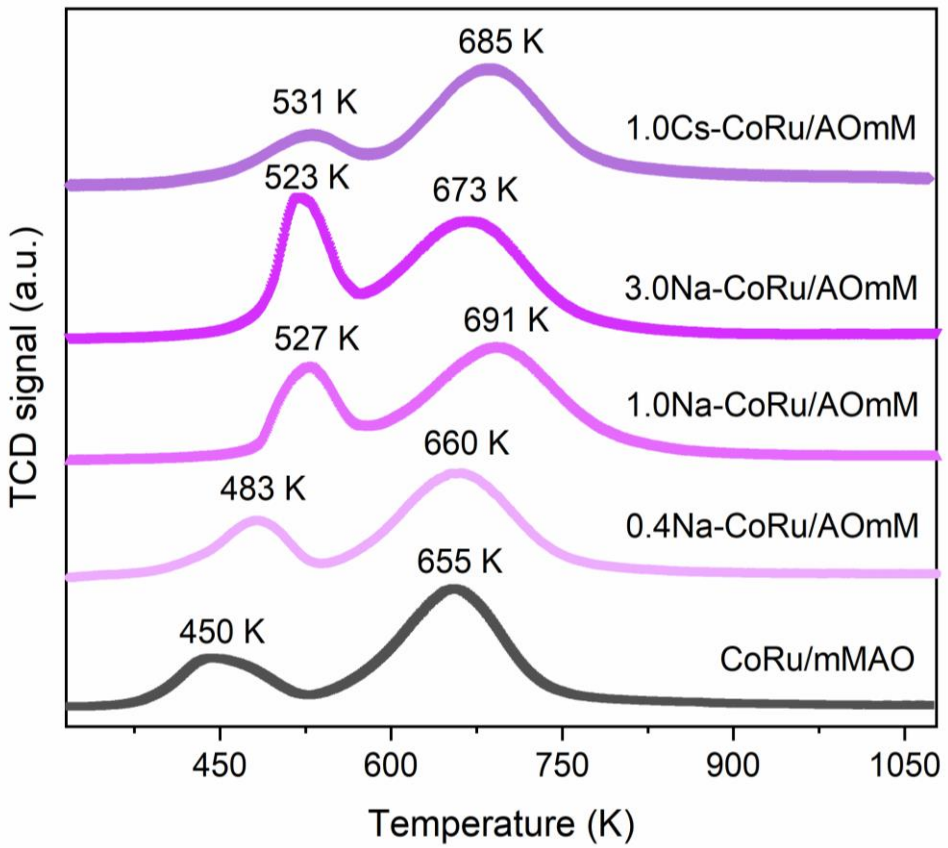


**Figure S4:** Temperature-programmed reduction profiles of Co-based FT catalysts supported on meso-macroporous γ-$Al_2O_3$ promoted with $NaO_x$ or $CsO_x$ at various alkaline metal surface loadings in the range of 0.4-3.0 $Na_{at}$ $nm^{-2}$. For reference, the $H_2$-TPR profile for the unpromoted CoRu/AOmM catalyst is also shown.

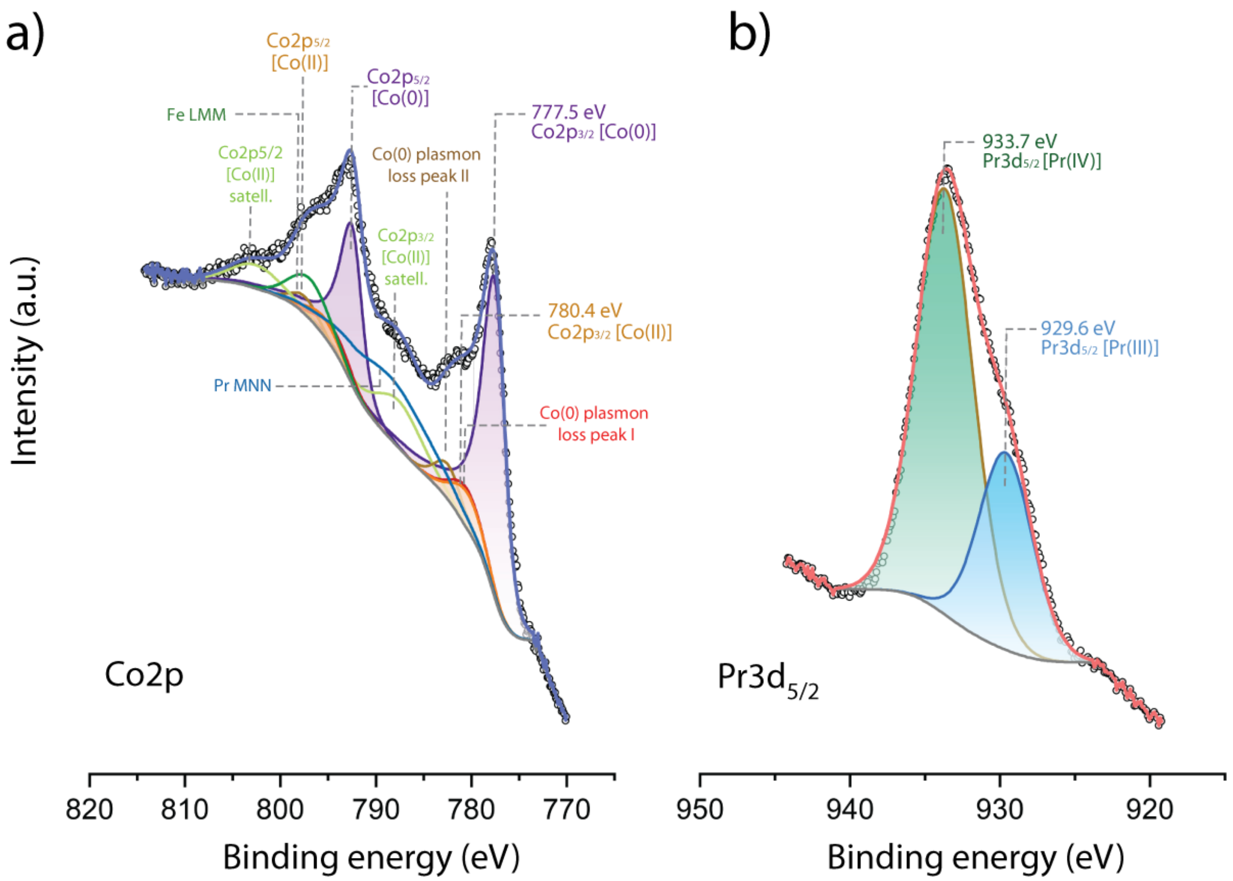


**Figure S5:** X-ray photoelectron spectrum in the Co2p and Pr3d$_{5/2}$ spectral regions for 3.0Pr-CoRu/AOmM after $H_2$ reduction. Deconvolution of the Co2p region, in addition to main Co(0) and Co(II) components, the two metal plasmon loss peaks at 3.0 eV and 5.0 eV, respectively, above the main peak, and the shake-up satellites for Co(II), included in this case contributions from the PrMNN and FeLMM Auger signals, the latter arising from the stainless-steel sample holder applied for the *in situ* catalyst reduction.

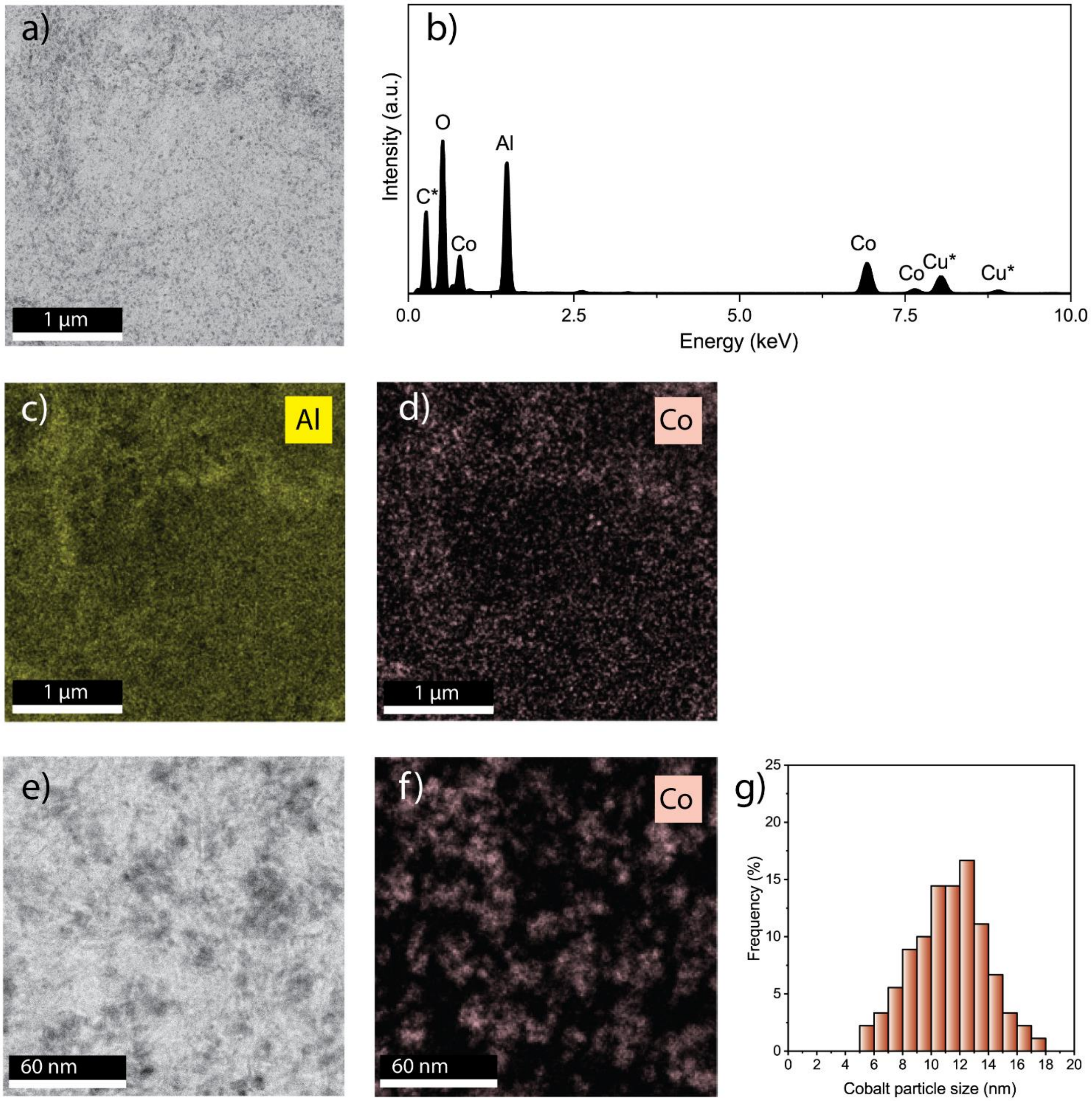


**Figure S6:** Bright field Cs-STEM and EDS microanalysis on ultramicrotomed catalyst cross-sections of CoRu/AOmM. a) representative BF-STEM mesoscale micrograph and; b) the corresponding ED spectrum. Spectral contributions from the embedding carbonaceous resin and the copper grid are marked with asterisks; c,d) EDS compositional maps for the region imaged in (a) for Al and Co, respectively, obtained from the corresponding EDS K-lines; e) representative BF-STEM nanoscale micrograph and; f) the corresponding EDS compositional map for Co; g) cobalt nanoparticle size distribution.

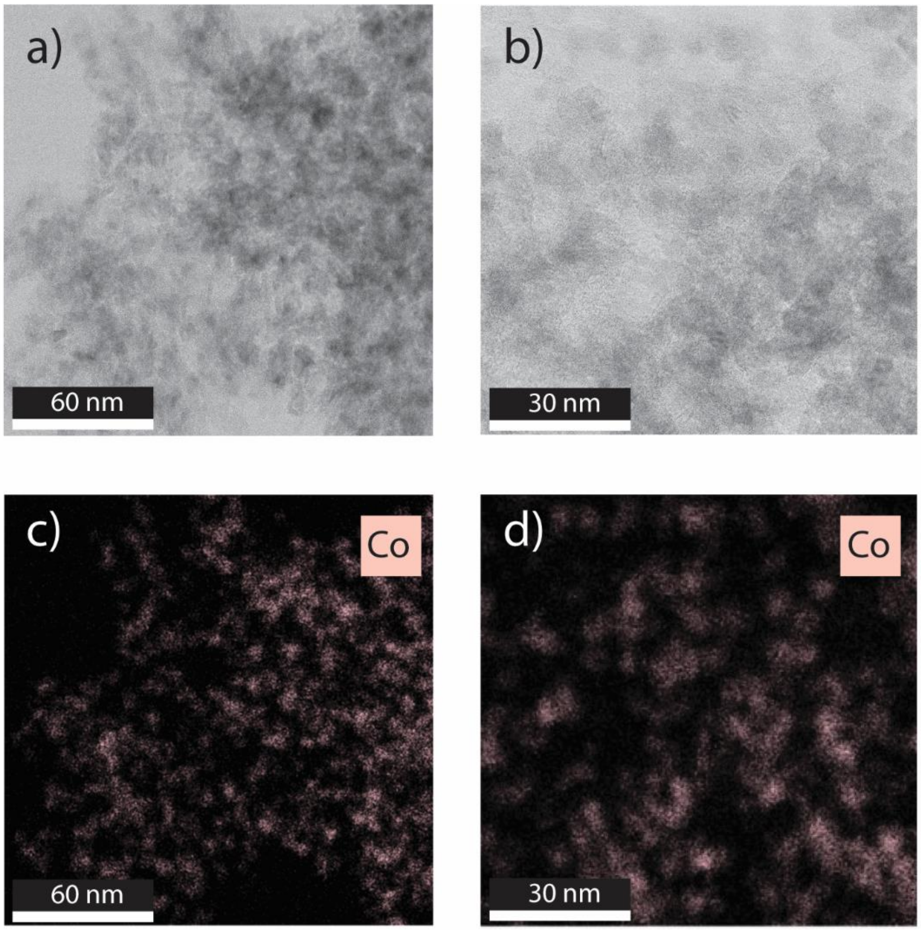


**Figure S7:** Representative bright-field STEM micrographs (a,b) and the corresponding nano-scale cobalt-K EDS compositional maps (c,d) for ultramicrotomed cross-sections of 1.0Pr-CoRu/AOmM after $H_2$-reduction and cobalt surface passivation, showing details on the cobalt nanoparticles.

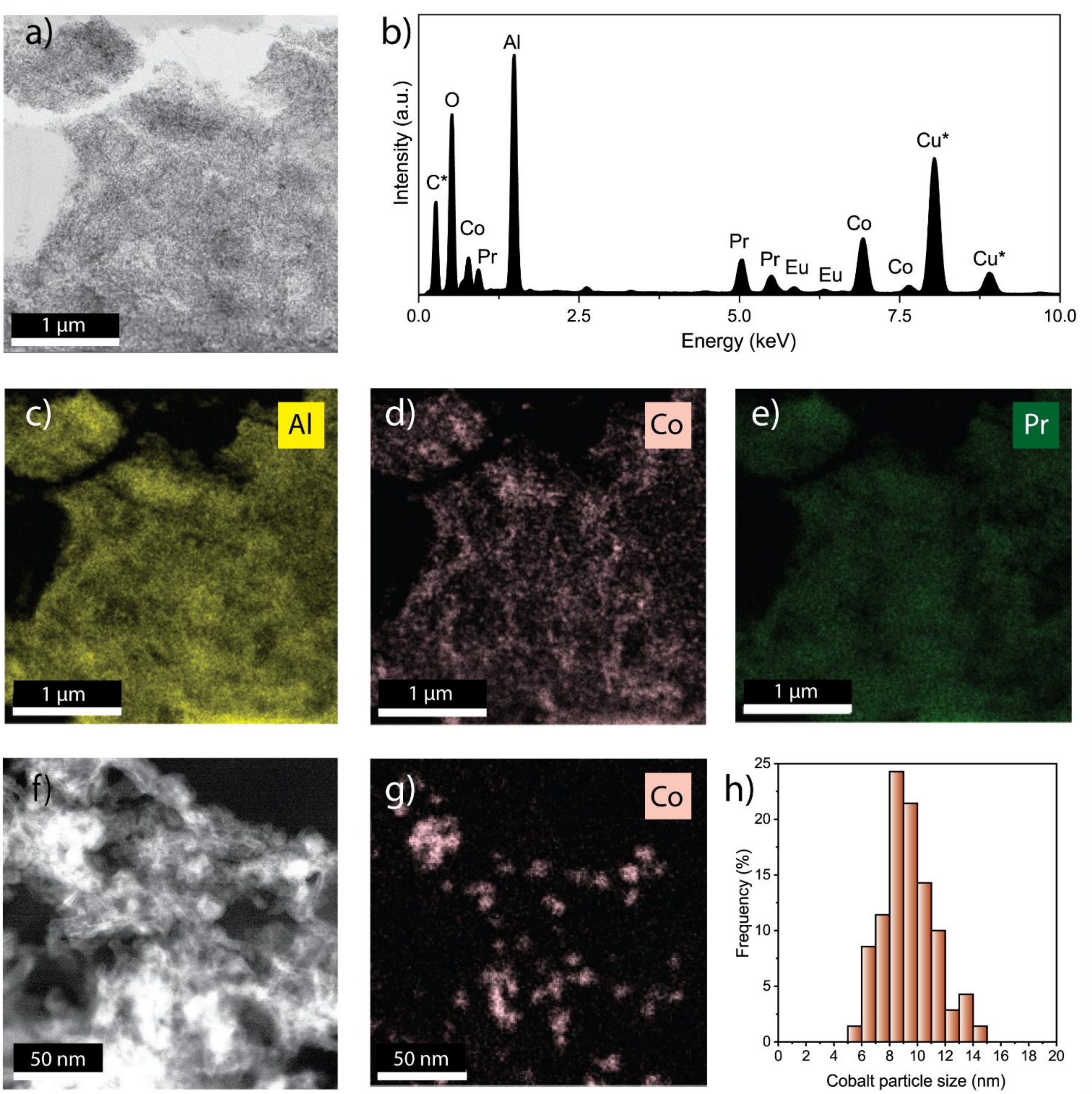


**Figure S8:** Bright field $C_s$-STEM and EDS microanalysis on ultramicrotomed catalyst cross-sections of 3.0Pr-CoRu/AOmM. a) representative BF-STEM mesoscale micrograph and; b) the corresponding ED spectrum. Spectral contributions from the embedding carbonaceous resin and the copper grid are marked with asterisks, Eu was found as a result of its presence as an impurity in the Pr precursor; c,d,e) EDS compositional maps for the region imaged in (a) for Al, Co and Pr, respectively, obtained from the corresponding EDS K-lines; f) representative HAADF-STEM nanoscale micrograph and; g) the corresponding EDS compositional map for Co; h) cobalt nanoparticle size distribution.

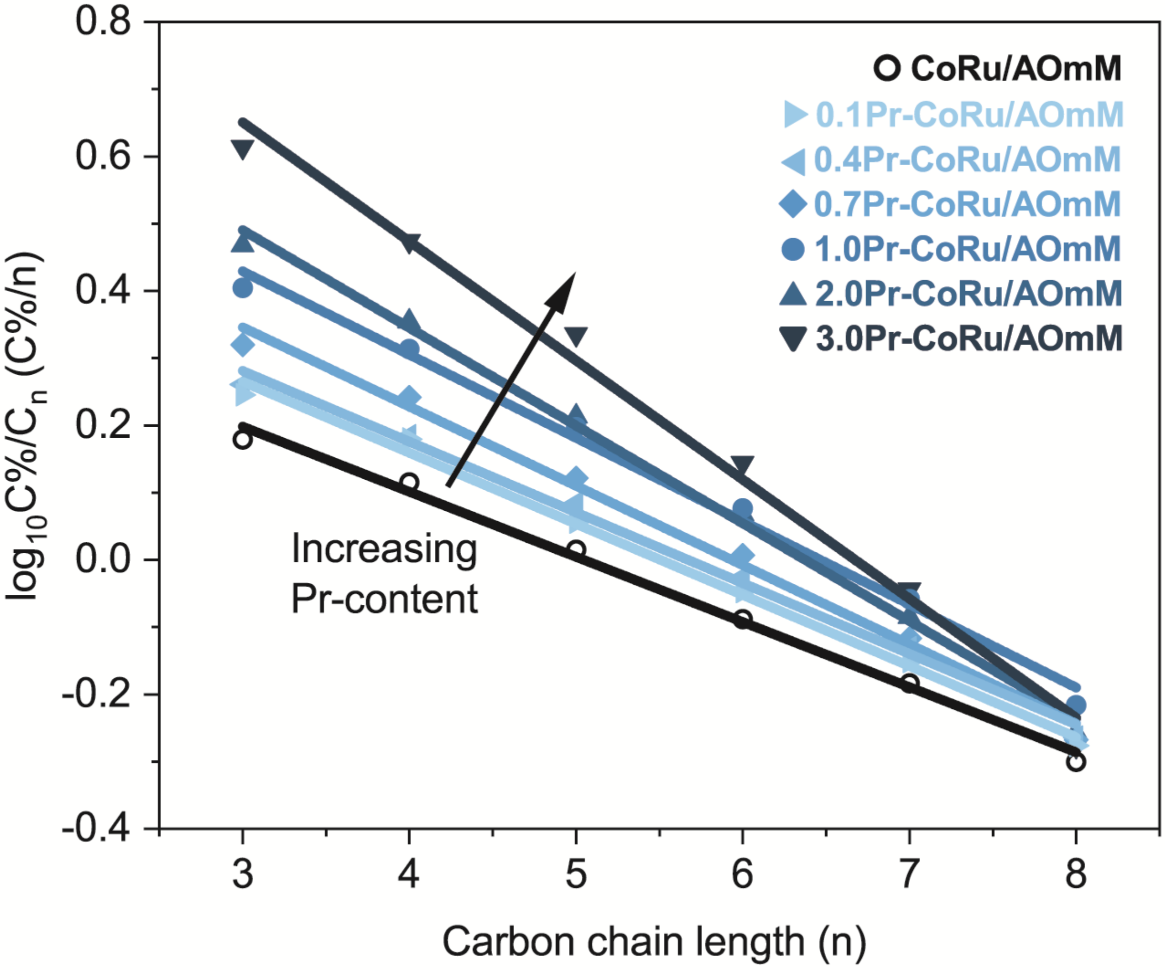


**Figure S9:** Linearized ASF plot in the $C_{3-8}$ hydrocarbon product range derived from online product analysis for cobalt-based FT catalysts supported on a multimodal meso-macroporous γ-$Al_2O_3$, both unpromoted (CoRu/AOmM) and additionally promoted with $PrO_x$ at various Pr surface loadings in the range of 0.1-3.0 $Pr_{at}$ $nm^{-2}$. The arrow in the plot indicates the direction of increasing Pr content. Reaction conditions: T=473 K, P=20 bar, $H_2$/CO=2, CO conversion=20±3%, WSHV=5.5-11.0 $h^{-1}$.

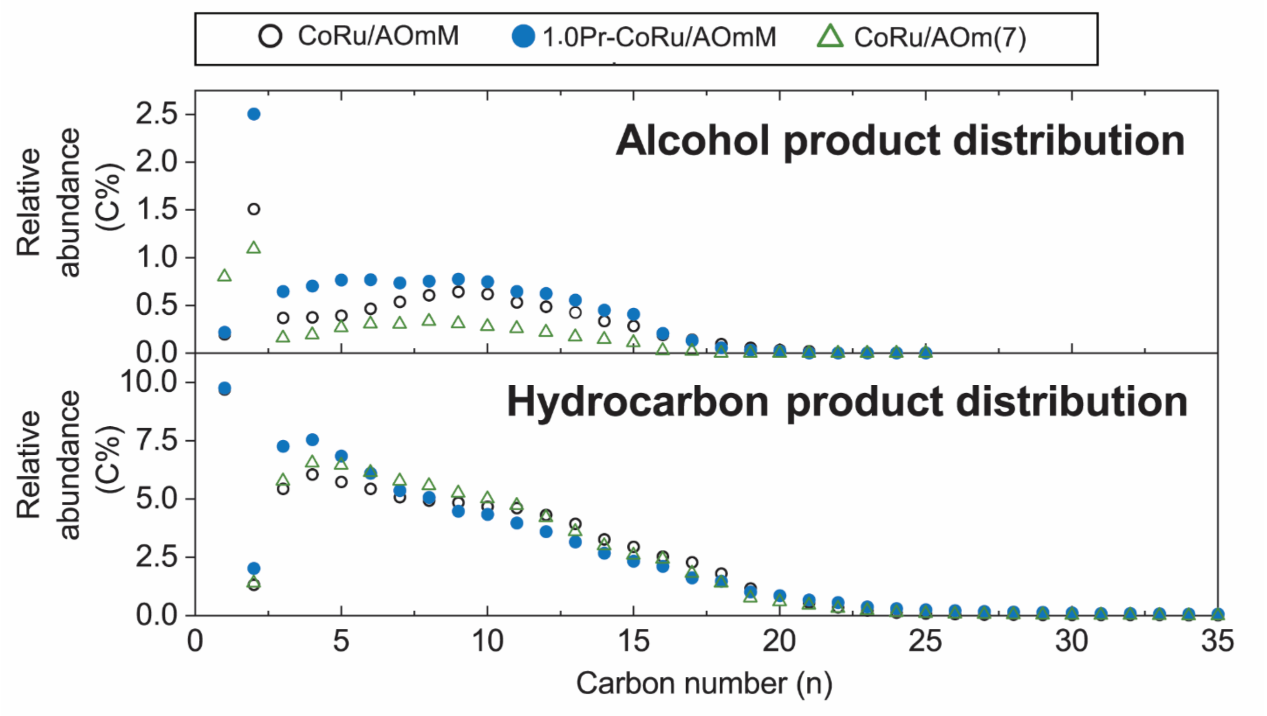


**Figure S10:** Fischer-Tropsch alcohol and hydrocarbon product distributions, based on integrated online+offline gas chromatography analyses, obtained in the pseudo-steady state (ToS~190 h) with selected catalysts.

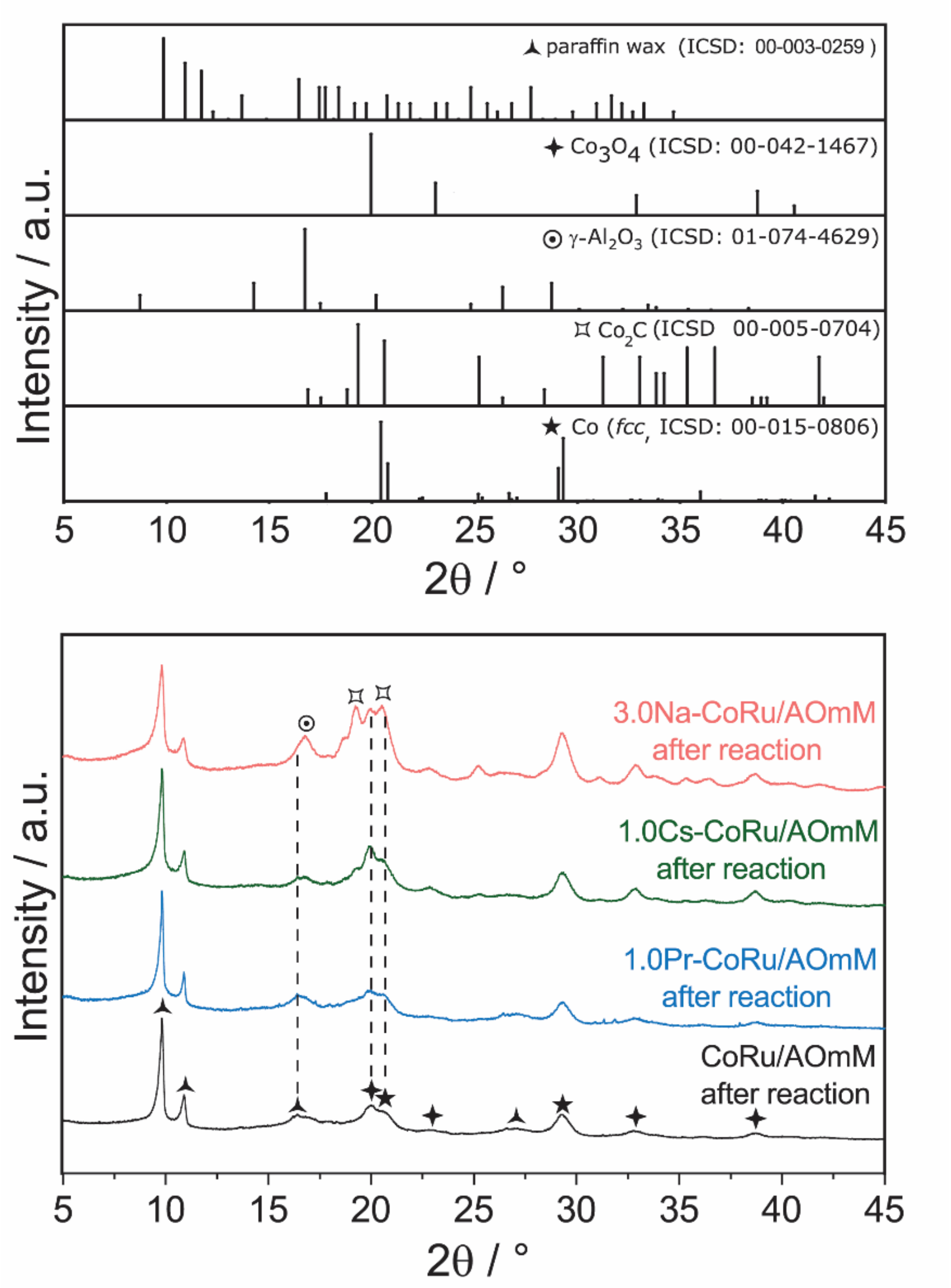


**Figure S11:** Powder X-ray diffraction patterns obtained with Mo-K-α-radiation (17.45 keV) for selected unpromoted and alkali- or lanthanide-promoted CoRu/$Al_2O_3$ catalysts after reaction under Fischer-Tropsch conditions (T=473 K, P=20 bar, $H_2$/CO=2, ToS ≥32 h), along with reference patterns from the International Crystal Structure Data Base (ICSD). After the Fischer-Tropsch test, the *spent* catalyst was separated from the SiC granules applied as diluent of the packed bed prior to XRD measurement by attraction with a Nd magnet. In all cases, the diffraction patterns present signals ascribed to crystallized solid waxes retained within the porosity of the catalysts. Weak diffraction contributions corresponding to $Co_3O_4$ are likely associated to partial cobalt reoxidation during the offloading of the catalyst from the tubular reactor which could not be performed under exclusion of air.

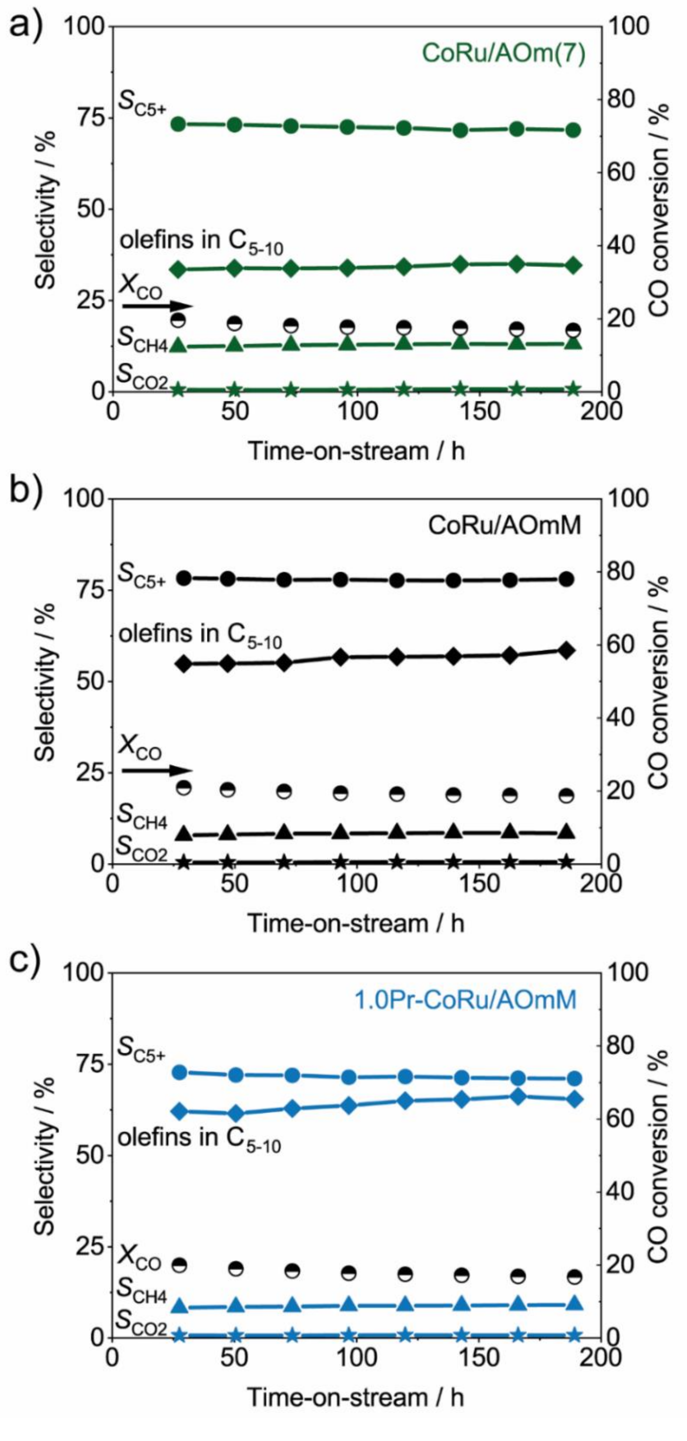


**Figure S12:** CO conversion (*X*), product selectivity (*S*) to $CH_4$, $CO_2$ and $C_{5+}$ hydrocarbons and $C_5$-$C_{10}$ olefin abundance within the $C_{5-10}$ hydrocarbon products for (a) CoRu/AOm(7), (b) CoRu/AOM, and (c) 1.0Pr-CoRu/AOmM, during aprox. 190 h on-stream in the pseudo-steady state. Reaction conditions: T=473 K, P= 20 bar, $H_2$/CO=2.0. Products formed in the first 24 h on-stream (transient period) were discarded for the purpose of offline liquid and solid hydrocarbon product analysis corresponding exclusively to the pseudo-steady operation state.

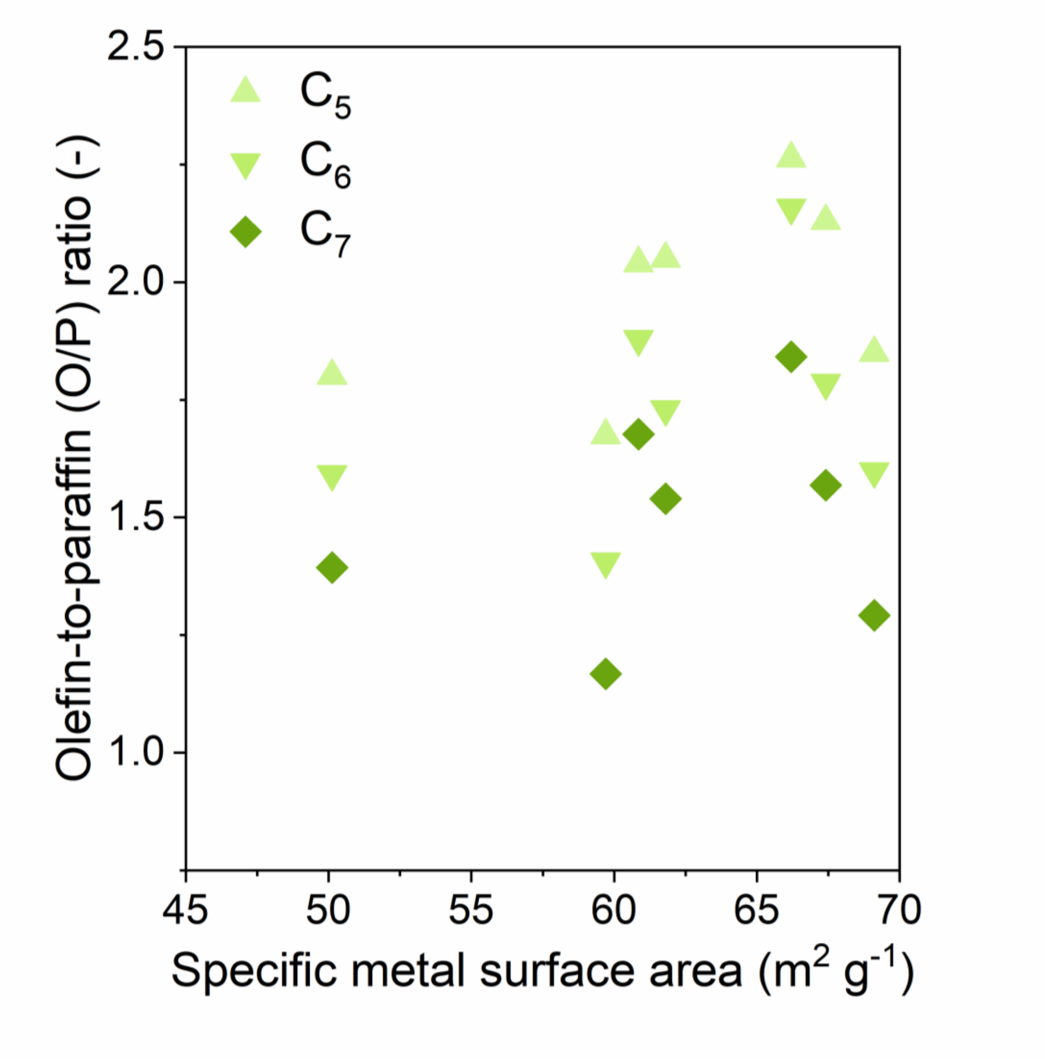


**Figure S13:** Molar olefin-to-paraffin (O/P) ratio for $C_5$, $C_6$ and $C_7$ hydrocarbon products obtained with various cobalt-based FT catalysts supported on a multimodal meso-macroporous γ-$Al_2O_3$ support and optionally promoted with $PrO_x$ at different Pr surface loadings as a function of the specific metal surface area (per unit mass of catalyst) quantified by $H_2$ chemisorption following catalyst reduction. Reaction conditions: T=473 K, P=20 bar, $H_2$/CO=2, CO conversion=20±5%, WHSV=5.5-33.0 $h^{-1}$.

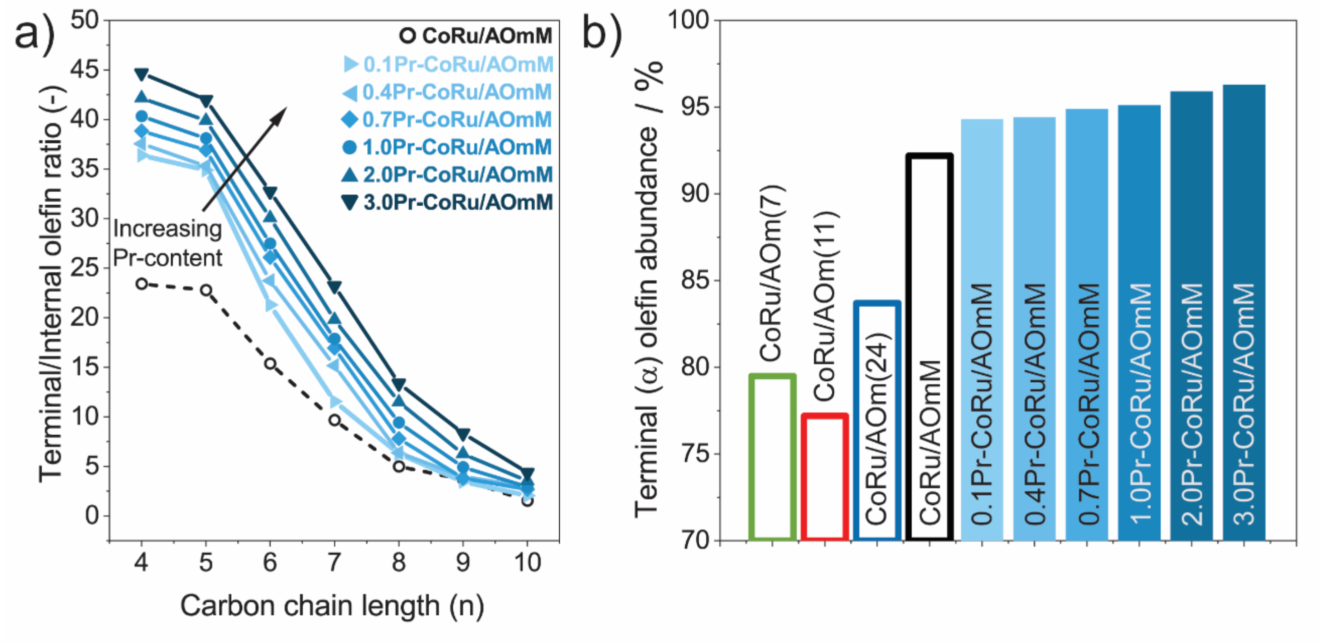


**Figure S14:** Evolution of a) the terminal-to-internal olefin molar ratio for $C_{10-}$ olefin products, and b) molar abundance of terminal (α) olefins within the $C_{5-10}$ olefin fraction, for products obtained with Co-based FT catalysts supported on a multimodal meso-macroporous γ-$Al_2O_3$ promoted with $PrO_x$ at increasing surface loadings in the range of 0.1-3.0 $Pr_{at}$ $nm^{-2}$ . Data for the corresponding unpromoted CoRu/AOmM catalyst is also shown for reference. For reference, panel (b) includes data also for CoRu/$Al_2O_3$ catalysts supported on unimodally mesoporous alumina carriers with various average pore dimeter *d* (nm) (CoRu/AOm(*d*)). Reaction conditions: T=473 K, P=20 bar, $H_2$/CO=2, WHSV=11.0-12.1 $h^{-1}$.

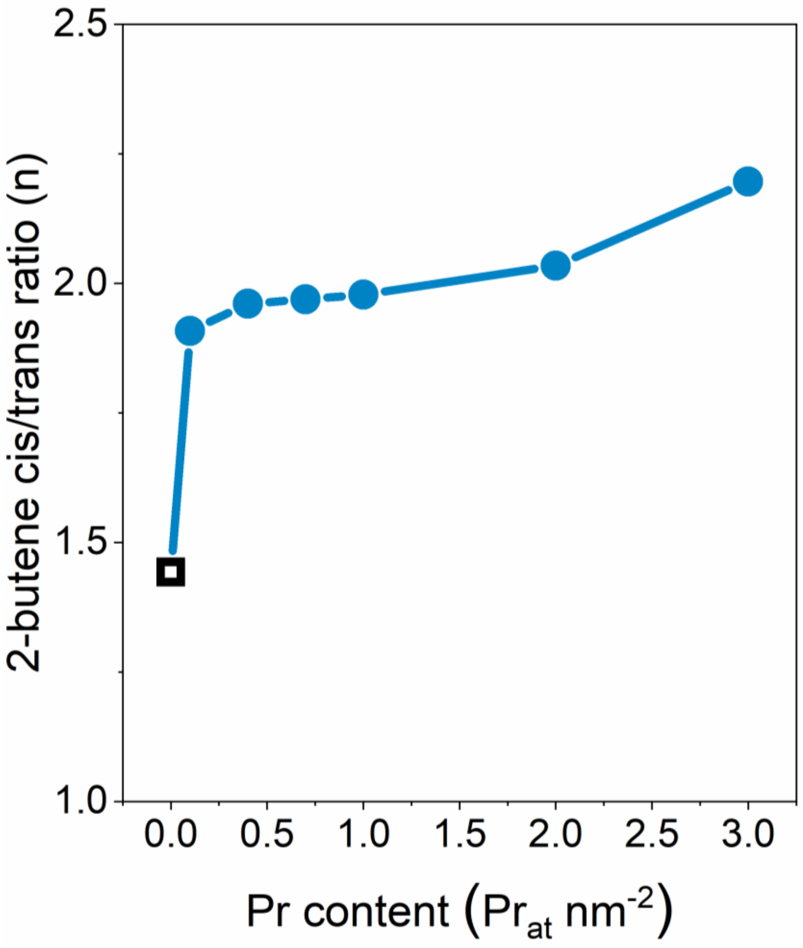


**Figure S15:** 2-butene cis/trans isomer molar ratio in the FT products obtained with cobalt-based FT catalysts supported on a meso-macroporous γ-$Al_2O_3$ promoted with $PrO_x$ as a function of the Pr content. Data for the corresponding unpromoted CoRu catalyst is also shown for reference (open symbol). Reaction conditions: T=473 K, P=20 bar, $H_2$/CO=2, WHSV=11.0 $h^{-1}$.

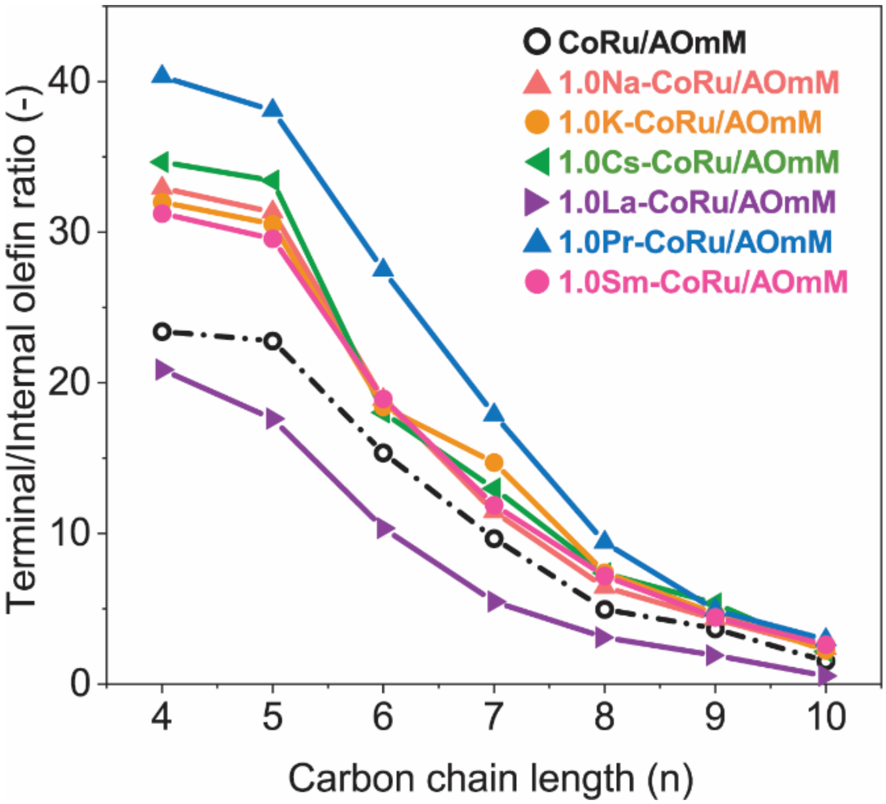


**Figure S16:** Evolution of the terminal-to-internal olefin molar ratio for $C_{10-}$ olefin products obtained with various cobalt-based FT catalysts supported on a multimodal meso-macroporou $\gamma$-$Al_2O_3$ support and promoted with different Lewis basic alkali and lanthanide oxides at a surface-specific promoter content of $1.0M_{at}$ $nm^{-2}$. Data for the corresponding unpromoted CoRu catalyst is also shown for reference (dotted line, open symbols). Reaction conditions: T=473 K, P=20 bar, $H_2$/CO=2, WHSV=11.0 $h^{-1}$.

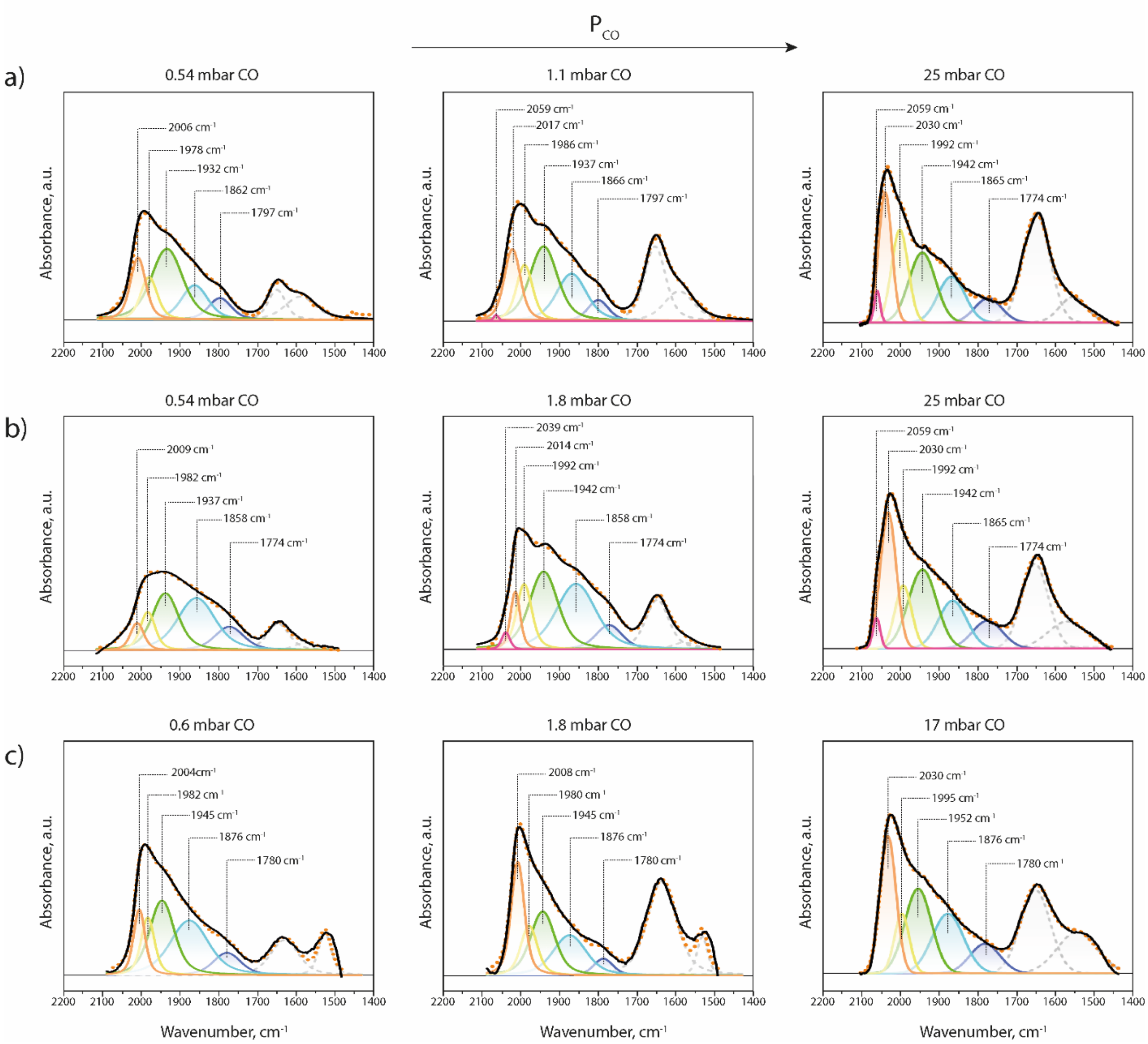


**Figure S17:** Deconvolution of CO-FTIR spectra in the υ(CO) region, recorded at 298 K after increasing CO dosages (given on the individual plots) on the *in situ* reduced a) CoRu/AOmM, b) 1.0Pr-CoRu/AOmM and c) 3.0Pr-CoRu/AOmM catalysts. Color codes for the different peak contributions/species apply to the entire figure.

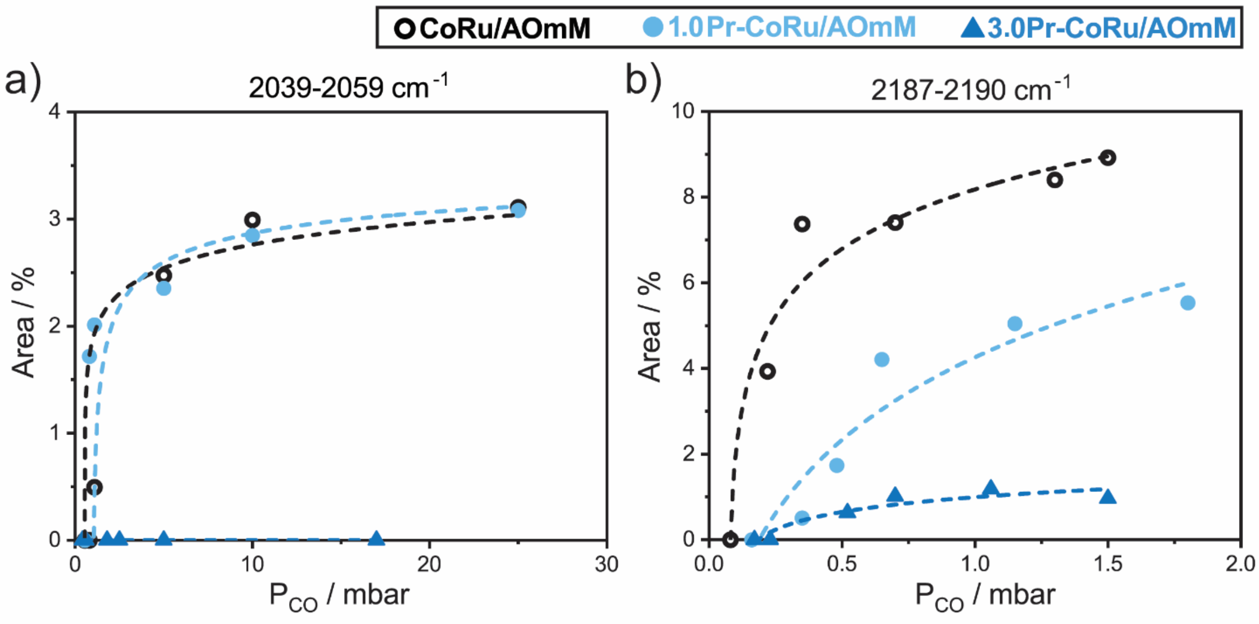


**Figure S18:** Area contribution as a function of the CO dosage pressure for FTIR bands peaking at a) 2039-2059 $cm^{-1}$ after dosing of CO at 298 K, and b) 2187-2190 $cm^{-1}$ after dosing of CO at 110 K, on the *in situ* reduced CoRu/AOmM, 1.0Pr-CoRu/AOmM and 3.0Pr-CoRu/AOmM catalysts, illustrating the dependence of the band intensity with the CO dosage (for any given sample) as well as with the Pr surface content (across different samples).

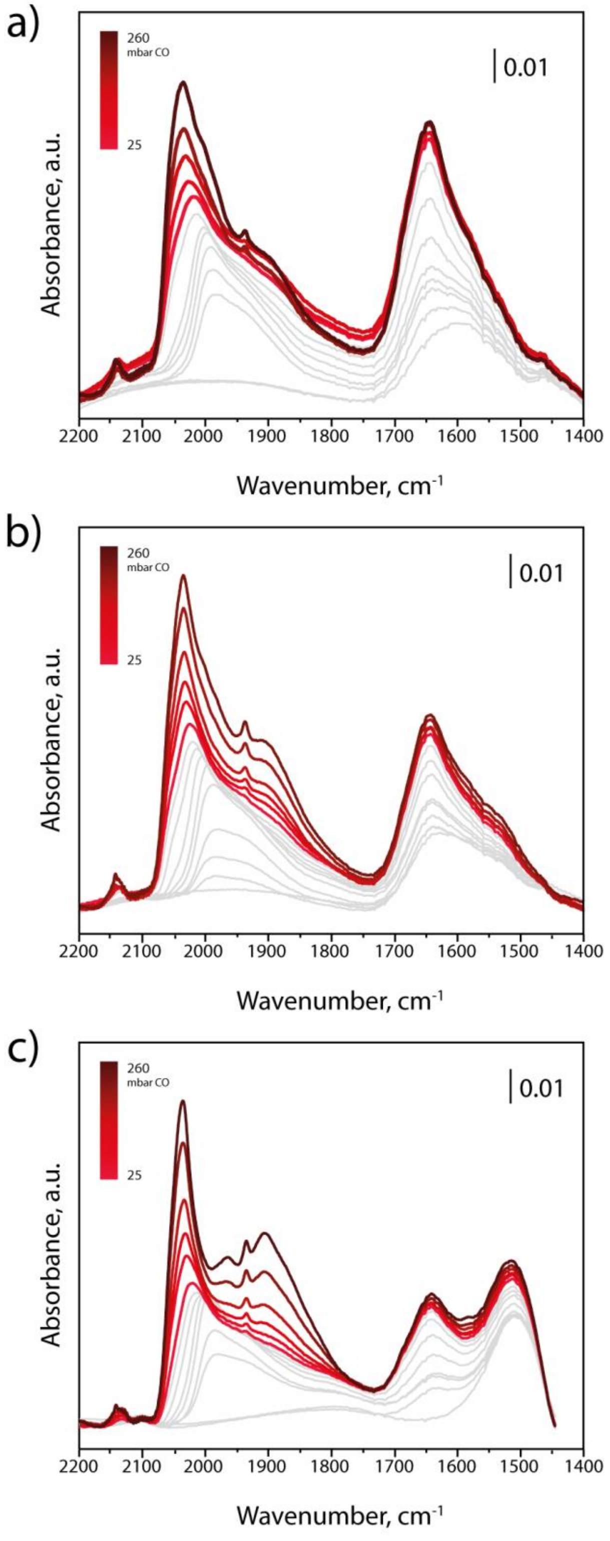


**Figure S19:** FTIR spectra in the υ(CO) region recorded at 298 K after dosing increasing CO partial pressures (25-260 mbar) on the *in situ* reduced a) CoRu/AOmM, b) 1.0Pr-CoRu/AOmM, and c) 3.0Pr-CoRu/AOmM catalysts. Gray traces show spectra recorded at lower CO dosages.

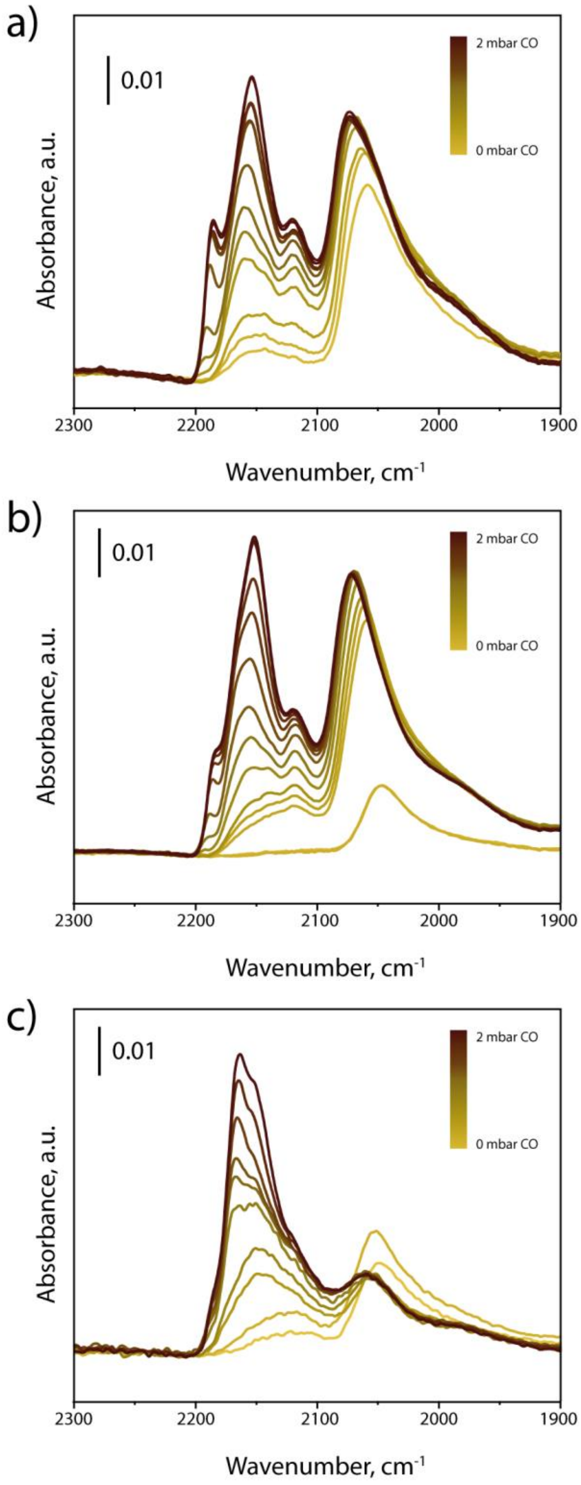


**Figure S20:** FTIR spectra in the υ(CO) region recorded at 110 K after dosing increasing CO partial pressures (0-2 mbar) on the *in situ* reduced a) CoRu/AOmM, b) 1.0Pr-CoRu/AOmM, and c) 3.0Pr-CoRu/AOmM catalysts.

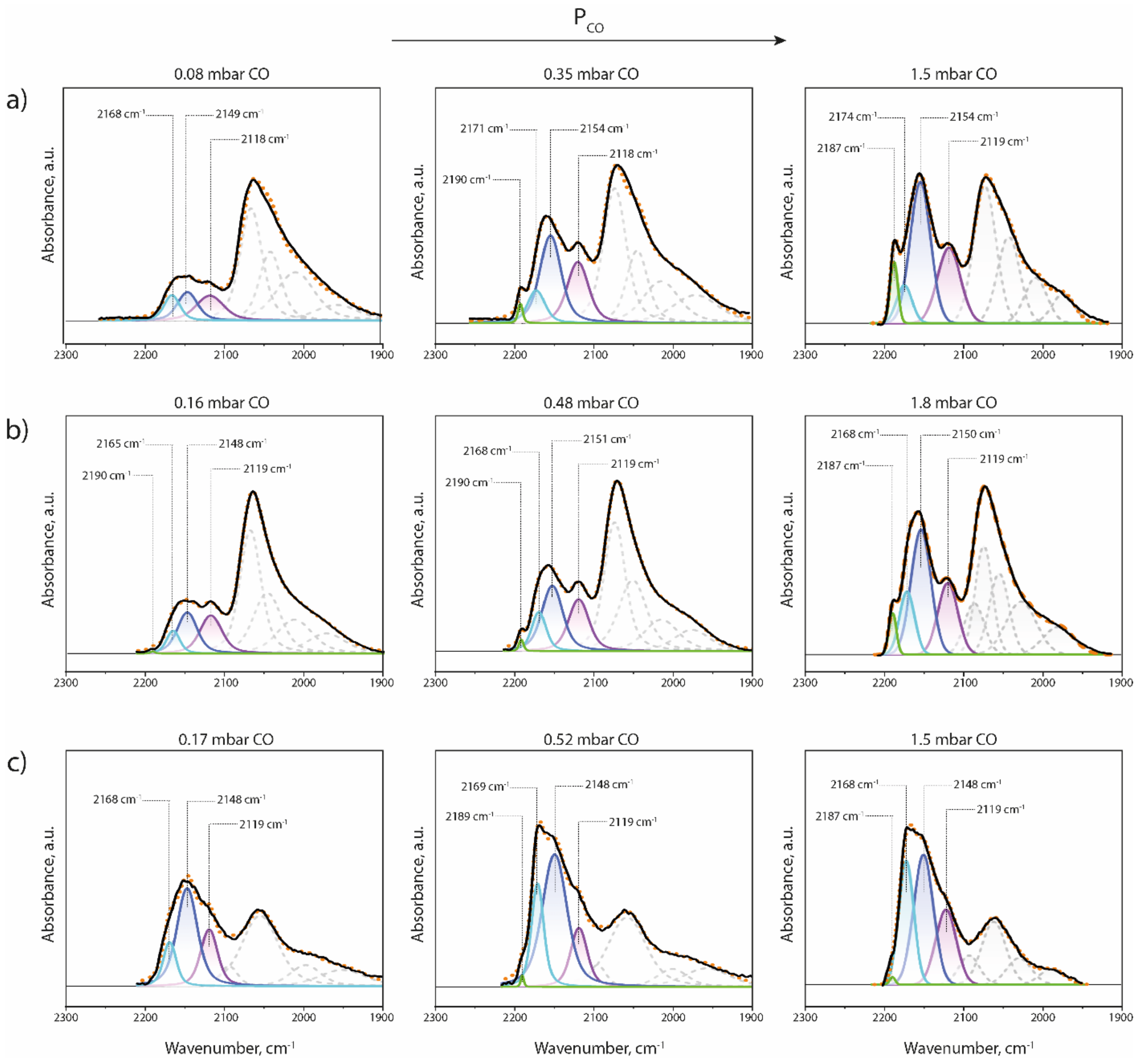


**Figure S21:** Deconvolution of CO-FTIR spectra in the υ(CO) region, recorded at 110 K after increasing CO dosages (given on the individual plots) on the *in situ* reduced a) CoRu/AOmM, b) 1.0Pr-CoRu/AOmM and c) 3.0Pr-CoRu/AOmM catalysts. Color codes for the different peak contributions/species apply to the entire figure. Contributions indicated by dashed peaks correspond to CO adsorbed on the metal nanoparticles and have therefore not been considered as they are better resolved in our experiments at 298 K (see Figure S17).

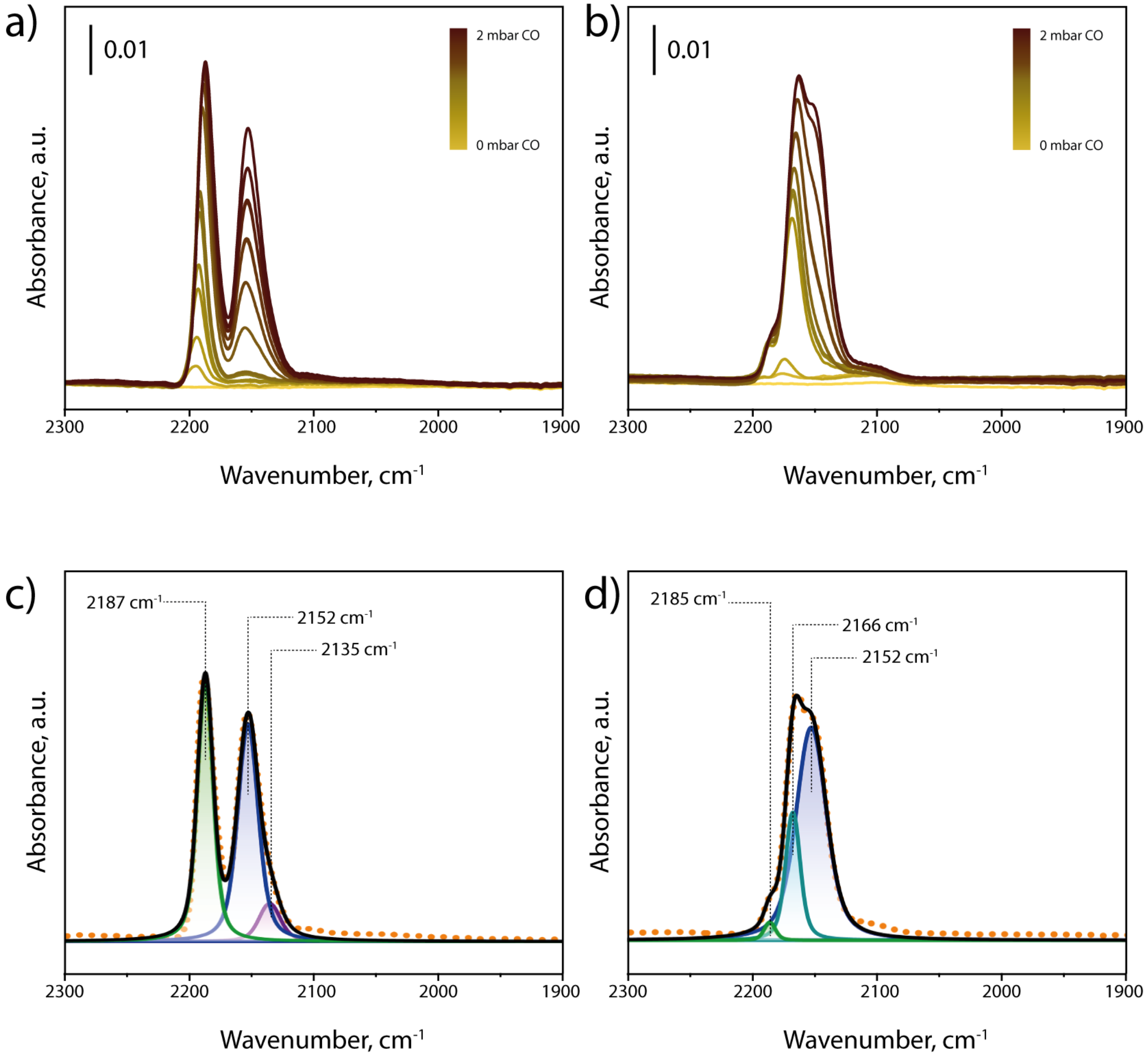


**Figure S22:** FTIR spectra in the υ(CO) region recorded at 110 K after dosing increasing CO partial pressures (0-2 mbar) (a,c) and deconvolution of the spectrum recorded at maximum coverage (2 mbar CO) (b,d) for cobalt-free AOmM (a,b) and 3.0Pr-AOmM (c,d).

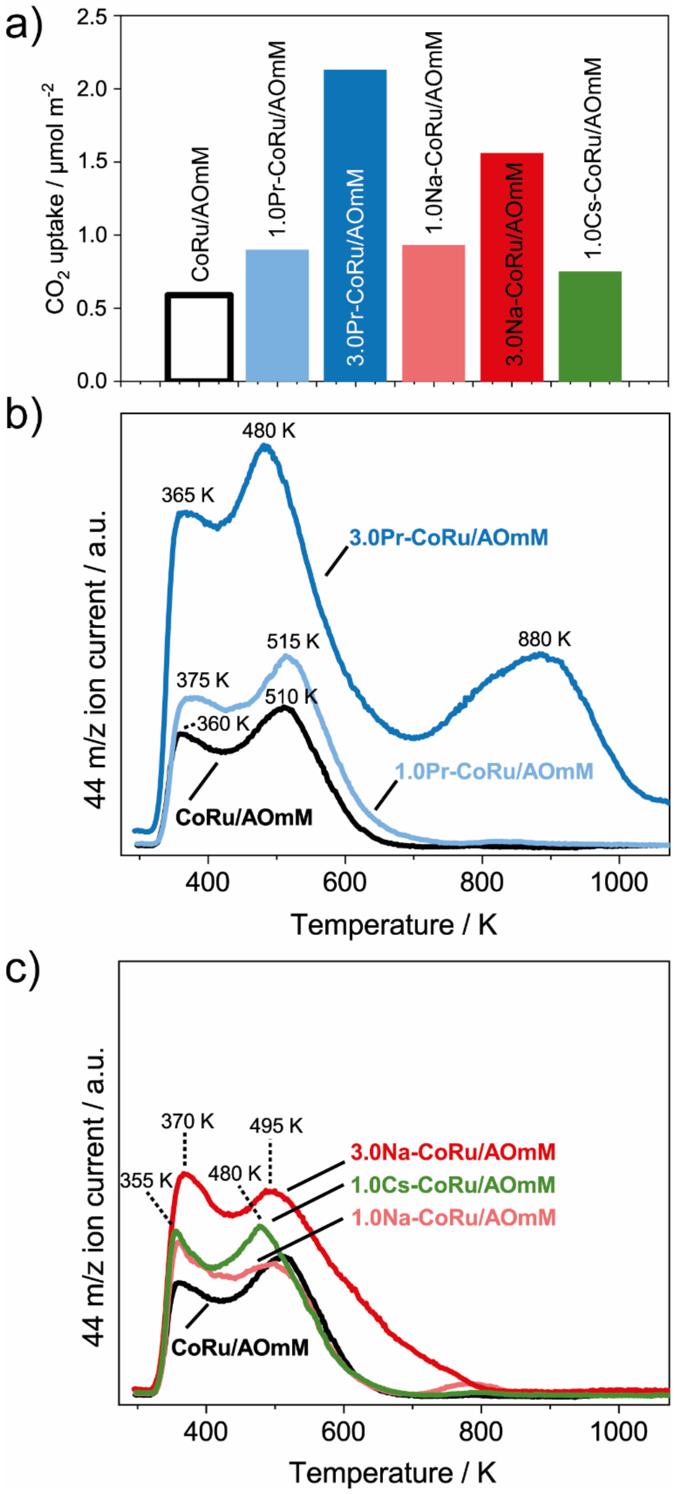


**Figure S23:** a) Surface-normalized saturation (RT) $CO_2$ uptakes and b,c) $CO_2$ temperature-programmed desorption profiles for unpromoted as well as $PrO_x$-promoted (a,b) and alkali oxide-promoted (a,c) CoRu/AOmM catalysts.

Changes in the surface basicity upon promotion with either lanthanide ($PrO_x$) or alkali ($NaO_x$, $CsO_x$) oxides was assessed by means of $CO_2$-TPD on selected CoRu/AOmM after in situ reduction, i.e. in their FT-active state. Figure S23a summarizes the total surface-specific $CO_2$ uptakes. As observed, increments (up to 57%) in $CO_2$ uptake, relative to that for the unpromoted CoRu/AOmM catalyst (0.59 µmol $CO_2$ $m^{-2}$) were observed upon the incorporation of any of the basic promoters at a surface content of 1.0 $M_{at}$ $nm^{-2}$. Figures S23b and c show the corresponding temperature-resolved $CO_2$ desorption profiles. The promoter-free CoRu/AOmM shows two major desorption events peaking at 360 K and 510 K, respectively, which are therefore associated to the desorption of $CO_2$ from those basic sites existing on the γ-$Al_2O_3$ carrier. Hence, the addition of Pr, Na and Cs as promoter at a surface content of 1.0 $M_{at}$ $nm^{-2}$ increased slightly the density of surface basic centers, but it barely modified the desorption pattern, with only a slight reduction in the desorption temperature for the ca. 500 K $CO_2$ desorption event (by up to 20 K) being registered in the case of alkali promoters. These results suggest that, at this surface loading, promoters mostly block surface acid sites (as observed with CO-FTIR, main text) but do not enhance surface basicity to a remarkable extent. Significantly higher $CO_2$ uptakes of 2.13 and 1.56 µmol $CO_2$ $m^{-2}$ (up to more than threefold relative to the unpromoted catalyst) were observed when the content of $PrO_x$ and $NaO_x$ promoters, respectively, was increased to 3.0 $M_{at}$ $nm^{-2}$, testifying for a noticeable increase in the number of surface basic centers. Regarding the strength of these new centers, the lanthanide and alkali promoters showed a different behavior. In the case of 3.0Na-CoRu/AOmM, next to the increment in desorption bands at temperatures below 600 K, corresponding to relatively weak basic sites as those found on the surface of the unmodified alumina carrier, a tail in the desorption trace extending up to 800 K denoted the development of new types of Lewis basic centers, of higher strength than those existing on γ-$Al_2O_3$. However, in the case of $PrO_x$, next to a significant intensity enhancement in the two desorption peaks below 600 K, a prominent new desorption band was detected centered at 880 K, indicating the development of a significant surface density of comparatively stronger basic sites. The latter band is characteristic for $CO_2$ desorption from bulk lanthanide oxides such as $La_2O_3$.[16-17] This suggests the development of relatively stable surface lanthanide carbonate species upon interaction of the $CO_2$ adsorbate with lattice oxygen species of enhanced basicity in extended lanthanide oxide domains developed on the γ-$Al_2O_3$ surface at higher $PrO_x$ surface loadings. Overall, these results are consistent with, and complementary to, those retrieved on surface acidity using CO-FTIR at cryogenic temperature, and indicate that, at moderate surface contents, i.e. 1.0 $M_{at}$ $nm^{-2}$, lanthanide and alkali oxides mostly act as promoters on the Co surface and block the most acid centers on the alumina carrier, while higher surface contents of 3.0 $M_{at}$ $nm^{-2}$, complete the inhibition of the strongest alumina Lewis acidity and additionally lead to the development of new and stronger basic sites on the catalyst surface.

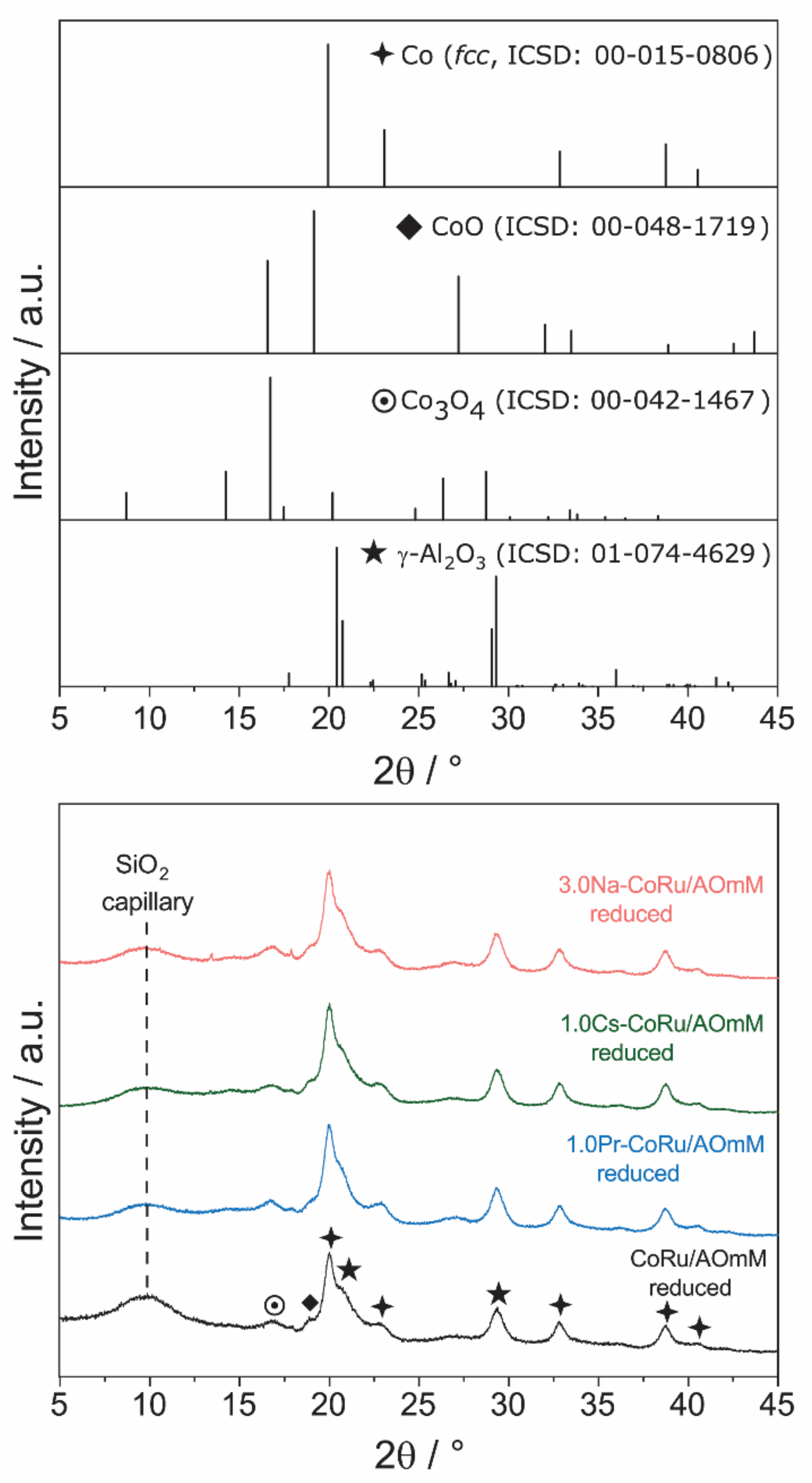


**Figure S24:** Powder X-ray diffraction patterns obtained with Mo-K-α-radiation (17.45 keV) for selected unpromoted and alkali- or lanthanide-promoted CoRu/$Al_2O_3$ catalysts after reduction in $H_2$ flow at 673 K, along with reference patterns from the International Crystal Structure Data Base (ICSD).

## 4. Supporting Tables

**Table S1:** Textural properties of γ-$Al_2O_3$ support materials.

| Support | BET[a] [$m^2\ g^{-1}$] | mesoPV[b] [$cm^3\ g^{-1}$] | macroPV[c] [$cm^3\ g^{-1}$] | *PD*[d] [nm] |
|---|---|---|---|---|
| AOm(7) | 300 | 0.50 | - | 6.8 |
| AOm(11) | 149 | 0.44 | - | 11.2 |
| AOm(24) | 96 | 0.42 | 0.05 | 24.3 (*sh* 49.6) |
| AOmM | 288 | 1.11 | 0.46 | 8.8, 37, $1.03 \cdot 10^3$ |

[a] BET specific surface area determined by $N_2$-physisorption. [b] Mesopore volume as determined by the BJH formalism from the corresponding $N_2$ adsorption isotherms. [c] Macropore volume as determined by integration of the cumulative intrusion volume for pore diameters in the range of 50-$10^4$ nm from the corresponding Hg intrusion results. [d] Major pore diameter modes as determined by the peaking position of maxima in the differential Hg intrusion pore size distribution (see Figure 1 in the main text).

**Table S2:** Bulk-EDS promoter loadings for oxide-promoted CoRu/γ-$Al_2O_3$ catalysts.

| Catalyst | M | $\delta_{M,nom}$[a] [$M_{at}$ $nm^{-2}$] | $M_{exp}$[b] [wt%] | $\delta_{M,exp}$[c] [$M_{at}$ $nm^{-2}$] |
|---|---|---|---|---|
| 0.4Na-CoRu/AOmM | Na | 0.4 | 0.3 | 0.4 |
| 1.0Na-CoRu/AOmM | Na | 1.0 | 0.9 | 1.2 |
| 3.0Na-CoRu/AOmM | Na | 3.0 | 2.1 | 3.0 |
| 1.0K-CoRu/AOmM | K | 1.0 | 1.6 | 1.2 |
| 1.0Cs-CoRu/AOmM | Cs | 1.0 | 4.9 | 1.1 |
| 1.0La-CoRu/AOmM | La | 1.0 | 5.0 | 1.1 |
| 0.1Pr-CoRu/AOmM | Pr | 0.1 | 0.5 | 0.1 |
| 0.4Pr-CoRu/AOmM | Pr | 0.4 | 1.9 | 0.4 |
| 0.7Pr-CoRu/AOmM | Pr | 0.7 | 3.1 | 0.7 |
| 1.0Pr-CoRu/AOmM | Pr | 1.0 | 5.1 | 1.1 |
| 2.0Pr-CoRu/AOmM | Pr | 2.0 | 8.6 | 2.0 |
| 3.0Pr-CoRu/AOmM | Pr | 3.0 | 15.1 | 3.2 |
| 1.0Sm-CoRu/AOmM | Sm | 1.0 | 5.6 | 1.1 |
| 1.0Pr-CoRu/AOm(7) | Pr | 1.0 | 4.5 | 1.0 |
| 1.0Pr-CoRu/AOm(11) | Pr | 1.0 | 2.9 | 1.0 |
| 1.0Pr-CoRu/AOm(24) | Pr | 1.0 | 2.3 | 1.1 |

[a] Nominal surface-specific promoter loading. [b] Experimental promoter weight loading. For catalysts supported on the meso-macroporous γ-$Al_2O_3$ (AOmM), potassium was detected at impurity levels (≤0.4 wt%), likely as a result of an adventitious potassium impurity in the surfactant used as porogen agent in the soft-templating route applied to synthesize this hierarchically porous material. [c] Experimental surface-specific promoter content.

**Table S3:** XPS binding energies (BEs) for metallic and Pr species in selected catalysts after reductive activation and following exposure to FT synthesis conditions.

| Catalyst | State | BE $Co2p_{3/2}$ ($Co^0$) (eV) | BE $Ru3p_{5/2}$ ($Ru^0$) (eV) | BE $Pr3d_{5/2}$ ($Pr^{3+}/Pr^{4+}$) (eV) |
|---|---|---|---|---|
| CoRu/AOmM | Reduced[a] | 777.2 | 279.5 | n.a. |
| 1.0Pr-CoRu/AOmM | Reduced | 777.5 | 279.6 | 929.5 / 933.7 |
| 1.0Pr-CoRu/AOmM | After *in situ* FT[b] | 777.4 | 280.2 | 929.6 / 933.7 |
| 3.0Pr-CoRu/AOmM | Reduced | 777.5 | 280.2 | 929.6 / 933.7 |
| Ru/AOmM | Reduced | n.a. | 280.1 | n.a. |

[a] Following *ex situ* reduction in a packed bed under hydrogen flow at 673 K, passivation at RT under flow of 1%$O_2$/He, and further reduction at 673 K under flow of $H_2$ once conformed as a pellet, in a reaction chamber connected to the XP spectrometer. [b] Following exposure of the *in situ* reduced catalyst to Fischer-Tropsch reaction conditions for 2 h in the high-pressure reactor unit coupled to our XP spectrometer, evacuation and transfer under UHV conditions to the XPS chamber. Reaction conditions: T=473 K, P=10 bar, $H_2$/CO=2.0.

Core-electron binding energies (BEs), as determined by XPS, have been used to assess potential electronic modifications of Co and Ru metallic phases upon promotion with $PrO_x$, as well as following exposure to Fischer-Tropsch reaction conditions *in situ*, in a high-pressure reactor unit coupled to the XP spectrometer. As shown in Table S3, the $Co2p_{3/2}$ BE for $Co^0$ increased slightly from 777.2 eV to 777.4-777.5 eV upon incorporation of praseodymium as promoter at surface contents of 1.0-3.0 $Pr_{at}$ $nm^{-2}$. This result hints towards a slight decrease in the electron density on near-surface $Co^0$ atoms, in line with the overall electron withdrawing character predicted by our DFT calculations for $PrO_x$ species on the metal surface. With regard to Ru, a significant shift in the $Ru3p_{5/2}$ BE was observed, from 279.5-6 to 280.2 eV, upon exposure of 1.0Pr-CoRu/AOmM to Fischer-Tropsch synthesis conditions. The latter BE coincides with that observed for a Co-free $Ru/Al_2O_3$ reference catalyst after reductive activation (280.1 eV), and it is thus characteristic of Ru clusters. These observations suggest that, while the $Ru^0$ reduction promoter appears to be highly intermixed with $Co^0$ after reduction of the bimetallic FT catalysts, thus likely withdrawing electronic density from the lower electronegativity cobalt, certain Ru segregation takes place during exposure to syngas conversion conditions, leading to Ru-enriched agglomerates on the catalyst surface. Regarding Pr, no relevant changes, neither in the $Pr3d_{5/2}$ BE, nor in the near-surface $Pr^{4+}/Pr^{3+}$ ratio (2.9±0.2) were observed following exposure of 1.0Pr-CoRu/AOmM to *in situ* syngas conversion conditions, suggesting a negligible further reduction or redistribution under catalysis conditions, as expected considering that the majority of the $PrO_x$ species are not interacting directly with the active metal but rather strongly bonded to the $\gamma$-$Al_2O_3$ support.

**Table S4:** Cobalt-Time-Yield (CTY) and product selectivities for CoRu-based FT catalysts supported on unimodally mesoporous or multimodally meso-macroporous γ-$Al_2O_3$, alternatively modified with various alkali and lanthanide oxide promoters at different promoter surface-specific contents. Reaction conditions: T=473 K, P=20 bar, $H_2$/CO=2, WHSV=5.1-11.0 $h^{-1}$, CO conversion=20±3%.

| Catalyst | CTY[a] [mmolCO $g_{Co}^{-1}h^{-1}$] | *S*($CO_2$) [C%] | *S*($CH_4$) [C%] | *S*($C_{5+}$) [C%] | *S*($C_{5-10}$ Olef.) [C%] | α[b] [-] | $C_{2-4}$ Olef.[c] [%] | $C_{5-10}$ Olef.[d] [%] |
|---|---|---|---|---|---|---|---|---|
| CoRu/AOm(7) | 145.9 | 0.2 | 10.6 | 74.3 | 9.9 | 0.79 | 47.9 | 33.4 |
| 1.0Pr-CoRu/AOm (7) | 74.3 | 0.3 | 10.9 | 69.3 | 17.9 | 0.76 | 57.3 | 53.2 |
| CoRu/AOm(11) | 173.9 | 0.2 | 11.6 | 76.6 | 11.2 | 0.82 | 50.4 | 36.6 |
| 1.0Pr-CoRu/AOm (11) | 89.9 | 0.3 | 10.4 | 70.4 | 16.3 | 0.76 | 56.0 | 49.0 |
| CoRu/AOm(24) | 241.5 | 0.2 | 9.3 | 80.8 | 9.8 | 0.83 | 55.2 | 43.7 |
| 1.0Pr-CoRu/AOm (24) | 117.8 | 0.3 | 8.6 | 74.9 | 16.3 | 0.77 | 59.8 | 54.1 |
| CoRu/AOmM | 88.9 | 0.6 | 7.9 | 79.2 | 13.3 | 0.80 | 56.5 | 54.7 |
| 0.4Na-CoRu/AOmM | 59.0 | 1.3 | 7.4 | 79.3 | 14.6 | 0.80 | 59.1 | 58.0 |
| 1.0Na-CoRu/AOmM | 32.9 | 1.0 | 8.0 | 76.0 | 14.4 | 0.77 | 54.3 | 56.2 |
| 3.0Na-CoRu/AOmM | 8.5 | 8.1 | 10.8 | 53.2 | 10.1 | 0.66 | 36.1 | 53.1 |
| 1.0K-CoRu/AOmM | 27.5 | 1.3 | 8.6 | 74.4 | 13.9 | 0.78 | 54.1 | 57.2 |
| 1.0Cs-CoRu/AOmM | 10.9 | 2.9 | 11.4 | 65.0 | 12.1 | 0.71 | 56.7 | 54.6 |
| 1.0La-CoRu/AOmM | 43.1 | 0.8 | 8.2 | 73.2 | 16.2 | 0.75 | 54.7 | 58.1 |
| 0.1Pr-CoRu/AOmM | 78.5 | 0.6 | 8.2 | 77.1 | 14.6 | 0.78 | 57.9 | 57.2 |
| 0.4Pr-CoRu/AOmM | 59.7 | 0.7 | 8.0 | 76.6 | 15.4 | 0.78 | 58.1 | 57.2 |
| 0.7Pr-CoRu/AOmM | 52.4 | 0.7 | 8.1 | 74.2 | 16.0 | 0.76 | 56.6 | 58.0 |
| 1.0Pr-CoRu/AOmM | 36.7 | 0.9 | 8.4 | 70.2 | 19.9 | 0.75 | 57.5 | 61.5 |
| 2.0Pr-CoRu/AOmM | 33.6 | 0.9 | 8.4 | 66.7 | 17.8 | 0.72 | 55.7 | 62.2 |
| 3.0Pr-CoRu/AOmM | 12.4 | 1.3 | 12.4 | 54.6 | 19.5 | 0.66 | 53.0 | 61.7 |
| 1.0Sm-CoRu/AOmM | 47.3 | 1.1 | 9.2 | 73.6 | 14.6 | 0.76 | 54.8 | 53.2 |

[a] Cobalt-time-yield. [b] Chain-growth probability. [c] Molar olefin abundance within the $C_{2-4}$ hydrocarbon product fraction. [d] Molar olefin abundance within the $C_{5-10}$ hydrocarbon product fraction.

**Table S5:** Optimized adsorption sites and energies for $H^*$ and $CO^*$ on $PrO_2$- and $Na_2O$-covered Co(111) surfaces for an adsorbate/promoter coverage of 0.11 ML as derived from DFT calculations. NA indicates that the specific adsorbate/site configuration is not energetically favorable and converges to another site.

| Adsorption site | $PrO_2$-Co(111) | | $Na_2O$-Co(111) | |
|---|---|---|---|---|
| | $E_{ads}[H^*]$ (kJ $mol^{-1}$) | $E_{ads}[CO^*]$ (kJ $mol^{-1}$) | $E_{ads}[H^*]$ (kJ $mol^{-1}$) | $E_{ads}[CO^*]$ (kJ $mol^{-1}$) |
| hcp1 | 262 | 240 | 265 | 234 |
| hcp2 | 271 | NA | 269 | NA |
| fcc1 | 276 | NA | 261 | NA |
| fcc2 | 273 | 226 | 265 | 219 |

**Table S6:** Optimized adsorption sites and energies for $H^*$ and $CO^*$ on $PrO_2$- and $Na_2O$- covered Co(211) surfaces for an adsorbate/promoter coverage of 0.11 ML as derived from DFT calculations. NA indicates that the specific adsorbate/site configuration is not energetically favorable and converges to another site.

| Adsorption site | $PrO_2$-Co(211) | | $Na_2O$-Co(211) | |
|---|---|---|---|---|
| | $E_{ads}[H^*]$ (kJ $mol^{-1}$) | $E_{ads}[CO^*]$ (kJ $mol^{-1}$) | $E_{ads}[H^*]$ (kJ $mol^{-1}$) | $E_{ads}[CO^*]$ (kJ $mol^{-1}$) |
| hcp1 | 275 | 232 | NA | NA |
| hcp2 | 270 | NA | 277 | 222 |
| fcc1 | NA | NA | 262 | NA |
| fcc2 | 270 | NA | 270 | NA |

**Table S7:** Adsorption energies for $H^*$/$CO^*$ for their most stable configurations on $PrO_2$- and $Na_2O$-covered Co(111) and Co(211) surfaces and the corresponding Bader charges on metal atoms/adsorbates, for a promoter/adsorbate coverage of 0.11 ML. For the cobalt surfaces, Bader charges are reported as the sum of charges on the uppermost 2 layers (18 atoms) for the Co(111) surface and the uppermost 3 layers (18 atoms) for the Co(211) surface. For the oxide promoter units, Bader charges are reported as the sum of charges on the $O^{2-}$ anions and cations of the $PrO_2$/ $Na_2O$ units.

| Surface | $H^*$ | | $CO^*$ | | | |
|---|---|---|---|---|---|---|
| | $E_{ads}$ (kJ $mol^{-1}$) | Bader charge on $H^*$ (e) | $E_{ads}$ (kJ $mol^{-1}$) | Bader charges (e) | | |
| | | | | $CO^*$ | Co | Promoter oxide |
| Bare-Co(111) | 280 | -0.38 | 170 | -0.62 | 0.62 | 0 |
| $PrO_2$-Co(111) | 276 | -0.44 | 240 | -1.06 | 1.65 | -0.61 |
| $Na_2O$-Co(111) | 269 | -0.43 | 234 | -1.17 | 0.73 | 0.42 |
| Bare-Co(211) | 284 | -0.40 | 177 | -0.63 | 0.63 | 0 |
| $PrO_2$-Co(211) | 275 | -0.43 | 232 | -0.98 | 1.50 | -0.51 |
| $Na_2O$-Co(211) | 277 | -0.45 | 222 | -0.97 | 0.50 | 0.48 |

## 5. References


1. Duyckaerts, N.; Bartsch, M.; Trotuş, I.-T.; Pfänder, N.; Lorke, A.; Schüth, F.; Prieto, G. Intermediate product regulation in tandem solid catalysts with multimodal porosity for high-yield synthetic fuel production. *Angew. Chem. Int. Ed.* **2017,** 56 (38), 11480–11484.
2. Reuel, R. C.; Bartholomew, C. H. Effects of support and dispersion on the CO hydrogenation activity/selectivity properties of cobalt. *J. Catal.* **1984,** 85 (1), 78-88.
3. Meyer, F.; Beucher, S. Morphological segmentation. *J. Vis. Commun. Image Represent.* **1990,** 1 (1), 21-46.
4. Borgefors, G. On digital distance transforms in three dimensions. *Comput. Vis. Image Underst.* **1996,** 64 (3), 368-376.
5. Raeini, A. Q.; Bijeljic, B.; Blunt, M. J. Generalized network modeling: Network extraction as a coarse-scale discretization of the void space of porous media. *Phys. Rev. E* **2017,** 96 (1), 013312.
6. Kresse, G.; Hafner, J. Ab initio molecular dynamics for liquid metals. *Phys. Rev. B* **1993,** 47 (1), 558-561.
7. Kresse, G.; Furthmüller, J. Efficient iterative schemes for ab initio total-energy calculations using a plane-wave basis set. *Phys. Rev. B* **1996,** 54 (16), 11169-11186.
8. Perdew, J. P.; Burke, K.; Ernzerhof, M. Generalized gradient approximation made simple. *Phys. Rev. Lett.* **1996,** 77 (18), 3865-3868.
9. Blöchl, P. E. Projector augmented-wave method. *Phys. Rev. B* **1994,** 50 (24), 17953-17979.
10. van Helden, P.; Ciobîcă, I. M.; Coetzer, R. L. J. The size-dependent site composition of FCC cobalt nanocrystals. *Catal. Today* **2016,** 261, 48-59.
11. Kizilkaya, A. C.; Niemantsverdriet, J. W.; Weststrate, C. J. Effect of ammonia on cobalt Fischer–Tropsch synthesis catalysts: a surface science approach. *Catal. Sci. Technol.* **2019,** 9 (3), 702-710.
12. Monkhorst, H. J.; Pack, J. D. Special points for Brillouin-zone integrations. *Phys. Rev. B* **1976,** 13 (12), 5188-5192.
13. Henkelman, G.; Arnaldsson, A.; Jónsson, H. A fast and robust algorithm for Bader decomposition of charge density. *Comput. Mater. Sci.* **2006,** 36 (3), 354-360.
14. Sanville, E.; Kenny, S. D.; Smith, R.; Henkelman, G. Improved grid-based algorithm for Bader charge allocation. *J. Comput. Chem.* **2007,** 28 (5), 899-908.
15. Tang, W.; Sanville, E.; Henkelman, G. A grid-based Bader analysis algorithm without lattice bias. *J. Phys.: Condens. Matter* **2009,** 21 (8), 084204.
16. Toomes, R. L.; King, D. A. The adsorption of CO on Co{1010}. *Surf. Sci.* **1996,** 349 (1), 1-18.
17. Manoilova, O. V.; Podkolzin, S. G.; Tope, B.; Lercher, J.; Stangland, E. E.; Goupil, J.-M.; Weckhuysen, B. M. Surface Acidity and Basicity of $La_2O_3$, LaOCl, and $LaCl_3$ Characterized by IR Spectroscopy, TPD, and DFT Calculations. *J. Phys. Chem. B* **2004,** 108 (40), 15770-15781.
