# Supplementary material for: Design of Cobalt Fischer–Tropsch Catalysts for the Combined Production of Liquid Fuels and Olefin Chemicals from Hydrogen-Rich Syngas
Source: ACS Catal. 2021 Apr 5;11(8):4784–98. doi: 10.1021/acscatal.0c05027 (PMC8056389; doi:10.1021/acscatal.0c05027)
Supplement: Supplementary file 2 — cs0c05027_si_002.pdf [file cs0c05027_si_002.pdf]

Supporting Information (cont)

Design of cobalt Fischer-Tropsch catalysts for the combined production of liquid fuels and olefin chemicals from hydrogen-rich syngas

Kai Jeske[1], Ali Can Kizilkaya[2], Iván López-Luque[3], Norbert Pfänder[4], Matthias Bartsch,[5] Patricia Concepción[3], Gonzalo Prieto[1,3,*]

[1] Max-Planck-Institut für Kohlenforschung, Kaiser-Wilhelm-Platz 1, 45470 Mülheim an der Ruhr, Germany.

[2] Department of Chemical Engineering, Izmir Institute of Technology, Gülbahçe Kampüsü, 35430 Izmir, Turkey.

[3] ITQ Instituto de Tecnología Química, Universitat Politècnica de València-Consejo Superior de Investigaciones Científicas (UPV-CSIC), Avenida de los Naranjos s/n, 46022 Valencia, Spain.

[4] Max-Planck-Institut für chemische Energiekonversion, Stiftstraße, 45470 Mülheim an der Ruhr, Germany.

[5] Faculty of Physics and CENIDE, Universität Duisburg-Essen, 47048 Duisburg, Germany.

[*]Email: prieto@mpi-muelheim.mpg.de; prieto@itq.upv.es

6. Structure files (DFT calculations)

i) Absolute energies of the structures reported in the manuscript (eV)

1) PrO_2-Co(111)-2x2	-128.89302516
2) PrO_2-Co(211)-1x2	-238.57085732
3) Na_2O-Co(111)-2x2	-117.56242049
4) Na_2O-Co(211)-1x2	-227.52687570
5) PrO_2-Co(111)-3x3	-262.49976992
6) PrO_2-Co(211)-1x3	-346.22925763
7) Na_2O-Co(111)-3x3	-252.23074347
8) Na_2O-Co(211)-1x3	-335.67823594
9) H-Co(111)-3x3	-244.23380844
10) H-PrO_2-Co(111)-3x3	-266.33876573
11) H-Na_2O-Co(111)-3x3	-255.99209598

12) CO-Co(111)-3x3	-256.82825463
13) CO-PrO_2-Co(111)-3x3	-279.67890552
14) CO-Na_2O-Co(111)-3x3	-269.35040455
15) H-Co(211)-1x3	-326.93677101
16) H-PrO_2-Co(211)-1x3	-350.05488436
17) H-Na_2O-Co(211)-1x3	-339.52004017
18) CO-Co(211)-1x3	-339.56825882
19) CO-PrO_2-Co(211)-1x3	-363.32903626
20) CO-Na_2O-Co(211)-1x3	-352.67612255
21) C_2H_4-Co(111)-3x3	-272.86550325
22) C_2H_4-PrO_2-Co(111)-3x3	-294.62661654
23) C_2H_4-Co(211)-1x3	-356.23248497
24) C_2H_4-PrO_2-Co(211)-1x3	-378.9933074
25) C-Co(111)-3x3	-248.51457208
26) C-PrO_2 -Co(111)-3x3	-270.88401842
27) C-Na_2O-Co(111)-3x3	-260.63216452
28) C-Co(211)-1x3	-331.33418986
29) C-PrO_2 -Co(211)-1x3	-354.63076785
30) C-Na_2O-Co(211)-1x3	-344.1383606

ii) VASP POSCAR files

1) PrO_2-Co(111)-2x2

```
Co Pr O
   1.00000000000000
     4.9752000000000001    0.0000000000000000    0.0000000000000000
    -2.4876000000000000    4.3086495888999998    0.0000000000000000
     0.0000000000000000    0.0000000000000000   21.0933999999999990
   Co   Pr   O
    16     1     2
Selective dynamics
Direct
  0.4445037044393629  0.4722398417457470  0.0024679757649295   F   F   F
  0.9445037044393629  0.4722398417457470  0.0024679757649295   F   F   F
  0.4445037282925455  0.9722398894521049  0.0024679757649295   F   F   F
  0.9445037282925455  0.9722398894521049  0.0024679757649295   F   F   F
  0.1114513153999539  0.3057535714656083  0.0948550731508462   F   F   F
  0.6114513153999539  0.3057535714656083  0.0948550731508462   F   F   F
  0.1114513392531364  0.8057536191719734  0.0948550731508462   F   F   F
  0.6114513392531364  0.8057536191719734  0.0948550731508462   F   F   F
```

```
0.2731032588298067  0.1388428258455599  0.1928027940928242   T   T   T
0.7775714905794112  0.1383873554515214  0.1934177543917787   T   T   T
0.2746379882948871  0.6356880804870526  0.1937469262252913   T   T   T
0.7744995809387224  0.6377803447482050  0.1948657948326394   T   T   T
0.4455856674148032  0.4781918645404295  0.2898307587559638   T   T   T
0.9412495153114611  0.4687342373096127  0.2865710806432946   T   T   T
0.4452219384921141  0.9698445726518042  0.2839912253299769   T   T   T
0.9317520968679313  0.9641852533621050  0.2899777763113320   T   T   T
0.4084913512254580  0.8451342412281843  0.4189876214004703   T   T   T
0.2807349002319417  0.1328592794712665  0.3490955823335268   T   T   T
0.9516422081300646  0.4949668301481068  0.4390906674975716   T   T   T
```

2) PrO_2-Co(211)-1x2

```
Co Pr O
  1.00000000000000
    6.0933999999999999    0.0000000000000000    0.0000000000000000
    0.0000000000000000    4.9752000000000001    0.0000000000000000
    0.0000000000000000    0.0000000000000000   25.7715999999999994
  Co   Pr   O
   32    1    2
Selective dynamics
Direct
0.3012132799999989  0.0000000000000000  0.0046948579056050   F   F   F
0.3012132799999989  0.5000000000000000  0.0046948579056050   F   F   F
0.6286634719999995  0.2500000000000000  0.0308760418445075   F   F   F
0.6286634719999995  0.7500000000000000  0.0308760418445075   F   F   F
0.9633304890000005  0.0000000000000000  0.0539849291468144   F   F   F
0.9633304890000005  0.5000000000000000  0.0539849291468144   F   F   F
0.2960606560000016  0.2500000000000000  0.0837748529388946   F   F   F
0.2960606560000016  0.7500000000000000  0.0837748529388946   F   F   F
0.6289869370000005  0.0000000000000000  0.1118942944947179   F   F   F
0.6289869370000005  0.5000000000000000  0.1118942944947179   F   F   F
0.9626366230000016  0.2500000000000000  0.1384130593366351   F   F   F
0.9626366230000016  0.7500000000000000  0.1384130593366351   F   F   F
0.2974300059999990  0.0000000000000000  0.1677073600397350   F   F   F
0.2974300059999990  0.5000000000000000  0.1677073600397350   F   F   F
0.6301967699999977  0.2500000000000000  0.1957913361995409   F   F   F
0.6301967699999977  0.7500000000000000  0.1957913361995409   F   F   F
0.9612393345043201  0.0001654511557178  0.2217530779742082   T   T   T
0.9619602252223010  0.5007080034786174  0.2223508555645184   T   T   T
0.2932882650735641  0.2509469490134886  0.2503303216932884   T   T   T
0.2952132657754088  0.7518241800958301  0.2508991737638424   T   T   T
0.6274213143218794  0.0033536120609225  0.2791209538839414   T   T   T
0.6295366930833761  0.4967964024061680  0.2797256661253752   T   T   T
0.9619202854679206  0.2459953049042675  0.3048451383238431   T   T   T
0.9626914900602375  0.7534252876280481  0.3061211429073926   T   T   T
0.2949105437012648  0.0047520241691534  0.3350009482826016   T   T   T
0.2976579892100570  0.4967995017534598  0.3346541804789939   T   T   T
0.6192171601653956  0.2452566944705538  0.3676970544897814   T   T   T
0.6224272945515560  0.7539016396365075  0.3595286477993909   T   T   T
0.9653321004684635  0.0031621961181977  0.3895328192885472   T   T   T
0.9644658444022296  0.4975548052334668  0.3838548951817233   T   T   T
0.2915578224101827  0.2605495254074769  0.4194275015122335   T   T   T
```

0.2763778939310634 0.7568547364505791 0.4150143802033285   T   T   T
0.7026291092202090 0.6726542584438787 0.4674838650212905   T   T   T
0.8059941344833528 0.2513628050176295 0.4376669279132808   T   T   T
0.3552186555693522 0.5245467981836430 0.4712444666277791   T   T   T

3) Na_2O-Co(111)-2x2

Co O Na
1.00000000000000
4.9752000000000001   0.0000000000000000   0.0000000000000000
-2.4876000000000000   4.3086495888999998   0.0000000000000000
0.0000000000000000   0.0000000000000000   21.0933999999999990
Co   O   Na
16   1   2
Selective dynamics
Direct
0.4445036999999985 0.4722397999999970 0.0024680000000004   F   F   F
0.9445036999999985 0.4722397999999970 0.0024680000000004   F   F   F
0.4445036999999985 0.9722398999999982 0.0024680000000004   F   F   F
0.9445036999999985 0.9722398999999982 0.0024680000000004   F   F   F
0.1114512999999988 0.3057536000000027 0.0948550999999966   F   F   F
0.6114512999999988 0.3057536000000027 0.0948550999999966   F   F   F
0.1114512999999988 0.8057536000000027 0.0948550999999966   F   F   F
0.6114512999999988 0.8057536000000027 0.0948550999999966   F   F   F
0.2776521855817350 0.1381852048054523 0.1930355853846610   T   T   T
0.7784782771831753 0.1359161925104969 0.1935274566575672   T   T   T
0.2781755830846162 0.6413971381731766 0.1922670644672179   T   T   T
0.7784940572443121 0.6414059959255419 0.1930918701225801   T   T   T
0.4376959212810057 0.4736629485519732 0.2852953436066404   T   T   T
0.9414012107139454 0.4704683161905035 0.2824699997488989   T   T   T
0.4454287581270113 0.9716778432436751 0.2883652703952925   T   T   T
0.9502930257784340 0.9725144480852879 0.2931521944187467   T   T   T
0.6039263627627998 0.7947550804827977 0.3527009919514289   T   T   T
0.2071085627995353 0.6783597355252474 0.4178522269062981   T   T   T
0.5498419048112312 0.3488324443604114 0.4050519521764563   T   T   T

4) Na_2O-Co(211)-1x2

Co O Na
1.00000000000000
6.0933999999999999   0.0000000000000000   0.0000000000000000
0.0000000000000000   4.9752000000000001   0.0000000000000000
0.0000000000000000   0.0000000000000000   25.7715999999999994
Co   O   Na
32   1   2
Selective dynamics
Direct
0.3012132799999989 0.0000000000000000 0.0046948579056121   F   F   F
0.3012132799999989 0.5000000000000000 0.0046948579056121   F   F   F
0.6286634719999995 0.2500000000000000 0.0308760418445075   F   F   F
0.6286634719999995 0.7500000000000000 0.0308760418445075   F   F   F
0.9633304890000005 0.0000000000000000 0.0539849291468073   F   F   F
0.9633304890000005 0.5000000000000000 0.0539849291468073   F   F   F

```
0.2960606560000016  0.2500000000000000  0.0837748529388875   F   F   F
0.2960606560000016  0.7500000000000000  0.0837748529388875   F   F   F
0.6289869370000005  0.0000000000000000  0.1118942944947179   F   F   F
0.6289869370000005  0.5000000000000000  0.1118942944947179   F   F   F
0.9626366230000016  0.2500000000000000  0.1384130593366422   F   F   F
0.9626366230000016  0.7500000000000000  0.1384130593366422   F   F   F
0.2974300059999990  0.0000000000000000  0.1677073600397421   F   F   F
0.2974300059999990  0.5000000000000000  0.1677073600397421   F   F   F
0.6301967699999977  0.2500000000000000  0.1957913361995409   F   F   F
0.6301967699999977  0.7500000000000000  0.1957913361995409   F   F   F
0.9619978788376849 -0.0003490539629597  0.2221417784026110   T   T   T
0.9606365063938908  0.4996067533403309  0.2216319089297358   T   T   T
0.2934335824692851  0.2502414615288501  0.2505753684110027   T   T   T
0.2939168337344539  0.7486304575455618  0.2507821647662609   T   T   T
0.6289541009653027 -0.0005456994246533  0.2797308738848672   T   T   T
0.6255320386667752  0.4990560074259988  0.2795970363039872   T   T   T
0.9613166009727856  0.2533160153964640  0.3055465320799379   T   T   T
0.9626608326994592  0.7459595917245979  0.3052787713899660   T   T   T
0.2948539972108672  0.0019008051795465  0.3340351808133730   T   T   T
0.2929097363045683  0.4975662258682926  0.3347014665044555   T   T   T
0.6230062937322909  0.2536507104197284  0.3629776697897348   T   T   T
0.6253553230172375  0.7463329594566810  0.3626999500570093   T   T   T
0.9576911850290702 -0.0022316683258602  0.3835783501964002   T   T   T
0.9607062413742035  0.4996541716991075  0.3888888756724684   T   T   T
0.2751859196504718  0.2415330201427859  0.4183535254682993   T   T   T
0.2919632663196226  0.7541449270527810  0.4149315122915813   T   T   T
0.3130056802450303  0.9883250648918248  0.4724912681101834   T   T   T
0.6831934584206851  0.0538965219853949  0.4720938927767471   T   T   T
0.1613909137757520  0.5974497287993746  0.5095076993344319   T   T   T
```

5) PrO_2-Co(111)-3x3

```
Co111_3X3
  1.00000000000000
    7.4587998390197754    0.0000000000000000    0.0000000000000000
   -3.7294002959821051    6.4595099249780574    0.0000000000000000
   -0.0000010106117090   -0.0000017504309440   23.1201000213622159
  Co   Pr   O
  36    1    2
Selective dynamics
Direct
  0.0000352770987675  0.9999648825964869  0.0907110695049838   F   F   F
  0.3333557427970177  0.0000001793936022  0.0907175141137841   F   F   F
  0.6666918534736581  0.9999938321653516  0.0907267268766958   F   F   F
  0.0000062578975388  0.3333084372988537  0.0907267268766958   F   F   F
  0.3333357007148621  0.3333426504206471  0.0907265971194704   F   F   F
  0.6666945969032270  0.3333051862794676  0.0907313116319486   F   F   F
  0.9999998575876035  0.6666444062796870  0.0907175141137841   F   F   F
  0.3333385397113773  0.6666614354387903  0.0907302303217463   F   F   F
  0.6666573639132167  0.6666642220269168  0.0907265971194704   F   F   F
  0.1111175321760314  0.2222028177229376  0.1750651163386081   F   F   F
  0.4444257597051688  0.2222195372577005  0.1750655488626904   F   F   F
  0.7777781372906531  0.2222218594174734  0.1750735938105805   F   F   F
  0.1111123692640206  0.5555522552209098  0.1750674087162309   F   F   F
```

```
 0.4444440697289238  0.5555561254847916  0.1750719502190776   F   F   F
 0.7777804782179913  0.5555742383122606  0.1750655488626904   F   F   F
 0.1111199658994551  0.8888800192425492  0.1750494157144900   F   F   F
 0.4444476042812084  0.8888872953412061  0.1750674087162309   F   F   F
 0.7777970141657633  0.8888823414043898  0.1750651163386081   F   F   F
 0.2205507349655056  0.1094534330080543  0.2654766529655608   T   T   T
 0.5554005973357555  0.1113674967882625  0.2649708104635769   T   T   T
 0.8896503450394350  0.1122865792695520  0.2658869950440838   T   T   T
 0.2179021402998755  0.4457232492530531  0.2640837498937665   T   T   T
 0.5584798439606136  0.4445939023372237  0.2633480531223145   T   T   T
 0.8901559781317090  0.4448783373487781  0.2669891098999089   T   T   T
 0.2196254967451199  0.7749854356544967  0.2666214237726218   T   T   T
 0.5537604336581013  0.7775207297538278  0.2652377348272291   T   T   T
 0.8897381939651556  0.7773281259294238  0.2657989644324691   T   T   T
 0.9925022627991740 -0.0063480794855958  0.3517869473176718   T   T   T
 0.3373528351197560 -0.0029213908661792  0.3509113587001192   T   T   T
 0.6649668686147857 -0.0006575821916713  0.3492862213176870   T   T   T
-0.0068948876795418  0.3296442740893500  0.3543386472388838   T   T   T
 0.3393333738924498  0.3408694234210600  0.3456303495064795   T   T   T
 0.6638482412461191  0.3313166729382071  0.3503934128369377   T   T   T
-0.0062826353240214  0.6677193866483342  0.3545378466138289   T   T   T
 0.3378884539168539  0.6684976163161643  0.3531670235977714   T   T   T
 0.6654273185013881  0.6649922329133168  0.3483132922319320   T   T   T
 0.2925201052479974  0.2097423465642124  0.4515667883134011   T   T   T
 0.2236252164455444  0.4423866230300575  0.4102540780261008   T   T   T
 0.1141684589323647 -0.1041958024132253  0.4127388392188282   T   T   T
```

6) PrO_2-Co(211)-1x3

```
Co(211)-3x3
   1.00000000000000
     6.0968000000000000    0.0000000000000000    0.0000000000000000
     0.0000000000000000    7.4669999999999996    0.0000000000000000
     0.0000000000000000    0.0000000000000000   23.6221999999999994
   Co   Pr   O
   48    1    2
Selective dynamics
Direct
 0.0039768403096687  0.1666663988214836  0.9745822997011260   F   F   F
 0.0039765122687285  0.5000000000000000  0.9745820457027676   F   F   F
 0.0039768403096687  0.8333336011785164  0.9745822997011260   F   F   F
 0.3315888662905095  0.0000000000000000  0.0034278771663949   F   F   F
 0.3315855858811148  0.3333350743270387  0.0034291471581795   F   F   F
 0.3315855858811148  0.6666647917503710  0.0034291471581795   F   F   F
 0.6664853693741009  0.1666655952859273  0.0285661792720404   F   F   F
 0.6664884857630256  0.5000000000000000  0.0285654596100287   F   F   F
 0.6664853693741009  0.8333344047140727  0.0285661792720404   F   F   F
 0.9983825941477491  0.0000000000000000  0.0609119811025209   F   F   F
 0.9983791497178842  0.3333345386366702  0.0609079171288016   F   F   F
 0.9983791497178842  0.6666654613633298  0.0609079171288016   F   F   F
 0.3314264860254568  0.1666650595955517  0.0918800111759310   F   F   F
 0.3314281262301506  0.5000000000000000  0.0918805615057039   F   F   F
 0.3314264860254568  0.8333349404044483  0.0918800111759310   F   F   F
 0.6654751673008761  0.0000000000000000  0.1205095207050988   F   F   F
```

0.6654631938065876 0.3333330654881479 0.1205040597404121 F F F
0.6654631938065876 0.6666669345118521 0.1205040597404121 F F F
0.0009121178323070 0.1666669345118521 0.1526111031148645 F F F
0.0009114617504267 0.5000000000000000 0.1526112301140472 F F F
0.0009121178323070 0.8333330654881479 0.1526111031148645 F F F
0.3340214538774404 0.0000000000000000 0.1833969740329024 F F F
0.3340229300616713 0.3333327976429601 0.1833968470337197 F F F
0.3340229300616713 0.6666672023570399 0.1833968470337197 F F F
0.6647434005567515 0.1665774013671639 0.2117360831755252 T T T
0.6661519577181387 0.4996734608718044 0.2119139648101470 T T T
0.6659141096602573 0.8342002262019684 0.2118690743323975 T T T
0.9964393114311343 0.0009227820541037 0.2431542600300359 T T T
-0.0011432956328016 0.3333778118860782 0.2429869856777780 T T T
-0.0002089003064927 0.6661987203426186 0.2427287716086656 T T T
0.3314599722444967 0.1671835516208988 0.2757971695707473 T T T
0.3345632533814381 0.4985795541405580 0.2755451716167761 T T T
0.3318229527220464 0.8350489727305822 0.2736918115721638 T T T
0.6649016366256801 0.0018221812058383 0.3035486815530158 T T T
0.6682134036001685 0.3294688816297060 0.3021783541257580 T T T
0.6659778889987896 0.6676940121105575 0.3019670862987719 T T T
0.9982792811797545 0.1684834865515520 0.3353824638116968 T T T
0.0029077364228150 0.4989424816448920 0.3347768770371820 T T T
0.9973720717684887 0.8325619969512291 0.3343715689230554 T T T
0.3283272693411702 0.9965530945411605 0.3663266051835712 T T T
0.3256942897623311 0.3313773457506297 0.3697735229751465 T T T
0.3274083206636151 0.6745745372857700 0.3642271721797583 T T T
0.6711602876081163 0.1608480675955824 0.3966851276477509 T T T
0.6699000001952580 0.5008209129673641 0.3872377107990902 T T T
0.6639892542033518 0.8347984798637287 0.3912170579799690 T T T
0.9950575859126305 0.9952136632865903 0.4202637815395033 T T T
0.9970782071186626 0.3386946583382129 0.4265180209270397 T T T
0.9756689453573376 0.6708183985952250 0.4239015393492523 T T T
0.4024443334021542 0.5932255357087505 0.4823653893281696 T T T
0.5172612014174336 0.3406377852776554 0.4413523363336903 T T T
0.0514696918509387 0.5117638694791101 0.4847024061000213 T T T

7) Na_2O-Co(111)-3x3

Co O Na
1.00000000000000
7.4587998390197754 0.0000000000000000 0.0000000000000000
-3.7294002959821051 6.4595099249780574 0.0000000000000000
-0.0000010106117090 -0.0000017504309440 23.1201000213622159
Co O Na
36 1 2
Selective dynamics
Direct
0.0000353225999987 0.9999648941999979 0.0907110695000028 F F F
0.3333557428000020 0.0000001793999971 0.0907175140999996 F F F
0.6666917649000013 0.9999938437999987 0.0907267269000016 F F F
0.0000062578999973 0.3333084372999977 0.0907267269000016 F F F
0.3333357006999975 0.3333426503999988 0.0907265971000015 F F F
0.6666945969000011 0.3333051863000023 0.0907313116000026 F F F
0.9999998791999971 0.6666444063000014 0.0907175140999996 F F F

```
 0.3333385397000015  0.6666614354000018  0.0907302302999966   F   F   F
 0.6666573639000006  0.6666642220000014  0.0907265971000015   F   F   F
 0.1111175321999980  0.2222028177000013  0.1750651162999972   F   F   F
 0.4444257597000032  0.2222195372999991  0.1750655489000010   F   F   F
 0.7777781372999968  0.2222218594000012  0.1750735938000005   F   F   F
 0.1111123693000025  0.5555522551999985  0.1750674087000021   F   F   F
 0.4444440696999976  0.5555561254999972  0.1750719501999995   F   F   F
 0.7777804782000004  0.5555742382999966  0.1750655489000010   F   F   F
 0.1111199659000022  0.8888800192000019  0.1750494157000020   F   F   F
 0.4444476043000023  0.8888872953000018  0.1750674087000021   F   F   F
 0.7777970141999972  0.8888823413999987  0.1750651162999972   F   F   F
 0.2221375239702549  0.1108236384194744  0.2657001400775192   T   T   T
 0.5538450150951526  0.1095131094696231  0.2648837721616484   T   T   T
 0.8889445048430319  0.1090274766311578  0.2645677596331018   T   T   T
 0.2242068901455473  0.4451793931308817  0.2663897570801773   T   T   T
 0.5552506221723262  0.4443108394725988  0.2656473229574078   T   T   T
 0.8893721638800284  0.4460756912415831  0.2641928419378674   T   T   T
 0.2228258765026604  0.7771693752484282  0.2659593443576885   T   T   T
 0.5538708322168195  0.7772885041014707  0.2660576322194308   T   T   T
 0.8889343303881384  0.7777209264526380  0.2654570282682344   T   T   T
-0.0003289555853119  0.0002457014848329  0.3506051186187231   T   T   T
 0.3338314578165443 -0.0008273913146767  0.3501954245568780   T   T   T
 0.6701152097306753  0.0057977508861954  0.3499077571317304   T   T   T
 0.9928932049338628  0.3304678726635799  0.3476629385132990   T   T   T
 0.3355696877805055  0.3303691952865750  0.3528461434693643   T   T   T
 0.6703716961885422  0.3305174698880314  0.3488655271307697   T   T   T
 0.0001064177965198  0.6646029288281463  0.3503147932921035   T   T   T
 0.3329702395991189  0.6664923141241721  0.3549101549016222   T   T   T
 0.6665515438554770  0.6696537078962550  0.3508712602443770   T   T   T
 0.4496804987777677  0.5532233989202600  0.4107895599536002   T   T   T
 0.1389244463031845  0.3606218161921505  0.4581212788291053   T   T   T
 0.7415198015536109  0.5852251281331488  0.4586396278861144   T   T   T
```

8) Na_2O-Co(211)-1x3

```
Co(211)-3x3
   1.00000000000000
     6.0968000000000000    0.0000000000000000    0.0000000000000000
     0.0000000000000000    7.4669999999999996    0.0000000000000000
     0.0000000000000000    0.0000000000000000   23.6221999999999994
   Co   O    Na
   48    1     2
Selective dynamics
Direct
 0.0039768403096687  0.1666663988214765  0.9745822997011331   F   F   F
 0.0039765122687285  0.5000000000000000  0.9745822997011331   F   F   F
 0.0039768403096687  0.8333336011785235  0.9745822997011331   F   F   F
 0.3315888662905095  0.0000000000000000  0.0034278771663878   F   F   F
 0.3315855858811219  0.3333350743270387  0.0034291471581795   F   F   F
 0.3315855858811219  0.6666647917503710  0.0034291471581795   F   F   F
 0.6664853693741009  0.1666655952859273  0.0285661792720404   F   F   F
 0.6664884857630327  0.5000000000000000  0.0285654596100287   F   F   F
 0.6664853693741009  0.8333344047140727  0.0285661792720404   F   F   F
 0.9983825941477491  0.0000000000000000  0.0609119811025209   F   F   F
```

0.9983791497178913 0.3333345386366702 0.0609079171288016 F F F
0.9983791497178913 0.6666654613633298 0.0609079171288016 F F F
0.3314264860254568 0.1666650595955517 0.0918800111759310 F F F
0.3314281262301506 0.5000000000000000 0.0918805615056968 F F F
0.3314264860254568 0.8333349404044483 0.0918800111759310 F F F
0.6654751673008832 0.0000000000000000 0.1205095207050988 F F F
0.6654631938065876 0.3333330654881479 0.1205040597404121 F F F
0.6654631938065876 0.6666669345118521 0.1205040597404121 F F F
0.0009121178323070 0.1666669345118521 0.1526111031148716 F F F
0.0009114617504267 0.5000000000000000 0.1526112301140472 F F F
0.0009121178323070 0.8333330654881479 0.1526111031148716 F F F
0.3340214538774404 0.0000000000000000 0.1833969740329024 F F F
0.3340229300616713 0.3333327976429601 0.1833968470337197 F F F
0.3340229300616713 0.6666672023570399 0.1833968470337197 F F F
0.6655821287910602 0.1668431383189340 0.2117088250351775 T T T
0.6668572790929191 0.5001748255385742 0.2117210293961458 T T T
0.6656478898741726 0.8334977678343171 0.2117280225223900 T T T
0.9976654652466771 0.0002760802133924 0.2434013885829756 T T T
0.9990461964891442 0.3333576400088915 0.2431517729846123 T T T
0.9991068720503014 0.6669258733323830 0.2431345948961191 T T T
0.3322741382127590 0.1657073138076697 0.2748862694046491 T T T
0.3332729227843304 0.5002925476014702 0.2741535734974035 T T T
0.3324364872068558 0.8350908039651603 0.2749325767103811 T T T
0.6660352724136506 0.0004368406273403 0.3031945854837930 T T T
0.6677715423790579 0.3332722304922451 0.3025606624715578 T T T
0.6677199126723382 0.6674864880295392 0.3025741510549418 T T T
0.9995878652569509 0.1670625395948408 0.3351874023648010 T T T
-0.0007561895679957 0.5005315361135679 0.3354785858588274 T T T
0.9997046719551758 0.8340647390133386 0.3351816261628597 T T T
0.3290263867039529 0.0006104057561895 0.3693972950926733 T T T
0.3311108846346809 0.3326208677232209 0.3633912610284071 T T T
0.3312471216413790 0.6682728643536109 0.3634031042584279 T T T
0.6669684752085723 0.1665625385715724 0.3912602739867361 T T T
0.6621268316075702 0.5005881335914051 0.3900951564383917 T T T
0.6671220837383890 0.8344367243648314 0.3911912807950934 T T T
0.9879154868941545 0.0005794670418491 0.4213351610769437 T T T
0.9866982507998551 0.3369719419383915 0.4255181656644864 T T T
0.9858227194948890 0.6641003444936842 0.4253789963442483 T T T
0.1059669506924052 0.5012360443191954 0.4771498312023839 T T T
0.3787526831448976 0.7323050011145081 0.4844344581755369 T T T
0.3835036096373457 0.2778411376652029 0.4846225228807932 T T T

9) H-Co(111)-3x3

Co111_3X3
1.00000000000000
7.4587998390197754 0.0000000000000000 0.0000000000000000
-3.7294002959821051 6.4595099249780574 0.0000000000000000
-0.0000010106117090 -0.0000017504309440 23.1201000213622159
Co H
36 1
Selective dynamics
Direct
0.0000353225881327 0.9999648942106703 0.0907110695049838 F F F
0.3333557427970177 0.0000001793936022 0.0907175141137841 F F F
0.6666917648931943 0.9999938437795350 0.0907267268766958 F F F

0.0000062578975388 0.3333084372988537 0.0907267268766958   F   F   F
0.3333357007148621 0.3333426504206471 0.0907265971194704   F   F   F
0.6666945969032270 0.3333051862794676 0.0907313116319486   F   F   F
0.9999998791701969 0.6666444062796870 0.0907175141137841   F   F   F
0.3333385397113773 0.6666614354387903 0.0907302303217463   F   F   F
0.6666573639132167 0.6666642220269168 0.0907265971194704   F   F   F
0.1111175321760314 0.2222028177229376 0.1750651163386081   F   F   F
0.4444257597051688 0.2222195372577005 0.1750655488626904   F   F   F
0.7777781372906531 0.2222218594174734 0.1750735938105805   F   F   F
0.1111123692640206 0.5555522552209098 0.1750674087162309   F   F   F
0.4444440697289238 0.5555561254847916 0.1750719502190776   F   F   F
0.7777804782179913 0.5555742383122606 0.1750655488626904   F   F   F
0.1111199658994551 0.8888800192425492 0.1750494157144900   F   F   F
0.4444476042812084 0.8888872953412061 0.1750674087162309   F   F   F
0.7777970141657633 0.8888823414043898 0.1750651163386081   F   F   F
0.2224291914823709 0.1114860978844242 0.2654663925162335   T   T   T
0.5558656833659361 0.1112327459103788 0.2653033147277880   T   T   T
0.8887504041543466 0.1112492744758355 0.2652816691922229   T   T   T
0.2221893963104555 0.4443877503904330 0.2655908375963801   T   T   T
0.5556521774696990 0.4443477858388194 0.2655970907706153   T   T   T
0.8887672650646076 0.4441342206638560 0.2653033147277880   T   T   T
0.2223941094648475 0.7776057166172979 0.2654428983024547   T   T   T
0.5556123405712013 0.7778103591848184 0.2655908375963801   T   T   T
0.8885137127344428 0.7775707296764752 0.2654663925162335   T   T   T
-0.0000215697849555 0.0000214189168304 0.3494035012962458   T   T   T
0.3333626060572703 0.0000335932143061 0.3494121225901914   T   T   T
0.6673371618904517 0.0012292132406806 0.3494884600222356   T   T   T
0.9987706651408281 0.3326627396522843 0.3494884600222356   T   T   T
0.3329571874349466 0.3325087390401797 0.3511022364418594   T   T   T
0.6673557599760700 0.3326440652483155 0.3495047093800124   T   T   T
-0.0000334474763423 0.6666375947809595 0.3494121225901914   T   T   T
0.3328327348728022 0.6671674380287202 0.3510722523910971   T   T   T
0.6674912915028620 0.6670429447278637 0.3511022364418594   T   T   T
0.4442402352599193 0.5557600786825238 0.3933913343539523   T   T   T

10) H-PrO_2-Co(111)-3x3

Co111_3X3
1.00000000000000
7.4587998390197754   0.0000000000000000   0.0000000000000000
-3.7294002959821051   6.4595099249780574   0.0000000000000000
-0.0000010106117090   -0.0000017504309440   23.1201000213622159
Co   Pr   O   H
36   1   2   1
Selective dynamics
Direct
0.0000353225881327 0.9999648942106703 0.0907110695049838   F   F   F
0.3333557427970177 0.0000001793936022 0.0907175141137841   F   F   F
0.6666917648931943 0.9999938437795350 0.0907267268766958   F   F   F
0.0000062578975388 0.3333084372988537 0.0907267268766958   F   F   F
0.3333357007148621 0.3333426504206471 0.0907265971194704   F   F   F
0.6666945969032270 0.3333051862794676 0.0907313116319486   F   F   F
0.9999998791701969 0.6666444062796870 0.0907175141137841   F   F   F
0.3333385397113773 0.6666614354387903 0.0907302303217463   F   F   F
0.6666573639132167 0.6666642220269168 0.0907265971194704   F   F   F

0.1111175321760314 0.2222028177229376 0.1750651163386081   F   F   F
0.4444257597051688 0.2222195372577005 0.1750655488626904   F   F   F
0.7777781372906531 0.2222218594174734 0.1750735938105805   F   F   F
0.1111123692640206 0.5555522552209098 0.1750674087162309   F   F   F
0.4444440697289238 0.5555561254847916 0.1750719502190776   F   F   F
0.7777804782179913 0.5555742383122606 0.1750655488626904   F   F   F
0.1111199658994551 0.8888800192425492 0.1750494157144900   F   F   F
0.4444476042812084 0.8888872953412061 0.1750674087162309   F   F   F
0.7777970141657633 0.8888823414043898 0.1750651163386081   F   F   F
0.2191479633409761 0.1077986947864803 0.2644190925534561   T   T   T
0.5547020951815628 0.1110556478749043 0.2650369120607196   T   T   T
0.8888881379693437 0.1121111827377106 0.2657295298291154   T   T   T
0.2164033529507331 0.4457303361059829 0.2630829286001410   T   T   T
0.5581614066197580 0.4441259826017648 0.2633187266039544   T   T   T
0.8892466348925674 0.4448663300582069 0.2672400397372539   T   T   T
0.2182326166180671 0.7741645511320467 0.2666262824704302   T   T   T
0.5534671054439910 0.7763371945688036 0.2657150756983881   T   T   T
0.8890250925352465 0.7769019827857860 0.2664033998911977   T   T   T
0.9948437201046559 0.9959777563969873 0.3517382432137072   T   T   T
0.3359843862957707 0.9959822160505551 0.3498883544570784   T   T   T
0.6653541717899776 0.9986626018550370 0.3490953257136969   T   T   T
0.9935736476130420 0.3293737058484049 0.3540130100213965   T   T   T
0.3327135497530770 0.3383807152484827 0.3431273192806615   T   T   T
0.6629884243189098 0.3300592112767888 0.3518276733600922   T   T   T
-0.0043425160718574 0.6698529504151302 0.3568212378598039   T   T   T
0.3366925423771928 0.6676347293078732 0.3530429350671345   T   T   T
0.6642444268131792 0.6639343340313545 0.3497863560168522   T   T   T
0.3320186768621581 0.2293676480123676 0.4494713401881902   T   T   T
0.2271885861212594 0.4392906274623051 0.4097660960665513   T   T   T
0.1274272498665911 0.9132561936378002 0.4135729059342196   T   T   T
0.7540335426856951 0.5326592052211282 0.3972744612015613   T   T   T

11) H-Na_2O-Co(111)-3x3

Co O Na H
1.00000000000000
7.4587998390197754   0.0000000000000000   0.0000000000000000
-3.7294002959821051   6.4595099249780574   0.0000000000000000
-0.0000010106117090   -0.0000017504309440   23.1201000213622159
Co   O   Na   H
36   1   2   1
Selective dynamics
Direct
0.0000353225881327 0.9999648942106703 0.0907110695049766   F   F   F
0.3333557427970177 0.0000001793936022 0.0907175141137770   F   F   F
0.6666917648932014 0.9999938437795421 0.0907267268767029   F   F   F
0.0000062578975388 0.3333084372988466 0.0907267268767029   F   F   F
0.3333357007148621 0.3333426504206471 0.0907265971194704   F   F   F
0.6666945969032270 0.3333051862794676 0.0907313116319486   F   F   F
0.9999998791701969 0.6666444062796870 0.0907175141137770   F   F   F
0.3333385397113773 0.6666614354387903 0.0907302303217534   F   F   F
0.6666573639132167 0.6666642220269168 0.0907265971194704   F   F   F
0.1111175321760314 0.2222028177229376 0.1750651163386081   F   F   F
0.4444257597051688 0.2222195372577005 0.1750655488626904   F   F   F

```
0.7777781372906531  0.2222218594174734  0.1750735938105805   F   F   F
0.1111123692640206  0.5555522552209098  0.1750674087162309   F   F   F
0.4444440697289167  0.5555561254847916  0.1750719502190776   F   F   F
0.7777804782179913  0.5555742383122606  0.1750655488626904   F   F   F
0.1111199658994622  0.8888800192425492  0.1750494157144900   F   F   F
0.4444476042812084  0.8888872953412132  0.1750674087162309   F   F   F
0.7777970141657633  0.8888823414043898  0.1750651163386081   F   F   F
0.2220336880034814  0.1103178370011476  0.2655455311724691   T   T   T
0.5545196097401827  0.1099351487842747  0.2652706876494403   T   T   T
0.8888093143999289  0.1084257349884131  0.2644440007831217   T   T   T
0.2242505714226629  0.4452187157969892  0.2657896014704487   T   T   T
0.5554704360603749  0.4439521219343788  0.2656342208764292   T   T   T
0.8889372104003841  0.4465409979669758  0.2639444387473621   T   T   T
0.2227663146891153  0.7774438039533232  0.2658277166235924   T   T   T
0.5541245980090254  0.7776000584814576  0.2660095512679364   T   T   T
0.8888663813522090  0.7776800842128224  0.2650154902384230   T   T   T
0.9989399003128685 -0.0006280610299482  0.3497974240970271   T   T   T
0.3315620622894667  0.9981871764899126  0.3510007538373205   T   T   T
0.6705990469366677  0.0052990926475594  0.3511806651809586   T   T   T
0.9933789854474215  0.3308128488377471  0.3468777636870993   T   T   T
0.3332618234031243  0.3310121940015572  0.3521696650103656   T   T   T
0.6708812817444638  0.3305595303049425  0.3503563639212385   T   T   T
-0.0006316172099991  0.6644525754182483  0.3493852256552255   T   T   T
0.3307646249012560  0.6641973788539290  0.3543583296509578   T   T   T
0.6664458882876343  0.6694040445299623  0.3501074430284503   T   T   T
0.4514718611410282  0.5540878727318208  0.4102626316358868   T   T   T
0.1409595328448180  0.3595239767581421  0.4572753099306215   T   T   T
0.7446096333102815  0.5895126693954520  0.4576938552972054   T   T   T
0.5531782915113247  0.1122612188743686  0.3940216082450360   T   T   T
```

12) CO-Co(111)-3x3

```
Co O C
   1.00000000000000
     7.4587998390197754    0.0000000000000000    0.0000000000000000
    -3.7294002959821051    6.4595099249780574    0.0000000000000000
    -0.0000010106117090   -0.0000017504309440   23.1201000213622159
   Co   O    C
   36    1    1
Selective dynamics
Direct
 0.0000353225881327  0.9999648942106703  0.0907110695049766   F   F   F
 0.3333557427970177  0.0000001793936022  0.0907175141137770   F   F   F
 0.6666917648932014  0.9999938437795421  0.0907267268767029   F   F   F
 0.0000062578975388  0.3333084372988466  0.0907267268767029   F   F   F
 0.3333357007148621  0.3333426504206471  0.0907265971194704   F   F   F
 0.6666945969032270  0.3333051862794676  0.0907313116319486   F   F   F
 0.9999998791701969  0.6666444062796870  0.0907175141137770   F   F   F
 0.3333385397113773  0.6666614354387903  0.0907302303217534   F   F   F
 0.6666573639132167  0.6666642220269168  0.0907265971194704   F   F   F
 0.1111175321760314  0.2222028177229376  0.1750651163386081   F   F   F
 0.4444257597051688  0.2222195372577005  0.1750655488626904   F   F   F
 0.7777781372906531  0.2222218594174734  0.1750735938105805   F   F   F
 0.1111123692640206  0.5555522552209098  0.1750674087162309   F   F   F
```

```
0.4444440697289167  0.5555561254847916  0.1750719502190776   F   F   F
0.7777804782179913  0.5555742383122606  0.1750655488626904   F   F   F
0.1111199658994622  0.8888800192425492  0.1750494157144900   F   F   F
0.4444476042812084  0.8888872953412132  0.1750674087162309   F   F   F
0.7777970141657633  0.8888823414043898  0.1750651163386081   F   F   F
0.2221036974284626  0.1108669016928796  0.2650251033425200   T   T   T
0.5554764457769095  0.1108803365706740  0.2650265186596194   T   T   T
0.8889069478731588  0.1110929363341530  0.2649879941267082   T   T   T
0.2220873552126460  0.4445307453312975  0.2650259249668122   T   T   T
0.5555638123215225  0.4444410809561343  0.2663685461318546   T   T   T
0.8891219341067715  0.4445257665880609  0.2650253595715871   T   T   T
0.2222231281630335  0.7777782381542686  0.2650931804688371   T   T   T
0.5554673009132628  0.7779130286603022  0.2650275140874026   T   T   T
0.8891326671982605  0.7779012885493886  0.2650259637089440   T   T   T
-0.0001400996809232  0.0001400943895795  0.3486062486709154   T   T   T
0.3322933912777389  0.9978779787815756  0.3493625904538315   T   T   T
0.6669540885494030  0.0001441053840270  0.3486042788051772   T   T   T
-0.0001456771256583  0.3330430722955081  0.3486032654043956   T   T   T
0.3311943308095472  0.3322629339010165  0.3521607889053422   T   T   T
0.6677402652691588  0.3322586939784946  0.3521635815894781   T   T   T
0.0021166883284463  0.6677085770723037  0.3493629441061497   T   T   T
0.3322750211082653  0.6677059568972596  0.3493655412845165   T   T   T
0.6677389575800685  0.6688122093019238  0.3521631872160244   T   T   T
0.5555440019058292  0.4444667002070210  0.4616508139109228   T   T   T
0.5555585436222646  0.4444471693398092  0.4098158261853002   T   T   T
```

13) CO-PrO_2-Co(111)-3x3

```
Co Pr O C
  1.00000000000000
    7.4587998390197754    0.0000000000000000    0.0000000000000000
   -3.7294002959821051    6.4595099249780574    0.0000000000000000
   -0.0000010106117090   -0.0000017504309440   23.1201000213622159
  Co   Pr   O    C
   36    1    3    1
Selective dynamics
Direct
0.0000353225881327  0.9999648942106703  0.0907110695049766   F   F   F
0.3333557427970177  0.0000001793936022  0.0907175141137770   F   F   F
0.6666917648932014  0.9999938437795421  0.0907267268767029   F   F   F
0.0000062578975388  0.3333084372988466  0.0907267268767029   F   F   F
0.3333357007148621  0.3333426504206471  0.0907265971194704   F   F   F
0.6666945969032270  0.3333051862794676  0.0907313116319486   F   F   F
0.9999998791701969  0.6666444062796870  0.0907175141137770   F   F   F
0.3333385397113773  0.6666614354387903  0.0907302303217534   F   F   F
0.6666573639132167  0.6666642220269168  0.0907265971194704   F   F   F
0.1111175321760314  0.2222028177229376  0.1750651163386081   F   F   F
0.4444257597051688  0.2222195372577005  0.1750655488626904   F   F   F
0.7777781372906531  0.2222218594174734  0.1750735938105805   F   F   F
0.1111123692640206  0.5555522552209098  0.1750674087162309   F   F   F
0.4444440697289167  0.5555561254847916  0.1750719502190776   F   F   F
0.7777804782179913  0.5555742383122606  0.1750655488626904   F   F   F
0.1111199658994622  0.8888800192425492  0.1750494157144900   F   F   F
0.4444476042812084  0.8888872953412132  0.1750674087162309   F   F   F
```

0.7777970141657633 0.8888823414043898 0.1750651163386081 F F F
0.2246204323930829 0.1133391550036773 0.2658276030001413 T T T
0.5541713353262103 0.1100014613146186 0.2640123926539331 T T T
0.8898993497032982 0.1123094112548517 0.2645471850347881 T T T
0.2210885426706755 0.4458598360629237 0.2645794074443593 T T T
0.5576773693763727 0.4451470709112306 0.2649468187374439 T T T
0.8913436892489106 0.4461092891940112 0.2650190181547976 T T T
0.2215643484408887 0.7778075551209848 0.2666735642558533 T T T
0.5536610888178682 0.7764350927744694 0.2649301268503573 T T T
0.8905342548718762 0.7764208964470659 0.2649190301361435 T T T
0.9939195788799134 0.9955037388540243 0.3490326042370613 T T T
0.3354125956071713 0.9979449343467273 0.3522763554554164 T T T
0.6653548787904541 0.9982830947628608 0.3479013818888871 T T T
0.9904870576075824 0.3281736009348064 0.3511469355499036 T T T
0.3412677865475350 0.3416720471603109 0.3519131903318257 T T T
0.6688227605838196 0.3295892972094258 0.3506555156975117 T T T
0.9986773813777425 0.6704056590253886 0.3530409060552684 T T T
0.3373299384665452 0.6726091769618736 0.3518221686071695 T T T
0.6689453437808269 0.6694519349845311 0.3518799883867835 T T T
0.1956288523832697 0.1891781418754368 0.4582191725521584 T T T
0.2009808186302912 0.4506353041051009 0.4088110330178136 T T T
0.1116329003371559 0.8936502927781822 0.4113691139093410 T T T
0.5624823659074319 0.4334785591848711 0.4617079351304048 T T T
0.5761598656011695 0.4460733485274896 0.4065385691988560 T T T

14) CO-Na_2O-Co(111)-3x3

Co O Na C
1.00000000000000
7.4587998390197754 0.0000000000000000 0.0000000000000000
-3.7294002959821051 6.4595099249780574 0.0000000000000000
-0.0000010106117090 -0.0000017504309440 23.1201000213622159
Co O Na C
36 2 2 1
Selective dynamics
Direct
0.0000353225881327 0.9999648942106703 0.0907110695049766 F F F
0.3333557427970177 0.0000001793936022 0.0907175141137770 F F F
0.6666917648932014 0.9999938437795421 0.0907267268767029 F F F
0.0000062578975388 0.3333084372988466 0.0907267268767029 F F F
0.3333357007148621 0.3333426504206471 0.0907265971194704 F F F
0.6666945969032270 0.3333051862794676 0.0907313116319486 F F F
0.9999998791701969 0.6666444062796870 0.0907175141137770 F F F
0.3333385397113773 0.6666614354387903 0.0907302303217534 F F F
0.6666573639132167 0.6666642220269168 0.0907265971194704 F F F
0.1111175321760314 0.2222028177229376 0.1750651163386081 F F F
0.4444257597051688 0.2222195372577005 0.1750655488626904 F F F
0.7777781372906531 0.2222218594174734 0.1750735938105805 F F F
0.1111123692640206 0.5555522552209098 0.1750674087162309 F F F
0.4444440697289167 0.5555561254847916 0.1750719502190776 F F F
0.7777804782179913 0.5555742383122606 0.1750655488626904 F F F
0.1111199658994622 0.8888800192425492 0.1750494157144900 F F F
0.4444476042812084 0.8888872953412132 0.1750674087162309 F F F
0.7777970141657633 0.8888823414043898 0.1750651163386081 F F F

```
 0.2226599004348977  0.1121061546981159  0.2653456600965885   T   T   T
 0.5547213267799483  0.1105638157776499  0.2646009590223369   T   T   T
 0.8888696872656416  0.1102462204533707  0.2653687238078457   T   T   T
 0.2240607022663711  0.4443289132927175  0.2661265318169739   T   T   T
 0.5536989755726143  0.4427655013792817  0.2654364956850360   T   T   T
 0.8886873497797644  0.4459019154056134  0.2637844648571075   T   T   T
 0.2224125151728259  0.7773518841563372  0.2651039622049164   T   T   T
 0.5553685795652969  0.7770575004983036  0.2653480976954286   T   T   T
 0.8886110753474302  0.7776884573663773  0.2647905801946258   T   T   T
 0.0016099844740726 -0.0009435951152835  0.3519168862307597   T   T   T
 0.3334868960320312  0.9993515959305129  0.3488473430394419   T   T   T
 0.6671829757792229  0.0036906634778825  0.3521825937468550   T   T   T
 0.9949314558822192  0.3332568193041241  0.3503182918049295   T   T   T
 0.3389614803561818  0.3341391424361143  0.3539099499186197   T   T   T
 0.6691963246859348  0.3321083970320101  0.3486177392686298   T   T   T
 0.0015012880888389  0.6660005678259459  0.3493023254699507   T   T   T
 0.3358397186949341  0.6701436224220975  0.3522704955085173   T   T   T
 0.6661628261162766  0.6606347645985137  0.3495311303091539   T   T   T
 0.4532359436579617  0.5638892597075303  0.4103487418522269   T   T   T
 0.8963756332869324  0.1156894208806303  0.4616256752648365   T   T   T
 0.1564103136326972  0.4641062300338658  0.4611549521352474   T   T   T
 0.7644123521711568  0.7602125264291260  0.4610694240340514   T   T   T
 0.8952425055837765  0.1199411817164053  0.4063412535039359   T   T   T
```

15) H-Co(211)-1x3

```
Co(211)-3x3
   1.00000000000000
     6.0968000000000000    0.0000000000000000    0.0000000000000000
     0.0000000000000000    7.4669999999999996    0.0000000000000000
     0.0000000000000000    0.0000000000000000   23.6221999999999994
   Co   H
    48     1
Selective dynamics
Direct
 0.0039768403096687  0.1666663988214836  0.9745822997011260   F   F   F
 0.0039765122687285  0.5000000000000000  0.9745820457027676   F   F   F
 0.0039768403096687  0.8333336011785164  0.9745822997011260   F   F   F
 0.3315888662905095  0.0000000000000000  0.0034278771663949   F   F   F
 0.3315855858811148  0.3333350743270387  0.0034291471581795   F   F   F
 0.3315855858811148  0.6666647917503710  0.0034291471581795   F   F   F
 0.6664853693741009  0.1666655952859273  0.0285661792720404   F   F   F
 0.6664884857630256  0.5000000000000000  0.0285654596100287   F   F   F
 0.6664853693741009  0.8333344047140727  0.0285661792720404   F   F   F
 0.9983825941477491  0.0000000000000000  0.0609119811025209   F   F   F
 0.9983791497178842  0.3333345386366702  0.0609079171288016   F   F   F
 0.9983791497178842  0.6666654613633298  0.0609079171288016   F   F   F
 0.3314264860254568  0.1666650595955517  0.0918800111759310   F   F   F
 0.3314281262301506  0.5000000000000000  0.0918805615057039   F   F   F
 0.3314264860254568  0.8333349404044483  0.0918800111759310   F   F   F
 0.6654751673008761  0.0000000000000000  0.1205095207050988   F   F   F
 0.6654631938065876  0.3333330654881479  0.1205040597404121   F   F   F
 0.6654631938065876  0.6666669345118521  0.1205040597404121   F   F   F
 0.0009121178323070  0.1666669345118521  0.1526111031148645   F   F   F
```

0.0009114617504267 0.5000000000000000 0.1526112301140472 F F F
0.0009121178323070 0.8333330654881479 0.1526111031148645 F F F
0.3340214538774404 0.0000000000000000 0.1833969740329024 F F F
0.3340229300616713 0.3333327976429601 0.1833968470337197 F F F
0.3340229300616713 0.6666672023570399 0.1833968470337197 F F F
0.6659075478749005 0.1667425763836577 0.2120608371868066 T T T
0.6654222140421522 0.4999804391611274 0.2120747318145073 T T T
0.6659060550970877 0.8332131059645395 0.2120587887988211 T T T
0.9993331675290085 0.0000025241197139 0.2425556302596794 T T T
0.9982086379092424 0.3333517232772652 0.2426471252536520 T T T
0.9982071957486578 0.6666102478270592 0.2426436655430493 T T T
0.3346643569559713 0.1666807671243641 0.2747684054115404 T T T
0.3337915709990743 0.4999831682990099 0.2749892740702782 T T T
0.3346772120115538 0.8332931158111589 0.2747723822142937 T T T
0.6691866228884117 -0.0000409899969991 0.3033163937320021 T T T
0.6674888440355041 0.3339969845417470 0.3037061707485622 T T T
0.6674732661394517 0.6659386861055385 0.3037262996008369 T T T
0.0018387078492426 0.1662691251799880 0.3340110045415151 T T T
0.0003819898157567 0.4999423300563042 0.3343458796982838 T T T
0.0018506325352221 0.8336872350016312 0.3339937148428434 T T T
0.3328946935284433 -0.0000211635235944 0.3663192053489376 T T T
0.3334100934074803 0.3332126409893287 0.3666987885690342 T T T
0.3334141418636439 0.6667322570659311 0.3667085494275082 T T T
0.6679883450717899 0.1656694592774273 0.3916581283242920 T T T
0.6676555225948456 0.4999024987587957 0.3939746017344007 T T T
0.6679396612973107 0.8341522832419273 0.3916580213788689 T T T
0.9974811386156500 -0.0001728302591857 0.4198030710211847 T T T
0.9952222112097231 0.3318765641747736 0.4210868539526317 T T T
0.9951967524766275 0.6679774903050043 0.4210847767529043 T T T
0.8464472131059547 0.4998689792134908 0.4547233256787429 T T T

16) H-PrO_2-Co(211)-1x3

Co(211)-3x3
1.00000000000000
6.0968000000000000 0.0000000000000000 0.0000000000000000
0.0000000000000000 7.4669999999999996 0.0000000000000000
0.0000000000000000 0.0000000000000000 23.6221999999999994
Co Pr O H
48 1 2 1
Selective dynamics
Direct
0.0039768403096687 0.1666663988214836 0.9745822997011260 F F F
0.0039765122687285 0.5000000000000000 0.9745819610366553 F F F
0.0039768403096687 0.8333336011785164 0.9745822997011260 F F F
0.3315888662905095 0.0000000000000000 0.0034278771663949 F F F
0.3315855858811148 0.3333350743270387 0.0034291471581795 F F F
0.3315855858811148 0.6666647917503710 0.0034291471581795 F F F
0.6664853693741009 0.1666655952859273 0.0285661792720404 F F F
0.6664884857630256 0.5000000000000000 0.0285654596100287 F F F
0.6664853693741009 0.8333344047140727 0.0285661792720404 F F F
0.9983825941477491 0.0000000000000000 0.0609119811025209 F F F
0.9983791497178842 0.3333345386366702 0.0609079171288016 F F F
0.9983791497178842 0.6666654613633298 0.0609079171288016 F F F

0.3314264860254568 0.1666650595955517 0.0918800111759310 F F F
0.3314281262301506 0.5000000000000000 0.0918805615057039 F F F
0.3314264860254568 0.8333349404044483 0.0918800111759310 F F F
0.6654751673008761 0.0000000000000000 0.1205095207050988 F F F
0.6654631938065876 0.3333330654881479 0.1205040597404121 F F F
0.6654631938065876 0.6666669345118521 0.1205040597404121 F F F
0.0009121178323070 0.1666669345118521 0.1526111031148645 F F F
0.0009114617504267 0.5000000000000000 0.1526112301140472 F F F
0.0009121178323070 0.8333330654881479 0.1526111031148645 F F F
0.3340214538774404 0.0000000000000000 0.1833969740329024 F F F
0.3340229300616713 0.3333327976429601 0.1833968470337197 F F F
0.3340229300616713 0.6666672023570399 0.1833968470337197 F F F
0.6652067687614618 0.1660260622257642 0.2117581642129120 T T T
0.6666112268661125 0.4996458227400032 0.2119678488169355 T T T
0.6659634727998642 0.8339010246993521 0.2119254963882900 T T T
0.9965483728025958 0.0006530877129741 0.2431953111975689 T T T
-0.0001394948403334 0.3335991062127469 0.2428770173288116 T T T
-0.0000214143935374 0.6659671088440241 0.2427545259357554 T T T
0.3325994222227879 0.1666639429315361 0.2755584668379062 T T T
0.3357474430119203 0.4982792723513034 0.2754832091969216 T T T
0.3319361501426492 0.8343230961211812 0.2739344005498196 T T T
0.6646240618638403 0.0004562453873235 0.3035652748013982 T T T
0.6700510693230065 0.3287759840297700 0.3020667889191338 T T T
0.6658343915442785 0.6679099398123817 0.3023237497390294 T T T
0.0002688444054577 0.1678067225182660 0.3347068440706438 T T T
0.0044527745431080 0.4981992004458877 0.3347601341413541 T T T
-0.0031084668870038 0.8312950923828641 0.3344620382393557 T T T
0.3286362651398992 -0.0042159488229669 0.3660650358483465 T T T
0.3268795551249948 0.3304142199567579 0.3692866422545333 T T T
0.3277139890133021 0.6735579372191476 0.3641969911948855 T T T
0.6724902053901375 0.1621674089924025 0.3956328097408930 T T T
0.6713675508853488 0.4992180128120329 0.3870350954848259 T T T
0.6634329175548970 0.8348962500450262 0.3932780466450750 T T T
0.9958715053244804 -0.0033237241002427 0.4204812411721088 T T T
-0.0000670470059774 0.3370599588476814 0.4259017916959014 T T T
0.9764519018850490 0.6678558346326093 0.4249906658215404 T T T
0.4035714243168471 0.5905800902576853 0.4824582666174612 T T T
0.5193082485350469 0.3394406089636171 0.4411547837462604 T T T
0.0565943590987361 0.5011089377371946 0.4852147774507706 T T T
0.8176659049862471 0.8417249953457976 0.4550520341413495 T T T

17) H-Na_2O-Co(211)-1x3

Co(211)-3x3
1.00000000000000
6.0968000000000000 0.0000000000000000 0.0000000000000000
0.0000000000000000 7.4669999999999996 0.0000000000000000
0.0000000000000000 0.0000000000000000 23.6221999999999994
Co O Na H
48 1 2 1
Selective dynamics
Direct
0.0039768403096687 0.1666663988214765 0.9745822997011331 F F F
0.0039765122687285 0.5000000000000000 0.9745822997011331 F F F
0.0039768403096687 0.8333336011785235 0.9745822997011331 F F F

0.3315888662905095	0.0000000000000000	0.0034278771663878	F	F	F
0.3315855858811219	0.3333350743270387	0.0034291471581795	F	F	F
0.3315855858811219	0.6666647917503710	0.0034291471581795	F	F	F
0.6664853693741009	0.1666655952859273	0.0285661792720404	F	F	F
0.6664884857630327	0.5000000000000000	0.0285654596100287	F	F	F
0.6664853693741009	0.8333344047140727	0.0285661792720404	F	F	F
0.9983825941477491	0.0000000000000000	0.0609119811025209	F	F	F
0.9983791497178913	0.3333345386366702	0.0609079171288016	F	F	F
0.9983791497178913	0.6666654613633298	0.0609079171288016	F	F	F
0.3314264860254568	0.1666650595955517	0.0918800111759310	F	F	F
0.3314281262301506	0.5000000000000000	0.0918805615056968	F	F	F
0.3314264860254568	0.8333349404044483	0.0918800111759310	F	F	F
0.6654751673008832	0.0000000000000000	0.1205095207050988	F	F	F
0.6654631938065876	0.3333330654881479	0.1205040597404121	F	F	F
0.6654631938065876	0.6666669345118521	0.1205040597404121	F	F	F
0.0009121178323070	0.1666669345118521	0.1526111031148716	F	F	F
0.0009114617504267	0.5000000000000000	0.1526112301140472	F	F	F
0.0009121178323070	0.8333330654881479	0.1526111031148716	F	F	F
0.3340214538774404	0.0000000000000000	0.1833969740329024	F	F	F
0.3340229300616713	0.3333327976429601	0.1833968470337197	F	F	F
0.3340229300616713	0.6666672023570399	0.1833968470337197	F	F	F
0.6659674072033428	0.1664263389120203	0.2117668895214391	T	T	T
0.6667693936345407	0.5002760327314161	0.2118509531235440	T	T	T
0.6653433747384949	0.8334116784821781	0.2118960691086768	T	T	T
0.9973997909551519	-0.0003122570896731	0.2433806437166484	T	T	T
0.9995481227378127	0.3336027187832307	0.2429975203508628	T	T	T
0.9985525395186134	0.6666895449455533	0.2432959106060703	T	T	T
0.3328580974270715	0.1655785705700051	0.2746161923291268	T	T	T
0.3332241377078415	0.5002930771315897	0.2742088353183476	T	T	T
0.3315131961132983	0.8347246966246131	0.2751004349187668	T	T	T
0.6653396770328229	-0.0008081309944815	0.3033598937086599	T	T	T
0.6679843797271969	0.3331835352745552	0.3023765102583033	T	T	T
0.6659635308600181	0.6680648032882724	0.3031725694016482	T	T	T
0.0000400588793651	0.1670658812229724	0.3342563432456641	T	T	T
0.9994525148598531	0.5001366688914094	0.3353904551737359	T	T	T
-0.0018583022633239	0.8327100540194835	0.3354567905052871	T	T	T
0.3295105437939897	0.0006240829150966	0.3687839288582370	T	T	T
0.3305647794254619	0.3319350656628934	0.3631585073860314	T	T	T
0.3309203296335538	0.6681737108801794	0.3633547672211947	T	T	T
0.6676872512229978	0.1677214065306828	0.3906954619114150	T	T	T
0.6621621028227725	0.4991538806348371	0.3899526772605293	T	T	T
0.6657119782042966	0.8354398748009635	0.3936715376351596	T	T	T
0.9898024429791771	0.0037537819084640	0.4214428578565002	T	T	T
0.9918042048101675	0.3348655085854445	0.4246363479335738	T	T	T
0.9798836401425487	0.6597002625489501	0.4272063463157648	T	T	T
0.1077746489631742	0.4930348805290349	0.4773843298196017	T	T	T
0.3731510549304654	0.7261266000961475	0.4845517654860206	T	T	T
0.3824550594976283	0.2686625998102146	0.4844913970343471	T	T	T
0.8337157486039309	0.8396497384614255	0.4545634632904084	T	T	T

18) CO-Co(211)-1x3

Co(211)-3x3
1.0000000000000000

```
     6.0968000000000000    0.0000000000000000    0.0000000000000000
     0.0000000000000000    7.4669999999999996    0.0000000000000000
     0.0000000000000000    0.0000000000000000   23.6221999999999994
   Co   C    O
    48     1     1
Selective dynamics
Direct
  0.0039768403096687  0.1666663988214836  0.9745822997011260   F   F   F
  0.0039765122687285  0.5000000000000000  0.9745819610366553   F   F   F
  0.0039768403096687  0.8333336011785164  0.9745822997011260   F   F   F
  0.3315888662905095  0.0000000000000000  0.0034278771663949   F   F   F
  0.3315855858811148  0.3333350743270387  0.0034291471581795   F   F   F
  0.3315855858811148  0.6666647917503710  0.0034291471581795   F   F   F
  0.6664853693741009  0.1666655952859273  0.0285661792720404   F   F   F
  0.6664884857630256  0.5000000000000000  0.0285654596100287   F   F   F
  0.6664853693741009  0.8333344047140727  0.0285661792720404   F   F   F
  0.9983825941477491  0.0000000000000000  0.0609119811025209   F   F   F
  0.9983791497178842  0.3333345386366702  0.0609079171288016   F   F   F
  0.9983791497178842  0.6666654613633298  0.0609079171288016   F   F   F
  0.3314264860254568  0.1666650595955517  0.0918800111759310   F   F   F
  0.3314281262301506  0.5000000000000000  0.0918805615057039   F   F   F
  0.3314264860254568  0.8333349404044483  0.0918800111759310   F   F   F
  0.6654751673008761  0.0000000000000000  0.1205095207050988   F   F   F
  0.6654631938065876  0.3333330654881479  0.1205040597404121   F   F   F
  0.6654631938065876  0.6666669345118521  0.1205040597404121   F   F   F
  0.0009121178323070  0.1666669345118521  0.1526111031148645   F   F   F
  0.0009114617504267  0.5000000000000000  0.1526112301140472   F   F   F
  0.0009121178323070  0.8333330654881479  0.1526111031148645   F   F   F
  0.3340214538774404  0.0000000000000000  0.1833969740329024   F   F   F
  0.3340229300616713  0.3333327976429601  0.1833968470337197   F   F   F
  0.3340229300616713  0.6666672023570399  0.1833968470337197   F   F   F
  0.6661549991465248  0.1671088045642204  0.2121370482318834   T   T   T
  0.6652657502845153  0.5000015103498405  0.2121330654994987   T   T   T
  0.6661124607590213  0.8328841079110644  0.2121329283428572   T   T   T
  0.9994160749341340  0.0000052536044698  0.2426842257032323   T   T   T
  0.9985746210866836  0.3340245771292583  0.2426360101780421   T   T   T
  0.9985342947834118  0.6659424189576791  0.2426283974096319   T   T   T
  0.3343453746893962  0.1669266906403577  0.2746075407314732   T   T   T
  0.3333483841401463  0.4999846212555771  0.2753018031418745   T   T   T
  0.3343338649606651  0.8330155988231027  0.2746101084265001   T   T   T
  0.6682658596518518 -0.0000269536024985  0.3032248522666768   T   T   T
  0.6671068968686519  0.3335707328329204  0.3038066872712201   T   T   T
  0.6671364186720850  0.6663448520771074  0.3038149424995706   T   T   T
  0.0018695158547401  0.1659493767865665  0.3331735140198635   T   T   T
 -0.0008970692702314  0.4999835214599412  0.3352222951670533   T   T   T
  0.0019135053750565  0.8340723162933716  0.3331646005911059   T   T   T
  0.3324077884408947 -0.0000152370560420  0.3661302008868556   T   T   T
  0.3344893868202183  0.3334477335242045  0.3662386227600017   T   T   T
  0.3344707664866270  0.6664445859190905  0.3662489140089750   T   T   T
  0.6680993464519451  0.1656058362065947  0.3913286873459751   T   T   T
  0.6659113234414806  0.4999704824390104  0.3951486098969861   T   T   T
  0.6681063797376795  0.8343574477438920  0.3913235367892759   T   T   T
  0.9977770183830449 -0.0000074470223833  0.4189927122397781   T   T   T
 -0.0060628242549301  0.3290503972765261  0.4219669693703065   T   T   T
 -0.0061290600394377  0.6709379384791161  0.4219736221732861   T   T   T
```

```
 0.8437213107813394  0.4999488573397495  0.4696933805210059   T   T   T
 0.7858516594498068  0.4998363955114090  0.5181969527513248   T   T   T
```

19) CO-PrO_2-Co(211)-1x3

```
Co(211)-3x3
   1.00000000000000
     6.0968000000000000    0.0000000000000000    0.0000000000000000
     0.0000000000000000    7.4669999999999996    0.0000000000000000
     0.0000000000000000    0.0000000000000000   23.6221999999999994
   Co   Pr   O    C
    48     1     3     1
Selective dynamics
Direct
  0.0039768403096687  0.1666663988214765  0.9745822997011331   F   F   F
  0.0039765122687285  0.5000000000000000  0.9745822997011331   F   F   F
  0.0039768403096687  0.8333336011785235  0.9745822997011331   F   F   F
  0.3315888662905095  0.0000000000000000  0.0034278771663878   F   F   F
  0.3315855858811219  0.3333350743270387  0.0034291471581795   F   F   F
  0.3315855858811219  0.6666647917503710  0.0034291471581795   F   F   F
  0.6664853693741009  0.1666655952859273  0.0285661792720404   F   F   F
  0.6664884857630327  0.5000000000000000  0.0285654596100287   F   F   F
  0.6664853693741009  0.8333344047140727  0.0285661792720404   F   F   F
  0.9983825941477491  0.0000000000000000  0.0609119811025209   F   F   F
  0.9983791497178913  0.3333345386366702  0.0609079171288016   F   F   F
  0.9983791497178913  0.6666654613633298  0.0609079171288016   F   F   F
  0.3314264860254568  0.1666650595955517  0.0918800111759310   F   F   F
  0.3314281262301506  0.5000000000000000  0.0918805615056968   F   F   F
  0.3314264860254568  0.8333349404044483  0.0918800111759310   F   F   F
  0.6654751673008832  0.0000000000000000  0.1205095207050988   F   F   F
  0.6654631938065876  0.3333330654881479  0.1205040597404121   F   F   F
  0.6654631938065876  0.6666669345118521  0.1205040597404121   F   F   F
  0.0009121178323070  0.1666669345118521  0.1526111031148716   F   F   F
  0.0009114617504267  0.5000000000000000  0.1526112301140472   F   F   F
  0.0009121178323070  0.8333330654881479  0.1526111031148716   F   F   F
  0.3340214538774404  0.0000000000000000  0.1833969740329024   F   F   F
  0.3340229300616713  0.3333327976429601  0.1833968470337197   F   F   F
  0.3340229300616713  0.6666672023570399  0.1833968470337197   F   F   F
  0.6653613728124529  0.1664578260424591  0.2117980719468279   T   T   T
  0.6666586309066196  0.5000313797493514  0.2119408757669775   T   T   T
  0.6659914870899454  0.8341629071782054  0.2119018324888515   T   T   T
  0.9974934696656351  0.0004976302811139  0.2433895034643271   T   T   T
 -0.0002633123707830  0.3337693069221044  0.2433310962050885   T   T   T
  0.0005911106266107  0.6668874065175531  0.2430092377945242   T   T   T
  0.3323331229694900  0.1673259507555524  0.2755509830768902   T   T   T
  0.3355354171547904  0.4991583807736408  0.2752810473085250   T   T   T
  0.3323926484615387  0.8349771752551072  0.2742738062374915   T   T   T
  0.6656360126150809  0.0020555510077673  0.3030445174250208   T   T   T
  0.6683208220882650  0.3299632273938333  0.3022326110535698   T   T   T
  0.6661856088351116  0.6675800086966346  0.3017751356461609   T   T   T
 -0.0002903970610149  0.1689617938794409  0.3346053250353859   T   T   T
  0.0039700054823608  0.4995107179755596  0.3342867432952809   T   T   T
  0.9970602132120677  0.8336056345382030  0.3356768295778463   T   T   T
  0.3282651795382773  0.9974178354674614  0.3656643359298637   T   T   T
```

```
  0.3273469268939008  0.3330144894485049  0.3691608229220192   T   T   T
  0.3279534198033027  0.6753140657828917  0.3640657939966930   T   T   T
  0.6703289490984979  0.1641818773280594  0.3950580129608196   T   T   T
  0.6719650682442321  0.4998072543593880  0.3867644858655807   T   T   T
  0.6597528975219376  0.8344673572543855  0.3923723650380088   T   T   T
  0.9859509994381287 -0.0002994464043558  0.4228900076596769   T   T   T
  0.0029436661185110  0.3351652724672987  0.4258953541330976   T   T   T
  0.9797222719183487  0.6663610553999267  0.4266669529553186   T   T   T
  0.4173386133427402  0.5935132845643734  0.4855392530519371   T   T   T
  0.5193082221195895  0.3417149760160189  0.4403055226885715   T   T   T
  0.0644925684256396  0.4991640065587024  0.4839528006760478   T   T   T
  0.7067156391182440  0.8277240629360844  0.5130051754416428   T   T   T
  0.7988236910497837  0.8415803443599996  0.4655792983481067   T   T   T
```

20) CO-Na_2O-Co(211)-1x3

```
Co(211)-3x3
   1.00000000000000
     6.0968000000000000    0.0000000000000000    0.0000000000000000
     0.0000000000000000    7.4669999999999996    0.0000000000000000
     0.0000000000000000    0.0000000000000000   23.6221999999999994
   Co   O    Na   C
   48    2    2    1
Selective dynamics
Direct
  0.0039768403096687  0.1666663988214765  0.9745822997011331   F   F   F
  0.0039765122687285  0.5000000000000000  0.9745822997011331   F   F   F
  0.0039768403096687  0.8333336011785235  0.9745822997011331   F   F   F
  0.3315888662905095  0.0000000000000000  0.0034278771663878   F   F   F
  0.3315855858811219  0.3333350743270387  0.0034291471581795   F   F   F
  0.3315855858811219  0.6666647917503710  0.0034291471581795   F   F   F
  0.6664853693741009  0.1666655952859273  0.0285661792720404   F   F   F
  0.6664884857630327  0.5000000000000000  0.0285654596100287   F   F   F
  0.6664853693741009  0.8333344047140727  0.0285661792720404   F   F   F
  0.9983825941477491  0.0000000000000000  0.0609119811025209   F   F   F
  0.9983791497178913  0.3333345386366702  0.0609079171288016   F   F   F
  0.9983791497178913  0.6666654613633298  0.0609079171288016   F   F   F
  0.3314264860254568  0.1666650595955517  0.0918800111759310   F   F   F
  0.3314281262301506  0.5000000000000000  0.0918805615056968   F   F   F
  0.3314264860254568  0.8333349404044483  0.0918800111759310   F   F   F
  0.6654751673008832  0.0000000000000000  0.1205095207050988   F   F   F
  0.6654631938065876  0.3333330654881479  0.1205040597404121   F   F   F
  0.6654631938065876  0.6666669345118521  0.1205040597404121   F   F   F
  0.0009121178323070  0.1666669345118521  0.1526111031148716   F   F   F
  0.0009114617504267  0.5000000000000000  0.1526112301140472   F   F   F
  0.0009121178323070  0.8333330654881479  0.1526111031148716   F   F   F
  0.3340214538774404  0.0000000000000000  0.1833969740329024   F   F   F
  0.3340229300616713  0.3333327976429601  0.1833968470337197   F   F   F
  0.3340229300616713  0.6666672023570399  0.1833968470337197   F   F   F
  0.6658645422707269  0.1660374172918767  0.2117900980960461   T   T   T
  0.6667366244340689  0.5004382308647629  0.2118554643162825   T   T   T
  0.6648505018371085  0.8330335876520153  0.2119251929010326   T   T   T
  0.9976500271947548 -0.0009152142789934  0.2434567089120533   T   T   T
  0.9994419541752373  0.3336727498136443  0.2433463717582243   T   T   T
```

```
 0.9986802249148526  0.6672305147521883  0.2433768180795012   T   T   T
 0.3325205505817793  0.1656167447402343  0.2744783234707141   T   T   T
 0.3323202207837498  0.5005203464497577  0.2741292923054650   T   T   T
 0.3313032653954628  0.8344843688483434  0.2754030764850086   T   T   T
 0.6653438035873240 -0.0001451086055465  0.3030014726379174   T   T   T
 0.6664557620174970  0.3336835320680231  0.3023131973640921   T   T   T
 0.6646856522760883  0.6668688022120725  0.3030835601043886   T   T   T
-0.0003733117933651  0.1676819447939709  0.3335882412674660   T   T   T
 0.9983961010769162  0.5012002018953704  0.3349992166482182   T   T   T
-0.0028299828258668  0.8334750718135748  0.3365147381389103   T   T   T
 0.3290327276351646  0.0015081270937850  0.3681854646085312   T   T   T
 0.3303835492779598  0.3325073003050363  0.3632174238355844   T   T   T
 0.3302292419764821  0.6686320756678014  0.3637227706046005   T   T   T
 0.6673245832511625  0.1685238972889263  0.3898079691366706   T   T   T
 0.6630032573437643  0.4975559461748061  0.3897105084566692   T   T   T
 0.6607745510081795  0.8346430522056604  0.3933386399598129   T   T   T
 0.9834259385443012  0.0038732714809830  0.4230745867549848   T   T   T
 0.9955426878540420  0.3325118661102236  0.4238595857025066   T   T   T
 0.9780984236133625  0.6590038108232589  0.4304189966252635   T   T   T
 0.1171919195741866  0.4895854190147851  0.4757554388491282   T   T   T
 0.7185608969788112  0.8439948225265722  0.5142339662466496   T   T   T
 0.3681024528313128  0.7316150541463173  0.4908880157680375   T   T   T
 0.3889793510931684  0.2640342996130064  0.4860592201200274   T   T   T
 0.8139449779826161  0.8430928781916557  0.4676575895445056   T   T   T
```

21) C_2H_4-Co(111)-3x3

```
Co C H
   1.00000000000000
     7.4587998390197754    0.0000000000000000    0.0000000000000000
    -3.7294002959821051    6.4595099249780574    0.0000000000000000
    -0.0000010106117090   -0.0000017504309440   23.1201000213622159
   Co   C    H
    36    2    4
Selective dynamics
Direct
 0.0000352770987675  0.9999648825964869  0.0907110695049838   F   F   F
 0.3333557427970177  0.0000001793936022  0.0907175141137841   F   F   F
 0.6666918534736581  0.9999938321653516  0.0907267268766958   F   F   F
 0.0000062578975388  0.3333084372988537  0.0907267268766958   F   F   F
 0.3333357007148621  0.3333426504206471  0.0907265971194704   F   F   F
 0.6666945969032270  0.3333051862794676  0.0907313116319486   F   F   F
 0.9999998575876035  0.6666444062796870  0.0907175141137841   F   F   F
 0.3333385397113773  0.6666614354387903  0.0907302303217463   F   F   F
 0.6666573639132167  0.6666642220269168  0.0907265971194704   F   F   F
 0.1111175321760314  0.2222028177229376  0.1750651163386081   F   F   F
 0.4444257597051688  0.2222195372577005  0.1750655488626904   F   F   F
 0.7777781372906531  0.2222218594174734  0.1750735938105805   F   F   F
 0.1111123692640206  0.5555522552209098  0.1750674087162309   F   F   F
 0.4444440697289238  0.5555561254847916  0.1750719502190776   F   F   F
 0.7777804782179913  0.5555742383122606  0.1750655488626904   F   F   F
 0.1111199658994551  0.8888800192425492  0.1750494157144900   F   F   F
 0.4444476042812084  0.8888872953412061  0.1750674087162309   F   F   F
 0.7777970141657633  0.8888823414043898  0.1750651163386081   F   F   F
```

```
0.2242657109236099  0.1147251015225590  0.2662406435787559   T   T   T
0.5559152728827738  0.1114184951329684  0.2649560784399888   T   T   T
0.8893507309390879  0.1112338652890922  0.2649449497427928   T   T   T
0.2231657041214168  0.4426849531089395  0.2661203608252982   T   T   T
0.5534008336331755  0.4427252045008263  0.2661080749670763   T   T   T
0.8883266731557761  0.4426464547152027  0.2657269036039966   T   T   T
0.2226238104384674  0.7779303986206146  0.2650163407038440   T   T   T
0.5556129618196056  0.7774697063439479  0.2647568674476912   T   T   T
0.8889388590301562  0.7777916307773853  0.2650344723436049   T   T   T
0.9972988004568093  0.9960997306624617  0.3489202525924624   T   T   T
0.3326711423815232  0.9959869578779580  0.3489016987708813   T   T   T
0.6664550229248549  0.9989413139613537  0.3485803938991679   T   T   T
0.0003128461354784  0.3321571013386502  0.3499255771399150   T   T   T
0.3320593871651816  0.3298816375976920  0.3569928226272741   T   T   T
0.6658598791339840  0.3320507389521455  0.3499120630842615   T   T   T
0.0000748973193884  0.6661571542581171  0.3493934096418259   T   T   T
0.3343202682512865  0.6680272310599088  0.3489932784145139   T   T   T
0.6676973121139330  0.6680096907992878  0.3490000993260212   T   T   T
0.4284265155475125  0.3309302722960558  0.4414517587070718   T   T   T
0.2372048197215636  0.3306761892232316  0.4414734361550463   T   T   T
0.2371699331481144  0.4724245277888496  0.4536418322165825   T   T   T
0.0955770535965005  0.1886534700467737  0.4535871163198738   T   T   T
0.5700033723657081  0.4728383250935478  0.4536239012162485   T   T   T
0.4284399043032906  0.1891398594818928  0.4535802151577402   T   T   T
```

22) C_2H_4-PrO_2-Co(111)-3x3

```
Co Pr O C H
   1.00000000000000
     7.4587998390197754    0.0000000000000000    0.0000000000000000
    -3.7294002959821051    6.4595099249780574    0.0000000000000000
    -0.0000010106117090   -0.0000017504309440   23.1201000213622159
   Co   Pr   O   C   H
    36    1    2    2    4
Selective dynamics
Direct
  0.0000353225881327  0.9999648942106703  0.0907110695049838   F   F   F
  0.3333557427970177  0.0000001793936022  0.0907175141137841   F   F   F
  0.6666917648931943  0.9999938437795350  0.0907267268766958   F   F   F
  0.0000062578975388  0.3333084372988537  0.0907267268766958   F   F   F
  0.3333357007148621  0.3333426504206471  0.0907265971194704   F   F   F
  0.6666945969032270  0.3333051862794676  0.0907313116319486   F   F   F
  0.9999998791701969  0.6666444062796870  0.0907175141137841   F   F   F
  0.3333385397113773  0.6666614354387903  0.0907302303217463   F   F   F
  0.6666573639132167  0.6666642220269168  0.0907265971194704   F   F   F
  0.1111175321760314  0.2222028177229376  0.1750651163386081   F   F   F
  0.4444257597051688  0.2222195372577005  0.1750655488626904   F   F   F
  0.7777781372906531  0.2222218594174734  0.1750735938105805   F   F   F
  0.1111123692640206  0.5555522552209098  0.1750674087162309   F   F   F
  0.4444440697289238  0.5555561254847916  0.1750719502190776   F   F   F
  0.7777804782179913  0.5555742383122606  0.1750655488626904   F   F   F
  0.1111199658994551  0.8888800192425492  0.1750494157144900   F   F   F
  0.4444476042812084  0.8888872953412061  0.1750674087162309   F   F   F
  0.7777970141657633  0.8888823414043898  0.1750651163386081   F   F   F
```

0.2209980464308831 0.1101994349018439 0.2643889192036905 T T T
0.5571728998362104 0.1160001257901519 0.2663384997830477 T T T
0.8892692583318079 0.1126869649624241 0.2647303396849227 T T T
0.2174945364062416 0.4471494546774587 0.2630309179290135 T T T
0.5603716516117682 0.4425085633511537 0.2646716898292288 T T T
0.8847515255389152 0.4432257747752574 0.2685312444473849 T T T
0.2196055993189893 0.7760230650943726 0.2658843629749298 T T T
0.5552148444801831 0.7788503566162986 0.2653015789961129 T T T
0.8893161660892615 0.7776163425244919 0.2651935432038988 T T T
0.9964786908201744 0.9965446779370216 0.3505508526578509 T T T
0.3355403809997986 -0.0015604060269711 0.3491398114134163 T T T
0.6693899748280869 0.0023834430708399 0.3488177242657231 T T T
0.0041395945005226 0.3335035398073463 0.3510107938094524 T T T
0.3386463232875401 0.3427986816300322 0.3440863593790486 T T T
0.6681912492762042 0.3305418403298164 0.3676496887915006 T T T
0.0000151513921072 0.6729164347542672 0.3528951763938990 T T T
0.3408136279672705 0.6730025336831724 0.3522819889174637 T T T
0.6679794863839531 0.6662159739785040 0.3478501928347509 T T T
0.3058716848368546 0.2222135229001010 0.4506349538699512 T T T
0.2257725315690227 0.4499629951014685 0.4099864774721197 T T T
0.1179135352789193 0.9059269360625436 0.4128224797893326 T T T
0.7615154663577275 0.4542228494073270 0.4477574044383761 T T T
0.6995290885957068 0.2372066396317101 0.4481518879008186 T T T
0.6712986255416902 0.5147344354222005 0.4701991526925626 T T T
0.9259530317460827 0.5676923488800137 0.4531614619070097 T T T
0.5648201084523778 0.1191885474610434 0.4729009363651036 T T T
0.8220946084255338 0.1977725575493214 0.4518552046343371 T T T

23) C_2H_4-Co(211)-1x3

Co C H
1.0000000000000000
6.0968000000000000 0.0000000000000000 0.0000000000000000
0.0000000000000000 7.4669999999999996 0.0000000000000000
0.0000000000000000 0.0000000000000000 23.6221999999999994
Co C H
48 2 4
Selective dynamics
Direct
0.0039768403096687 0.1666663988214836 0.9745822997011260 F F F
0.0039765122687285 0.5000000000000000 0.9745822997011260 F F F
0.0039768403096687 0.8333336011785164 0.9745822997011260 F F F
0.3315888662905095 0.0000000000000000 0.0034278771663949 F F F
0.3315855858811148 0.3333350743270387 0.0034291471581795 F F F
0.3315855858811148 0.6666647917503710 0.0034291471581795 F F F
0.6664853693741009 0.1666655952859273 0.0285661792720404 F F F
0.6664884857630256 0.5000000000000000 0.0285654596100287 F F F
0.6664853693741009 0.8333344047140727 0.0285661792720404 F F F
0.9983825941477491 0.0000000000000000 0.0609119811025209 F F F
0.9983791497178842 0.3333345386366702 0.0609079171288016 F F F
0.9983791497178842 0.6666654613633298 0.0609079171288016 F F F
0.3314264860254568 0.1666650595955517 0.0918800111759310 F F F
0.3314281262301506 0.5000000000000000 0.0918805615057039 F F F
0.3314264860254568 0.8333349404044483 0.0918800111759310 F F F

0.6654751673008761	0.0000000000000000	0.1205095207050988	F	F	F
0.6654631938065876	0.3333330654881479	0.1205040597404121	F	F	F
0.6654631938065876	0.6666669345118521	0.1205040597404121	F	F	F
0.0009121178323070	0.1666669345118521	0.1526111031148645	F	F	F
0.0009114617504267	0.5000000000000000	0.1526112301140472	F	F	F
0.0009121178323070	0.8333330654881479	0.1526111031148645	F	F	F
0.3340214538774404	0.0000000000000000	0.1833969740329024	F	F	F
0.3340229300616713	0.3333327976429601	0.1833968470337197	F	F	F
0.3340229300616713	0.6666672023570399	0.1833968470337197	F	F	F
0.6659255956148380	0.1668387306927896	0.2118315399214045	T	T	T
0.6653797029869145	0.5001838278721612	0.2118774771242978	T	T	T
0.6658769680766116	0.8336309042963843	0.2118161480787779	T	T	T
0.9984971450770040	0.0000574930443549	0.2427842079845912	T	T	T
0.9986445787685340	0.3335318149466585	0.2426302729555652	T	T	T
0.9984413789950910	0.6667999211793522	0.2426858600025979	T	T	T
0.3333571390927925	0.1668517437295304	0.2746159989675913	T	T	T
0.3331810360535596	0.5000418677656948	0.2748497977692068	T	T	T
0.3329914746820734	0.8335033693635592	0.2745832478074934	T	T	T
0.6678810436205741	0.0005282621480566	0.3033322433560609	T	T	T
0.6671233901051468	0.3338943697410655	0.3031890412687036	T	T	T
0.6671154769249358	0.6676688706018699	0.3031573217065783	T	T	T
-0.0006674880643545	0.1672270440010111	0.3342881213233195	T	T	T
-0.0016085515181861	0.5012763576783755	0.3343507484806075	T	T	T
-0.0000178191281885	0.8334978938004244	0.3347584239254892	T	T	T
0.3323622929447293	0.0002567776546732	0.3657842483976530	T	T	T
0.3305193357987513	0.3327872460604484	0.3655628744050484	T	T	T
0.3300943191476738	0.6686190141794206	0.3659173082467139	T	T	T
0.6684556918528178	0.1674027253918159	0.3913955608848111	T	T	T
0.6625703267113854	0.5011223272635696	0.3903764311001696	T	T	T
0.6693267388840644	0.8341963672512018	0.3917399749298996	T	T	T
-0.0008281108981059	0.0016259692177587	0.4205329358204484	T	T	T
0.9875622956403196	0.3334631624437212	0.4235863539587836	T	T	T
0.9898947609429594	0.6628118333604518	0.4224832592435809	T	T	T
0.0197522740231572	0.4870416862683511	0.4968718156490633	T	T	T
0.0999041887092184	0.3063038385448135	0.5024307191912123	T	T	T
0.8665537210404497	0.5207644437269070	0.5184790034283027	T	T	T
0.1451789543060471	0.5952989928720477	0.4989389199443363	T	T	T
0.0067371951531023	0.2121398240844097	0.5284802670913448	T	T	T
0.2765961606817176	0.2823001321281038	0.5014026261389113	T	T	T

24) C_2H_4-PrO_2-Co(211)-1x3

Co Pr O C H
1.00000000000000

6.0968000000000000	0.0000000000000000	0.0000000000000000
0.0000000000000000	7.4669999999999996	0.0000000000000000
0.0000000000000000	0.0000000000000000	23.6221999999999994

Co	Pr	O	C	H
48	1	2	2	4

Selective dynamics
Direct

0.0039768400000000	0.1666663990000004	0.9745823000000016	F	F	F
0.0039765120000013	0.5000000000000000	0.9745823000000016	F	F	F
0.0039768400000000	0.8333336009999996	0.9745823000000016	F	F	F

0.3315888659999970	0.0000000000000000	0.0034278770000000	F	F	F
0.3315855860000028	0.3333350739999972	0.0034291469999985	F	F	F
0.3315855860000028	0.6666647919999988	0.0034291469999985	F	F	F
0.6664853690000001	0.1666655949999978	0.0285661790000020	F	F	F
0.6664884859999987	0.5000000000000000	0.0285654600000029	F	F	F
0.6664853690000001	0.8333344050000022	0.0285661790000020	F	F	F
0.9983825939999988	0.0000000000000000	0.0609119809999967	F	F	F
0.9983791499999981	0.3333345389999991	0.0609079170000015	F	F	F
0.9983791499999981	0.6666654610000009	0.0609079170000015	F	F	F
0.3314264859999980	0.1666650599999997	0.0918800110000006	F	F	F
0.3314281259999987	0.5000000000000000	0.0918805620000001	F	F	F
0.3314264859999980	0.8333349400000003	0.0918800110000006	F	F	F
0.6654751669999968	0.0000000000000000	0.1205095210000025	F	F	F
0.6654631939999973	0.3333330649999979	0.1205040600000018	F	F	F
0.6654631939999973	0.6666669350000021	0.1205040600000018	F	F	F
0.0009121180000022	0.1666669350000021	0.1526111029999981	F	F	F
0.0009114619999977	0.5000000000000000	0.1526112299999980	F	F	F
0.0009121180000022	0.8333330649999979	0.1526111029999981	F	F	F
0.3340214540000019	0.0000000000000000	0.1833969739999972	F	F	F
0.3340229300000033	0.3333327980000007	0.1833968469999974	F	F	F
0.3340229300000033	0.6666672019999993	0.1833968469999974	F	F	F
0.6645038679418933	0.1666940828473601	0.2117536149345588	T	T	T
0.6668350980161388	0.4999274581149993	0.2118850651104055	T	T	T
0.6666925967495559	0.8345085471319806	0.2116843195144681	T	T	T
0.9971402464938849	0.0018831507653378	0.2433410364505671	T	T	T
0.9991928755267638	0.3331416442830909	0.2431702507789253	T	T	T
0.0008537951422136	0.6668538866507475	0.2429867945952075	T	T	T
0.3309792965735021	0.1668836570767019	0.2762180080254098	T	T	T
0.3354938905205690	0.4977321036000936	0.2750810653308575	T	T	T
0.3328659922245464	0.8353508127889034	0.2731939475909700	T	T	T
0.6652536569758811	0.0021836096516317	0.3033413720717101	T	T	T
0.6682678429565183	0.3294490379773405	0.3024289597817802	T	T	T
0.6679892472703765	0.6679349043341912	0.3021192465686219	T	T	T
0.9965622463935024	0.1649853497978349	0.3367881522626946	T	T	T
0.0046695673829341	0.4967309628013341	0.3347662478787963	T	T	T
0.9990759922421457	0.8341797943545636	0.3341583858793399	T	T	T
0.3290719607093087	0.9949971320067698	0.3656680967764716	T	T	T
0.3237480608560939	0.3314327535249652	0.3698997535807703	T	T	T
0.3292654083547463	0.6733201621147146	0.3621776736307801	T	T	T
0.6665080733917150	0.1624685163243748	0.3960146142925514	T	T	T
0.6701042555922035	0.5001995875147793	0.3874395505083134	T	T	T
0.6692529831814400	0.8377081214686597	0.3907932855460702	T	T	T
0.0025664233099282	0.9943275746622604	0.4272845971575843	T	T	T
0.9922653008024535	0.3348866262701052	0.4282177828007835	T	T	T
0.9751474400853127	0.6664751578039481	0.4210454795279717	T	T	T
0.3991738023863956	0.6092152523544858	0.4802479845734832	T	T	T
0.5112790969425469	0.3452239070094069	0.4413606668151314	T	T	T
0.0507852023145833	0.5234423790341352	0.4843074240083908	T	T	T
0.0113607856367539	0.1124415743390720	0.5055094849266789	T	T	T
0.1364485176296854	0.9503261775230339	0.5047320090506463	T	T	T
0.8478033399128052	0.1115665824968311	0.5243567157988805	T	T	T
0.0989325875189479	0.2386892141042800	0.5124338374812948	T	T	T
0.0719028159203490	0.8320885251454846	0.5264880555014494	T	T	T
0.3159513489639311	0.9670478813435246	0.5044756896820537	T	T	T

25) C-Co(111)-3x3

```
Co  C
   1.00000000000000
     7.4587998390197754    0.0000000000000000    0.0000000000000000
    -3.7294002959821051    6.4595099249780574    0.0000000000000000
    -0.0000010106117090   -0.0000017504309440   23.1201000213622159
   Co   C
    36     1
Selective dynamics
Direct
  0.0000353225881327  0.9999648942106703  0.0907110695049766   F   F   F
  0.3333557427970177  0.0000001793936022  0.0907175141137770   F   F   F
  0.6666917648932014  0.9999938437795421  0.0907267268767029   F   F   F
  0.0000062578975388  0.3333084372988466  0.0907267268767029   F   F   F
  0.3333357007148621  0.3333426504206471  0.0907265971194704   F   F   F
  0.6666945969032270  0.3333051862794676  0.0907313116319486   F   F   F
  0.9999998791701969  0.6666444062796870  0.0907175141137770   F   F   F
  0.3333385397113773  0.6666614354387903  0.0907302303217534   F   F   F
  0.6666573639132167  0.6666642220269168  0.0907265971194704   F   F   F
  0.1111175321760314  0.2222028177229376  0.1750651163386081   F   F   F
  0.4444257597051688  0.2222195372577005  0.1750655488626904   F   F   F
  0.7777781372906531  0.2222218594174734  0.1750735938105805   F   F   F
  0.1111123692640206  0.5555522552209098  0.1750674087162309   F   F   F
  0.4444440697289167  0.5555561254847916  0.1750719502190776   F   F   F
  0.7777804782179913  0.5555742383122606  0.1750655488626904   F   F   F
  0.1111199658994622  0.8888800192425492  0.1750494157144900   F   F   F
  0.4444476042812084  0.8888872953412132  0.1750674087162309   F   F   F
  0.7777970141657633  0.8888823414043898  0.1750651163386081   F   F   F
  0.2223380483586267  0.1110500095691979  0.2651516163289674   T   T   T
  0.5554206816364260  0.1110515283190738  0.2651482880174993   T   T   T
  0.8889000229387710  0.1110743295683056  0.2648034854455156   T   T   T
  0.2223365425742542  0.4445771236207265  0.2651514672032683   T   T   T
  0.5555696563705191  0.4444293555622172  0.2658019485868975   T   T   T
  0.8889515532443769  0.4445790127157901  0.2651484733809658   T   T   T
  0.2222352779195763  0.7777691773053237  0.2654273427930417   T   T   T
  0.5553996418133776  0.7776542508696962  0.2651506072167233   T   T   T
  0.8889513869162673  0.7776577599423808  0.2651479573802301   T   T   T
 -0.0001702775825675  0.0001792110146804  0.3488355446336508   T   T   T
  0.3314056147795360  0.9960708055513404  0.3497036279100241   T   T   T
  0.6670675321415461  0.0001802467159488  0.3488330780707654   T   T   T
 -0.0001877346036965  0.3329206668981638  0.3488348935430982   T   T   T
  0.3228626550073723  0.3280765689617099  0.3519525339573998   T   T   T
  0.6719323386306690  0.3280843201298417  0.3519480193148877   T   T   T
  0.0039344014930955  0.6685961128323376  0.3497000814908069   T   T   T
  0.3314090690987684  0.6685736592914326  0.3497055892499839   T   T   T
  0.6719227232748884  0.6771492142131471  0.3519486091983463   T   T   T
  0.5555867361784330  0.4444637285096601  0.3928993102213063   T   T   T
```

26) C-PrO_2-Co(111)-3x3

```
Co Pr C
   1.00000000000000
```

7.4587998390197754 0.0000000000000000 0.0000000000000000
-3.7294002959821051 6.4595099249780574 0.0000000000000000
-0.0000010106117090 -0.0000017504309440 23.1201000213622159
Co Pr O C
36 1 2 1
Selective dynamics
Direct
0.0000353225881327 0.9999648942106703 0.0907110695049766 F F F
0.3333557427970177 0.0000001793936022 0.0907175141137770 F F F
0.6666917648932014 0.9999938437795421 0.0907267268767029 F F F
0.0000062578975388 0.3333084372988466 0.0907267268767029 F F F
0.3333357007148621 0.3333426504206471 0.0907265971194704 F F F
0.6666945969032270 0.3333051862794676 0.0907313116319486 F F F
0.9999998791701969 0.6666444062796870 0.0907175141137770 F F F
0.3333385397113773 0.6666614354387903 0.0907302303217534 F F F
0.6666573639132167 0.6666642220269168 0.0907265971194704 F F F
0.1111175321760314 0.2222028177229376 0.1750651163386081 F F F
0.4444257597051688 0.2222195372577005 0.1750655488626904 F F F
0.7777781372906531 0.2222218594174734 0.1750735938105805 F F F
0.1111123692640206 0.5555522552209098 0.1750674087162309 F F F
0.4444440697289167 0.5555561254847916 0.1750719502190776 F F F
0.7777804782179913 0.5555742383122606 0.1750655488626904 F F F
0.1111199658994622 0.8888800192425492 0.1750494157144900 F F F
0.4444476042812084 0.8888872953412132 0.1750674087162309 F F F
0.7777970141657633 0.8888823414043898 0.1750651163386081 F F F
0.2218311591948475 0.1117782956357841 0.2658927108738715 T T T
0.5520512691347320 0.1087812062587375 0.2640215454555646 T T T
0.8880998421085202 0.1115113265673815 0.2648052409722573 T T T
0.2181074737776463 0.4452995232789953 0.2641144692151559 T T T
0.5567065898040164 0.4446309801731888 0.2634300597465954 T T T
0.8880566485227415 0.4446277832635925 0.2659673855499142 T T T
0.2184466051679525 0.7754253325626249 0.2668619563035719 T T T
0.5511384030787515 0.7751132443535852 0.2651419494566352 T T T
0.8884637831048160 0.7760784028668397 0.2656912620923639 T T T
0.9951435056618591 0.9965923396727504 0.3509055192278500 T T T
0.3341710525199230 0.9954714925993028 0.3513399207434024 T T T
0.6643910092134667 0.9974657412287999 0.3482107449411130 T T T
0.9905080561891485 0.3270842645988612 0.3521765925436749 T T T
0.3396942319454552 0.3434452599255863 0.3486861777846760 T T T
0.6684940519241113 0.3238963528559608 0.3505221185128853 T T T
0.9977726539832280 0.6704269371463879 0.3541421147737177 T T T
0.3338816009647281 0.6707543357552881 0.3524058768567013 T T T
0.6698492751024521 0.6749375720923321 0.3508546773564742 T T T
0.2649230726534089 0.2120761078475647 0.4554303012850158 T T T
0.2018562951935000 0.4433604389839184 0.4096996066621731 T T T
0.1178049541586798 0.9004060786490327 0.4125482736580846 T T T
0.5817109227011624 0.4528338648051564 0.3978203662973674 T T T

27) C-Na_2O-Co(111)-3x3

Co O Na C
1.00000000000000
7.4587998390197754 0.0000000000000000 0.0000000000000000
-3.7294002959821051 6.4595099249780574 0.0000000000000000

-0.0000010106117090 -0.0000017504309440 23.1201000213622159
Co O Na C
36 1 2 1
Selective dynamics
Direct
0.0000353225881327 0.9999648942106703 0.0907110695049766 F F F
0.3333557427970177 0.0000001793936022 0.0907175141137770 F F F
0.6666917648932014 0.9999938437795421 0.0907267268767029 F F F
0.0000062578975388 0.3333084372988466 0.0907267268767029 F F F
0.3333357007148621 0.3333426504206471 0.0907265971194704 F F F
0.6666945969032270 0.3333051862794676 0.0907313116319486 F F F
0.9999998791701969 0.6666444062796870 0.0907175141137770 F F F
0.3333385397113773 0.6666614354387903 0.0907302303217534 F F F
0.6666573639132167 0.6666642220269168 0.0907265971194704 F F F
0.1111175321760314 0.2222028177229376 0.1750651163386081 F F F
0.4444257597051688 0.2222195372577005 0.1750655488626904 F F F
0.7777781372906531 0.2222218594174734 0.1750735938105805 F F F
0.1111123692640206 0.5555522552209098 0.1750674087162309 F F F
0.4444440697289167 0.5555561254847916 0.1750719502190776 F F F
0.7777804782179913 0.5555742383122606 0.1750655488626904 F F F
0.1111199658994622 0.8888800192425492 0.1750494157144900 F F F
0.4444476042812084 0.8888872953412132 0.1750674087162309 F F F
0.7777970141657633 0.8888823414043898 0.1750651163386081 F F F
0.2220037748498481 0.1115485555882207 0.2655055258448769 T T T
0.5559359712201797 0.1105294894759237 0.2647633698581331 T T T
0.8896705835683237 0.1103214467638186 0.2643874871623338 T T T
0.2240601506798727 0.4443746689607073 0.2662616739022490 T T T
0.5537033128261247 0.4424357038389937 0.2652296646644579 T T T
0.8887324712713486 0.4456885498305745 0.2638488297735581 T T T
0.2225217621389413 0.7778236173504700 0.2652024980176155 T T T
0.5561183791343209 0.7780624918752694 0.2655986524347366 T T T
0.8890649005681943 0.7789154017149545 0.2650028063374269 T T T
0.0058164709772389 0.9967811440035191 0.3521955464459429 T T T
0.3345407033212670 0.9994495039772926 0.3491452754586903 T T T
0.6638131432238086 0.0020706430029036 0.3515969818100077 T T T
0.9972523154339522 0.3386025702753698 0.3491402376436824 T T T
0.3410862098954021 0.3345962407368051 0.3534016843204610 T T T
0.6682905611205875 0.3326350853795056 0.3483491293983694 T T T
0.0018466799462907 0.6666606181912436 0.3497773171446861 T T T
0.3356023962023739 0.6703598678329384 0.3530687829710056 T T T
0.6646295371270764 0.6572586630986715 0.3488948820258068 T T T
0.4528424115074486 0.5621723341482251 0.4106274121490990 T T T
0.1458991291032975 0.4395291248821114 0.4579951984285337 T T T
0.7576841110188770 0.7906985023430286 0.4575405778137371 T T T
0.8951752451396521 0.1178376212114946 0.3969377749626274 T T T

28) C-Co(211)-1x3

Co(211)-3x3
1.0000000000000000
6.0968000000000000 0.0000000000000000 0.0000000000000000
0.0000000000000000 7.4669999999999996 0.0000000000000000
0.0000000000000000 0.0000000000000000 23.6221999999999994

```
   Co   C
   48     1
Selective dynamics
Direct
  0.0039768403096687  0.1666663988214836  0.9745822997011260   F   F   F
  0.0039765122687285  0.5000000000000000  0.9745819610366553   F   F   F
  0.0039768403096687  0.8333336011785164  0.9745822997011260   F   F   F
  0.3315888662905095  0.0000000000000000  0.0034278771663949   F   F   F
  0.3315855858811148  0.3333350743270387  0.0034291471581795   F   F   F
  0.3315855858811148  0.6666647917503710  0.0034291471581795   F   F   F
  0.6664853693741009  0.1666655952859273  0.0285661792720404   F   F   F
  0.6664884857630256  0.5000000000000000  0.0285654596100287   F   F   F
  0.6664853693741009  0.8333344047140727  0.0285661792720404   F   F   F
  0.9983825941477491  0.0000000000000000  0.0609119811025209   F   F   F
  0.9983791497178842  0.3333345386366702  0.0609079171288016   F   F   F
  0.9983791497178842  0.6666654613633298  0.0609079171288016   F   F   F
  0.3314264860254568  0.1666650595955517  0.0918800111759310   F   F   F
  0.3314281262301506  0.5000000000000000  0.0918805615057039   F   F   F
  0.3314264860254568  0.8333349404044483  0.0918800111759310   F   F   F
  0.6654751673008761  0.0000000000000000  0.1205095207050988   F   F   F
  0.6654631938065876  0.3333330654881479  0.1205040597404121   F   F   F
  0.6654631938065876  0.6666669345118521  0.1205040597404121   F   F   F
  0.0009121178323070  0.1666669345118521  0.1526111031148645   F   F   F
  0.0009114617504267  0.5000000000000000  0.1526112301140472   F   F   F
  0.0009121178323070  0.8333330654881479  0.1526111031148645   F   F   F
  0.3340214538774404  0.0000000000000000  0.1833969740329024   F   F   F
  0.3340229300616713  0.3333327976429601  0.1833968470337197   F   F   F
  0.3340229300616713  0.6666672023570399  0.1833968470337197   F   F   F
  0.6651836834864367  0.1670241756699727  0.2121787557005698   T   T   T
  0.6653551290437728  0.5000130881130070  0.2120218271879724   T   T   T
  0.6651900106739954  0.8329630275822837  0.2121744677031792   T   T   T
  0.9972328080415268  0.0000066152270395  0.2425129308967935   T   T   T
  0.9983297269388163  0.3341507892971681  0.2427182805003978   T   T   T
  0.9983315371876574  0.6658341425011695  0.2427143291968946   T   T   T
  0.3324577800673333  0.1664097601781237  0.2747655903034971   T   T   T
  0.3331241199194607  0.4999919888602291  0.2744422805143385   T   T   T
  0.3324810389836140  0.8335852469511348  0.2747659202661329   T   T   T
  0.6643907520026215  0.0000068255952465  0.3039697132717603   T   T   T
  0.6672905079228441  0.3339344911607027  0.3033625663218367   T   T   T
  0.6672824856403432  0.6660727410027502  0.3033569694191066   T   T   T
 -0.0026704406428508  0.1654753587475361  0.3330968920647463   T   T   T
  0.0002328669983543  0.4999894400047976  0.3346721302241367   T   T   T
 -0.0026720503707607  0.8345075545028593  0.3330944240998204   T   T   T
  0.3278840689635764 -0.0000003774673127  0.3675056990710739   T   T   T
  0.3341810212159383  0.3332111924702934  0.3650289057677424   T   T   T
  0.3341551568536060  0.6667969758752127  0.3650343529446300   T   T   T
  0.6648317572615734  0.1654293860992728  0.3915219353401320   T   T   T
  0.6604604430964073  0.4999781943597921  0.3938993571935223   T   T   T
  0.6648061086327516  0.8345536393561751  0.3915132344610947   T   T   T
  0.9862308419131331 -0.0000215381943695  0.4196414186557375   T   T   T
  0.0132257522051061  0.3201874704431405  0.4223733261680466   T   T   T
  0.0131448589671364  0.6797592893502391  0.4223662268661467   T   T   T
  0.8577264256830258  0.4999465704763640  0.4500626887079267   T   T   T
```

29) C-PrO_2-Co(211)-1x3

```
Co(211)-3x3
   1.00000000000000
     6.0968000000000000    0.0000000000000000    0.0000000000000000
     0.0000000000000000    7.4669999999999996    0.0000000000000000
     0.0000000000000000    0.0000000000000000   23.6221999999999994
   Co   Pr   O    C
   48    1    2    1
Selective dynamics
Direct
  0.0039768403096687  0.1666663988214765  0.9745822997011331   F   F   F
  0.0039765122687285  0.5000000000000000  0.9745822997011331   F   F   F
  0.0039768403096687  0.8333336011785235  0.9745822997011331   F   F   F
  0.3315888662905095  0.0000000000000000  0.0034278771663878   F   F   F
  0.3315855858811219  0.3333350743270387  0.0034291471581795   F   F   F
  0.3315855858811219  0.6666647917503710  0.0034291471581795   F   F   F
  0.6664853693741009  0.1666655952859273  0.0285661792720404   F   F   F
  0.6664884857630327  0.5000000000000000  0.0285654596100287   F   F   F
  0.6664853693741009  0.8333344047140727  0.0285661792720404   F   F   F
  0.9983825941477491  0.0000000000000000  0.0609119811025209   F   F   F
  0.9983791497178913  0.3333345386366702  0.0609079171288016   F   F   F
  0.9983791497178913  0.6666654613633298  0.0609079171288016   F   F   F
  0.3314264860254568  0.1666650595955517  0.0918800111759310   F   F   F
  0.3314281262301506  0.5000000000000000  0.0918805615056968   F   F   F
  0.3314264860254568  0.8333349404044483  0.0918800111759310   F   F   F
  0.6654751673008832  0.0000000000000000  0.1205095207050988   F   F   F
  0.6654631938065876  0.3333330654881479  0.1205040597404121   F   F   F
  0.6654631938065876  0.6666669345118521  0.1205040597404121   F   F   F
  0.0009121178323070  0.1666669345118521  0.1526111031148716   F   F   F
  0.0009114617504267  0.5000000000000000  0.1526112301140472   F   F   F
  0.0009121178323070  0.8333330654881479  0.1526111031148716   F   F   F
  0.3340214538774404  0.0000000000000000  0.1833969740329024   F   F   F
  0.3340229300616713  0.3333327976429601  0.1833968470337197   F   F   F
  0.3340229300616713  0.6666672023570399  0.1833968470337197   F   F   F
  0.6646579422798129  0.1664127234338406  0.2117747334176793   T   T   T
  0.6657979986372220  0.4997842039222730  0.2119388020351741   T   T   T
  0.6657221584832392  0.8340930578824738  0.2118341186016801   T   T   T
  0.9968876559786963 -0.0000544936298271  0.2432890268429820   T   T   T
 -0.0019255420666949  0.3339311181973192  0.2433260902881982   T   T   T
  0.0003481260475894  0.6665824410356554  0.2429495377698467   T   T   T
  0.3317136711743734  0.1671248175961575  0.2755390373540217   T   T   T
  0.3345576999534116  0.4981509905998547  0.2754092151077924   T   T   T
  0.3315810143970591  0.8339799110526186  0.2737337162740914   T   T   T
  0.6648905315398171  0.0018473417868035  0.3030084900945898   T   T   T
  0.6669167571740654  0.3296021255006357  0.3023121363358954   T   T   T
  0.6655731368409288  0.6674684992350458  0.3020357481165770   T   T   T
  0.9964315355195292  0.1684671024551909  0.3342324829624961   T   T   T
  0.0033513996031114  0.5001131019420725  0.3349380697803835   T   T   T
  0.9966926634306925  0.8321960938931868  0.3350348849175179   T   T   T
  0.3271289452940551  0.9956131632329416  0.3648450865122700   T   T   T
  0.3265666387023504  0.3304931924421498  0.3696424071447743   T   T   T
  0.3248105943413145  0.6724509135259389  0.3636382600404359   T   T   T
  0.6686430754909513  0.1638154590033586  0.3957732472969617   T   T   T
  0.6714961208660476  0.4964449535058141  0.3865363404952531   T   T   T
```

```
  0.6529073982275546  0.8359636206798809  0.3911859104607936   T   T   T
  0.9979768015573773  0.0065381239752620  0.4223591589198578   T   T   T
  0.0019643069072233  0.3321168766854257  0.4259412802248017   T   T   T
  0.9799101016835695  0.6641205640235570  0.4296190706180286   T   T   T
  0.4224615086570892  0.6015398808323551  0.4824064236727929   T   T   T
  0.5193008568252225  0.3429196448161501  0.4413762011409862   T   T   T
  0.0764276318399706  0.4938548935519562  0.4835555458943468   T   T   T
  0.8143935753273973  0.8451092670500731  0.4545985771968322   T   T   T
```

30) C-Na_2O-Co(211)-1x3

```
Co(211)-3x3
   1.00000000000000
     6.0968000000000000    0.0000000000000000    0.0000000000000000
     0.0000000000000000    7.4669999999999996    0.0000000000000000
     0.0000000000000000    0.0000000000000000   23.6221999999999994
   Co   O    Na   C
   48    1    2    1
Selective dynamics
Direct
  0.0039768403096687  0.1666663988214765  0.9745822997011331   F   F   F
  0.0039765122687285  0.5000000000000000  0.9745822997011331   F   F   F
  0.0039768403096687  0.8333336011785235  0.9745822997011331   F   F   F
  0.3315888662905095  0.0000000000000000  0.0034278771663878   F   F   F
  0.3315855858811219  0.3333350743270387  0.0034291471581795   F   F   F
  0.3315855858811219  0.6666647917503710  0.0034291471581795   F   F   F
  0.6664853693741009  0.1666655952859273  0.0285661792720404   F   F   F
  0.6664884857630327  0.5000000000000000  0.0285654596100287   F   F   F
  0.6664853693741009  0.8333344047140727  0.0285661792720404   F   F   F
  0.9983825941477491  0.0000000000000000  0.0609119811025209   F   F   F
  0.9983791497178913  0.3333345386366702  0.0609079171288016   F   F   F
  0.9983791497178913  0.6666654613633298  0.0609079171288016   F   F   F
  0.3314264860254568  0.1666650595955517  0.0918800111759310   F   F   F
  0.3314281262301506  0.5000000000000000  0.0918805615056968   F   F   F
  0.3314264860254568  0.8333349404044483  0.0918800111759310   F   F   F
  0.6654751673008832  0.0000000000000000  0.1205095207050988   F   F   F
  0.6654631938065876  0.3333330654881479  0.1205040597404121   F   F   F
  0.6654631938065876  0.6666669345118521  0.1205040597404121   F   F   F
  0.0009121178323070  0.1666669345118521  0.1526111031148716   F   F   F
  0.0009114617504267  0.5000000000000000  0.1526112301140472   F   F   F
  0.0009121178323070  0.8333330654881479  0.1526111031148716   F   F   F
  0.3340214538774404  0.0000000000000000  0.1833969740329024   F   F   F
  0.3340229300616713  0.3333327976429601  0.1833968470337197   F   F   F
  0.3340229300616713  0.6666672023570399  0.1833968470337197   F   F   F
  0.6660229830382043  0.1664180394931851  0.2118080413153266   T   T   T
  0.6664426563819583  0.5004865387322640  0.2118496763119750   T   T   T
  0.6650819728261590  0.8329395921601445  0.2118653610911357   T   T   T
  0.9982951932438143  0.9994225565787256  0.2432422663639840   T   T   T
  0.9984396644800451  0.3340788787620101  0.2433175370757806   T   T   T
  0.9990398098020749  0.6674718252495289  0.2435036232656308   T   T   T
  0.3330188721966958  0.1656560887983264  0.2744756479918403   T   T   T
  0.3321828815277689  0.5003364737550637  0.2741907694773036   T   T   T
  0.3317471108629577  0.8343049750536432  0.2747477952110169   T   T   T
```

0.6662758850264000	0.9996581887327556	0.3026024407681248	T	T	T
0.6654621338386121	0.3335342855802528	0.3027331869064074	T	T	T
0.6652426871261138	0.6671541227364572	0.3032493960256671	T	T	T
0.9982388363238468	0.1675822064598327	0.3336601089753727	T	T	T
0.9976017636900938	0.5015922075961459	0.3355140442157161	T	T	T
0.9986305562196982	0.8340520750107976	0.3356327237275910	T	T	T
0.3300248634253058	0.0002047020037566	0.3674997441658528	T	T	T
0.3298426701306598	0.3311673307645195	0.3633138532849471	T	T	T
0.3289739607705178	0.6670065843298526	0.3630568908775860	T	T	T
0.6664791600617851	0.1700704161599977	0.3898430881548156	T	T	T
0.6616953667926030	0.4960781099051799	0.3898620023270907	T	T	T
0.6580597562294932	0.8358733027627758	0.3927417749190200	T	T	T
0.9984268947398636	0.0142724128821413	0.4236781404465580	T	T	T
0.9902844231834672	0.3346277470016989	0.4247344140392910	T	T	T
0.9914828848889192	0.6578119681192123	0.4303955930214121	T	T	T
0.1219335239543362	0.4872827295581384	0.4764193173124881	T	T	T
0.3923367510265897	0.7254957157231614	0.4852715219231505	T	T	T
0.3826205856606456	0.2585279674893396	0.4845501869723537	T	T	T
0.8311763562963463	0.8427626796994238	0.4542526628392116	T	T	T
